# GIS based geostatistical modelling and trends analysis of groundwater quality for suitable uses in Dhaka division

**DOI:** 10.1038/s41598-024-66567-z

**Published:** 2024-07-29

**Authors:** Md. Rezaul Karim, Md. Atif Arham, Md. Jahim Uddin Shorif, Amimul Ahsan, Nadhir Al-Ansari

**Affiliations:** 1 Department of Civil and Environmental Engineering, Islamic University of Technology, Dhaka, Bangladesh; 2 Department of Civil and Construction Engineering, Swinburne University of Technology, Melbourne, Australia; 3 Civil, Environmental and Natural Resources Engineering, Lulea University of Technology, 971 87 Lulea, Sweden

**Keywords:** Groundwater, Mann–Kendall Test, Sens slope, Pearson’s correlation matrix, Piper diagram, GIS mapping, Environmental sciences, Hydrology, Solid Earth sciences, Civil engineering

## Abstract

Preserving the quality of groundwater has become Bangladesh’s primary challenge in recent years. This study explores temporal trend variations in groundwater quality on a broader scale across 18 stations within the Dhaka division over 35 years. The data set encompasses an analysis of 15 distinct water quality parameters. Modified Mann-Kendal, Sens Slope and Mann-Kendal tests were performed to determine the trend’s variation and slope. In addition, the spatial–temporal changes in the quality of groundwater are studied through Geographic Information System (GIS) mapping and Piper diagram was applied to identify the unique hydrochemical properties. This is the first study conducted on this area using various trends analysis and no in-depth study is available highlighting the trends analysis of groundwater quality on a larger magnitude. In contrast, the correlation matrix reveals a high association between Mg^2+^ and SO_4_^2−^, Na^+^ and Cl^−^ that affects salinity and overall hardness at the majority of sites. The Piper diagram also demonstrates that the groundwater in Madaripur Sadar has major salinity issues. The analysis reveals a distinctive dominance of bicarbonate (HCO_3_^−^) ions across all sampling stations, with (HCO_3_^−^) equivalent fractions consistently ranging from 0.70 to 0.99 which can cause a significant impact on groundwater uses. This extensive analysis of long-term groundwater quality trends in the Dhaka Division enables researchers to comprehend the overall transition of groundwater quality for hardness related complications in future. Moreover, it can be a baseline study considering the valuable implications and future steps for sustainable water resource management in this region.

## Introduction

Groundwater is an indispensable natural resource that serves various purposes, including drinking, irrigation, and household use. Bangladesh has around 1.8 million hand tube wells for delivering drinking water from subterranean sources to fulfil the needs of 124 million people^1^. On the other hand, Dhaka’s water supply system is primarily reliant on subsurface water sources. In 2008, the Dhaka Water Supply and Sewerage Authority (DWASA) extracted 1.6 Mm^3^ of groundwater per day from 500 wells drilled in the upper and lower Dupi Tila aquifers (the UDTA and the LDTA)^2^. The UDTA is dewatered in major portions and certain areas of the city, the water level is dropping at a pace of up to 3 m/year^3^. Furthermore, land degradation can have a direct impact on soil deterioration, destroying ecosystem functioning and interfering with the hydrological, biological and geochemical cycles^4^. The other reasons for changing and worsening groundwater quality are intensive extraction of groundwater, disposal of industrial and municipal waste water and penetration of dirty surface water into shallow aquifers. The issue of groundwater quality is growing more crucial as a result of population growth, fast industrialisation and increased use of pesticides and fertilizers in agriculture^5^.

The groundwater within this region primarily exhibits a composition characterized by the dominance of Ca^2+^-Mg^2+-^HCO_3_^−^ ions, particularly the HCO_3_^−^ anion. The presence of cations in the groundwater results from processes such as carbonate dissolution, cation exchange, and the weathering of arsenopyrites and silicates. The alkalinity of water was suggested by the high pH values of the samples (8.1–8.3), which may be attributed to the presence of significant levels of salt, calcium, carbonate, magnesium and bicarbonate ions^6^.Saline intrusions mostly determine the sodium content of groundwater, evaporates and silicate minerals. However, Na^+^ and K^+^ are produced through hard rock weathering, particularly silicate weathering^7^. Elevated levels of dissolved substances, especially arsenic, pose significant challenges in different regions^8^.

Groundwater pollution in Dhaka city results from anthropogenic activities, raising concerns about declining water quality^9^. In Munshiganj district, elevated arsenic (As), manganese (Mn), and salinity levels in groundwater cause pollution initially^10^. In Gopalganj, GIS and multivariate statistics-based analysis has shown and confirm the multiple sources contribute to groundwater contamination, including excessive extraction, reduced river flow, and rock weathering leading to seawater intrusion with elevated Na^+^, Mg^2+^, Ca^2+^, and Cl^−^ levels. GIS based analysis have been done in Kashiani and Kotalipara Upazila in Gopalganj face groundwater contamination from factors like human activities, poor drainage, geological influences, and population growth. In this region, the quality of water failed to meet the potable drinking water quality standards due to high concentrations of As and elevated levels of certain elements such as iron (Fe) and manganese (Mn)^11^. Gazipur District grapples with water pollution issues, including coliforms, harmful metals, and pesticides affecting both surface and groundwater. Industries, especially iron and arsenic contamination, pose significant challenges to drinking water quality^12^. In Faridpur district, garbage management problems and the infiltration of small-scale industrial wastewater into groundwater contribute to potential pollution such as Na^+^ and Cl^−^ concentrations in the groundwater samples are positively skewed and considered extreme. Recharge water quality and surrounding pollution sources also influence groundwater chemistry^13^. The most common mineral ingredient that causes alkalinity is calcium carbonate, which may be found in rocks such as limestone or dolomite and calcite can be leached in the soil. Water with a high alkalinity level has a bitter flavour^14^. From a study, it is found that, during droughts, groundwater pumping is likely to trigger up-coning of the saltwater interface into fresh groundwater utilized for irrigation, resulting in water quality degradation^15^. High mineralization levels are often caused by repetitive farming and excessive pumping, which causes groundwater quality to deteriorate and saltwater intrusion^16^. In the realm of groundwater quality trend detection, several well-established methods have gained global prominence. The Mann–Kendall Test, Modified Mann–Kendall Test, and the Sens Slope estimator are widely recognized tools employed worldwide^17^. In Pakistan, these tests play a crucial role in identifying critical trends^18^. Meanwhile, the Variance-Corrected Mann–Kendall test has been applied to identify groundwater quality trends in diverse regions, including India^19^, the Dezful Aquifer^20^, and the East Azerbaijan province in Northwest Iran^21^. Furthermore, the Mann–Kendall Test has demonstrated its high reliability in assessing the long-term sustainability of groundwater resources and quality in various locations, such as the Bacchiglione basin in Veneto, Italy^22^, the Jiroft plain^23^, Oak Ridge, Tennessee^24^, and the Yangtze river basin^25^. These applications underscore the versatility and significance of these tests in comprehensively evaluating groundwater quality trends on a global scale.

A rigorous study has been conducted to ascertain the seasonal limitations for groundwater quality in Bangladesh, taking data from the northwest part of the country^26^. In the Rangpur region, especially within the Tista flood plain, groundwater quality evaluations have also been conducted using indicators including SAR (Sodium Adsorption Ratio), SSP (Sodium Saturation Percentage), and PI (Permeability Index)^27^. In different parts of Bangladesh, groundwater quality has also been assessed using various robust techniques and approaches. These techniques comprise the use of the Groundwater Quality Index (GWQI), Moran’s Spatial Autocorrelation Index, Fuzzy Logic GIS (Geographic Information System), Correlation Matrix (CM), Hazard Index (HI), Carcinogenic Risk (CR), and Stochastic Uncertainty Analysis. These evaluations have advanced knowledge of groundwater quality in Bangladesh’s northeastern and north-central areas^28^, as well as in the southeast coastal region^29^ and Sylhet district^30^. Several laboratory tests and hydrochemical techniques, such as calculating hydraulic conductivity using the equations of Hazen and Kozeny-Carman, have been used in Savar and has plotted in GIS mapping for visualization^31^. The Ashulia-Kashimpur Upper Aquifer, Northwest of Dhaka city, has also been subject to groundwater quality assessment^32,33^. Groundwater quality was assessed in Gazipur city, specifically the potential health hazard of drinking water in restaurants and tea stalls^34^, Hazaribagh, and Gopalganj^35^ District, using drinking water evaluation indices, correlation matrices, irrigation water quality index (IWQI), principal component analysis, Gibbs plot, Ayers and Westcot’s four groups of IWQ parameters, and geostatistical modelling^36,37^. In Dhaka city, the hazard index (HI) and hazard quotient (HQ) were computed to assess the chronic health risk of exposure to metals and anions through oral and cutaneous routes^38,39^. Systematic identification of the primary ions and water quality has been done for the Dupi Tila aquifer^40^. On the other hand, the MODFLOW-2005 groundwater-flow model, hydrostratigraphic analysis, and data estimation based on population and water consumption data were also examined in Dhaka, Gazipur, and Narayanganj^41^. The findings indicate that the predominant groundwater type in Dhaka city is Ca^2+^–Mg^2+^–HCO_3_^−^, with Na^+^  > Mg^2+^  > Ca^2+^  > K^+^ and HCO_3_—> Cl^-^ > SO_4_^2−^ > NO_3_^2−^ as the trends for cations and anions, respectively. The spatial distribution of ions, Na/Cl, and Na/SiO_2_ molar ratios has been analyzed to comprehend the dominating ion^42^. All measured parameters except Na^+^, K^+^, Cl^−^, F^−^, and NO_3_^2−^ are within acceptable limits. Site-specific monitoring of groundwater quality can benefit from the association between EC, Cl^−^, and SO_4_^2−^, which was discovered through regression and correlation analysis^43^.

Previously, the groundwater quality studies only covered the central part of Bangladesh like Dhaka city, Gopalganj and Gazipur areas. In this study, 18 different locations and 15 water quality parameters were analysed in the Dhaka division to observe the overall scenario of groundwater chemistry. Trends analysis was done by following Mann-Kendal test, Modified Mann-Kendal test and Sen’s Slope. The correlation was identified by the Pearson test and the groundwater chemistry is represented by a Piper diagram. Spatial variation was shown through mapping by QGIS. This study aimed to analyse the trend of groundwater quality parameters to obtain the correlation between the parameters in the individual stations. No such study has been found in the literatures for the Dhaka division till now. Hence, it is essential to conduct an inclusive trends analysis of groundwater quality in the region to assess its current status and trends. GIS-based geostatistical modelling and trends analysis of groundwater quality were performed for the first time for potential uses in the Dhaka division. This research can inform Bangladesh’s water management policies, especially in addressing the challenges posed by deterioration of groundwater quality. Understanding groundwater trends allows us for protecting water sourced in better way, ensuring they last for future and maintain the sustainability of ecosystems.

## Description of the study area

### Location and hydrometeorology

Dhaka division covers an area of 31,026.51 km^2^ and is located between 22°51’ and 25°25’ north latitudes and 89°19’ and 91°15’ east longitudes. It is bounded on the north by the Indian state of Meghalaya and the Kurigram district, on the south by Bagerhat, Barisal Pirojpur, and Chandpur districts and on the east by Brahmanbaria, Sunamganj, Habiganj, Comilla and Chandpur districts and on the west by Narail, Pabna, Magura, Kushtia, Jhenaidah, Sirajganj. Dhaka is surrounded by a flat plain bordered by the Meghna, Padma (Ganges) and Jamuna (Brahmaputra) rivers. A network of streams and rivers traverses the plain, the most important of which are the Dhaleswari, Buriganga and Sitalakhya. Dhaka division’s maximum elevation is 359 m and the average elevation is 14 m.

**Figure 1:**
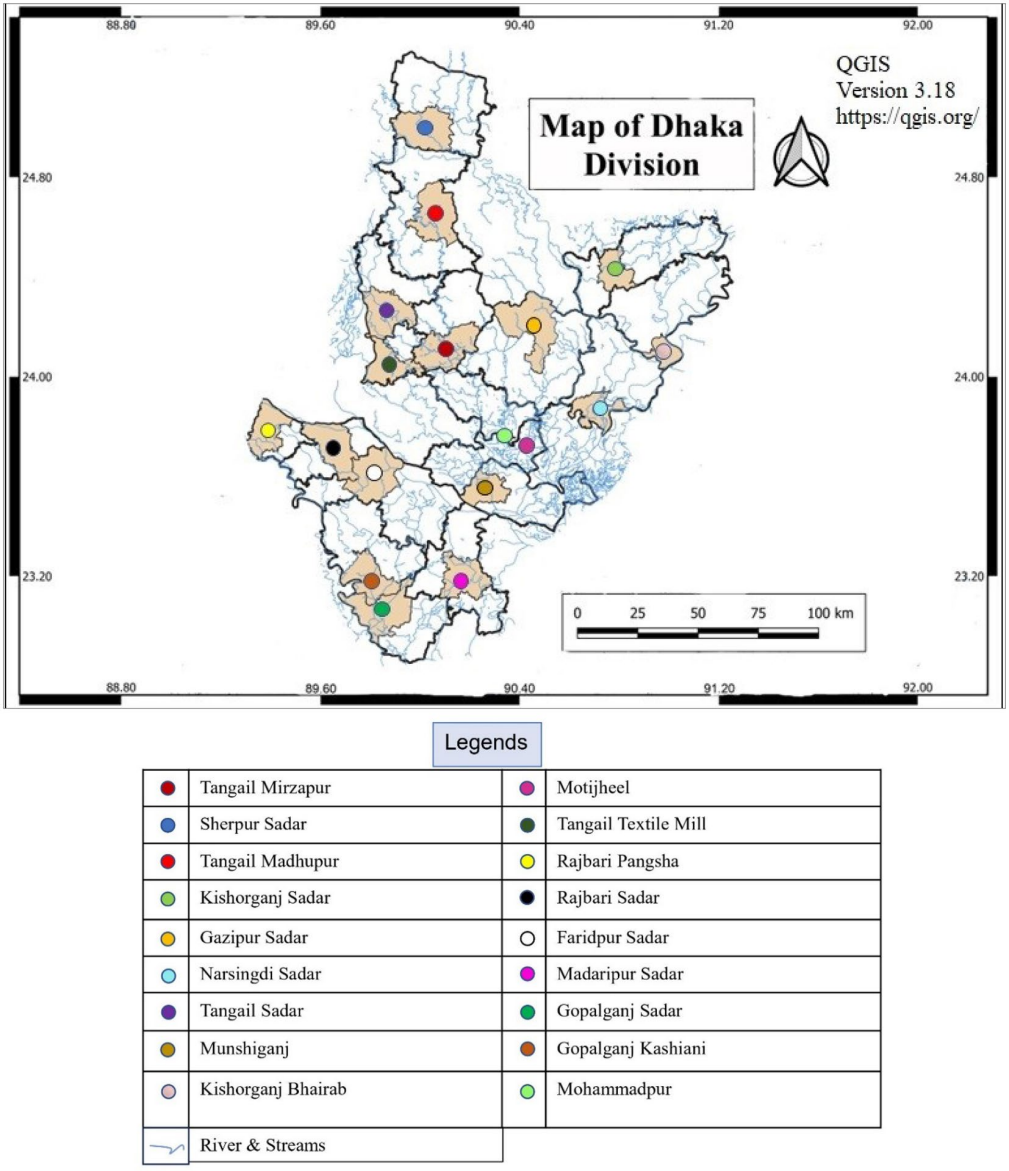
Location of the stations for water quality analysis (QGIS, Version 3.18, https://qgis.org/).

This study has covered 18 different locations in Dhaka division which are Faridpur Sadar, Gazipur (Sreepur), Gopalganj Kashiani, Gopalganj Sadar, Kishoregonj Sadar, Kishoreganj Bhairab, Madaripur Sadar, Mohammadpur, Motijheel, Munshiganj Sreenagar, Narsingdi Sadar, Rajbari (Pangsha), Rajbari Sadar, Sherpur Sadar, Tangail (Madhupur), Tangail Sadar, Tangail (Mirzapur) and Textile Mill Tangail. The locations are shown in Fig. 1. The annual average temperature showed an increasing trend for Dhaka, Mymensingh and Faridpur by 0.02 C, 0.003 C and 0.01 C per year, respectively^44^. Annual total rainfall in Dhaka and Mymensingh increased by 2.330 mm and 2.171 mm each year, respectively. Annual total rainfall decreased by 5.996 mm, 4.179 mm and 15.10 mm per year in Tangail, Faridpur and Madaripur, respectively. Rainfall in Madaripur has decreased drastically and the coefficient of determination was 0.158 and it was observed that the yearly average rainfall in Dhaka was increasing by 2.2 mm per year^45^.

### Physiography, groundwater scenario and land use pattern

Every day, around 2.3 Mm^3^ of water is necessary to meet Dhaka’s demand. To supply this demand of over 14 million people, approximately 2.0 Mm^3^ of groundwater is extracted from the upper Plio-Pleistocene Dupi Tila aquifer. Dhaka Water Supply and Sewerage Authority (DWASA) installed tube wells and over 2000 private tube wells of varying depths have been extracting groundwater from this aquifer. Groundwater extraction is a widespread practice in Bangladesh for irrigation purposes, covering around 4.2 million hectares of land irrigated by both shallow and deep tubewells. Groundwater abstraction for Boro rice cultivation differs between groundwater sellers (owners of tubewells) and groundwater buyers (farmers). On average, groundwater sellers apply 15,300 m^3^/ha of water to Boro rice, while groundwater buyers apply 10,500 m^3^/ha^46^. On the other hand, the level of groundwater development in Dhaka city varies between 117 and 320%, indicating the overexploitation of aquifers in all areas. The excessive extraction of groundwater compared to its recharge has consistently caused a decline in groundwater levels across the study area^47^. The unrestricted abstraction of groundwater has caused issues with the city’s water resource management. Because of uncontrolled development, recharging areas are gradually shrinking. As a result, the process of natural water recharge to the aquifer has not kept pace with the rate of water removal from it during the previous three decades.

Rapid urbanization leads to a greater demand for water in urban areas, encompassing household, industrial, and commercial needs, thereby increasing the extraction of groundwater. As urban areas expand, they encroach upon natural recharge zones and increase impermeable surfaces, which diminishes infiltration and groundwater replenishment, heightening the dependence on groundwater resources^48^. Industrial operations necessitate significant water quantities for manufacturing and cooling purposes, leading to notable groundwater extraction within industrial zones^49^. Additionally, the discharge of industrial wastewater often entails the pumping of groundwater for treatment and disposal, contributing further to groundwater abstraction. When natural vegetation gives way to urban or agricultural land, it disrupts hydrological processes, reducing groundwater recharge rates and heightening the utilization of existing groundwater supplies^50^. Pleistocene alluvium fills the city’s dissected uplands, while alluvium of recent river-borne deposits covers the city’s low-lying flood plains. The Pleistocene Madhupur Clay, characterized by reddish plastic clay with silt and extremely fine sand, overlies the Pliocene Dupi Tila Formation, which forms the primary aquifer. The Dupi Tila Formation is made up of medium to coarse yellowish-brown sand with some gravel. Recent Holocene alluvial floodplain deposits cover the incised channels and depressions. The upper aquifer system (first aquifer) is defined as (1) an upper formation composed of very fine to fine sand, in places associated with traces of silt, that extends down to a depth of 30 to 90 m, (2) a middle part composed of fine to medium sand, in places associated with coarse sand at depth, that extends down to a drilling depth of 100 to 240 m with thicknesses ranging from 50 to more than 200 m^51^.

The first aquifer is recharged primarily by horizontal flow from adjacent regions, with a part from vertical percolation of rain and floodwater. The Dupi Tila Aquifer is recharged by topographically induced vertical leaking through the Madhupur Clay^52,53^. The rechargeable surface area is shrinking day by day as a result of the unplanned urbanization of buildings, roads and concrete pavements. Furthermore, the compact top clay layer in the subsurface prevents vertical recharging, despite the fact that the average annual rainfall in the metropolitan region is around 1800 mm. Lowering the water table lengthens flow routes, which increases the time necessary for recharging and diminishes vertical hydraulic conductivity owing to pore drying^54^.

## Methodology

### Data collection

Groundwater data for this research were collected from 18 stations; the locations of the stations are indicated in Fig. 1. The groundwater quality data for 35 year period (1985–2019) were gathered from the Bangladesh Water Development Board. The samples were chemically examined by the department to ascertain the following water quality parameters e.g., nitrate (NO_3_^−^), EC, silica (SiO_2_^2+^), pH, sodium (Na^+^), chloride (Cl^−^), carbonate (CO_3_^2−^), magnesium (Mg^2+^), potassium (K^+^), iron (Fe^2+^), sulphate (SO_4_^2−^), bicarbonate (HCO_3_^-^), total dissolved solids (TDS), calcium (Ca^2+^) and hardness. On the GIS platform, these data were utilised for univariate and multivariate statistical analysis to determine the change in trend according to the time cycle period. Those characteristics are determined for understanding the sources of dissolved constituent salts in water. A key problem of this work was the irregular nature of water quality observations throughout space and time within a district, which results.

in either a lack of acceptable continuous temporal data or an inadequate geographical representation of the study region. For those missing data, mice analysis was used for missing data imputation and minimising the possible errors that could occur due to lack of data availability. To accommodate for changing sample sizes, percentages of observations exceeding environmental criteria were determined, together with descriptive statistics, to support conclusions regarding changes in trend for water quality. Groundwater physicochemical data were examined for water type using Grapher and Pearson correlation using R studio which showed the correlation water quality parameter.

### Mice analysis

Data regarding various parameters were gathered from the Bangladesh Water Development Board. However, certain data points were absent, potentially leading to inconsistencies in the analysis. Consequently, the mice analysis method was employed to address this issue. The RStudio MICE algorithm package was utilized to rectify the inconsistent data.

### Box and whisker plots

Box plots, also known as box and whiskers plots, are highly effective visualizations for comparing the distribution of a numerical variable across multiple sub-samples. They were introduced by Tukey in 1977^55^. The box plot necessitates the determination of the minimum value (q_0_), the three quartiles (q_1_ ≤ q_2_ < q_3_), and the highest value (q_4_) of the variable within the sample. To create a box plot, one should draw a vertical box that spans from the first quartile to the second quartile, which is also known as the median. Furthermore, basic vertical lines are depicted between the minimum and first quartile, as well as between the third quartile and maximum. It is noted that box and whisker plots of the original data utilizing Tukey’s symmetric fences.

### Mann–Kendall test

Mann first proposed the Mann–Kendall (MK) test in 1945 and Kendall expanded on it in 1975. This non-parametric test is commonly used in meteorological and hydrological data sets, to determine linear and non-linear time series patterns. The trend was identified using data from 18 different observation stations. It is a simple method for dealing with the greatest values, missing values, and values below a specified threshold. The test is applicable for non-normal distributions. For the data S statistics, the MK test value is determined as follows:

1 $$ S = \sum\limits_{i = 1}^{n - 1} {\sum\limits_{j = i + 1}^{n} {{\text{sgn}} \left( {Xj - Xi} \right)} } $$

where, x_i_ and x_j_ address respectively data in the i and j years, individually and n addresses the time length of the statistical period. In the next equation, a pair of estimation values were correlated through subtractions with growing values =  + 1, declining values = −1, no change = 0 and are written as:

2 $$ sign\left( {Xj - Xi} \right) = \left\{ {\left\{ \begin{gathered} + 1,\quad if\left( {x_{j} - x_{i} } \right) > 0 \hfill \\ 0,\quad if\left( {x_{j} - x_{i} } \right) = 0 \hfill \\ - 1,\quad if\left( {x_{j} - x_{i} } \right) < 0 \hfill \\ \end{gathered} \right.} \right. $$

The variance of S with a zero mean for n ≥ 8 is denoted as variance. The statistic S can be expressed using the mean (E) and variance (V), as follows:

3 $$\text{V}(\text{S}) =\frac{n\left(n-1\right)\left(2n+5\right)-{\sum }_{i=1}^{m}{t}_{i}\left({t}_{i}-1\right)\left(2{t}_{i}+5\right)}{18}$$

In this equation, the quantity of data is n, the number of ties is m and the number of ties for the i^th^ value is t_i_. In cases when n > 10, the statistic called the standard normal test Z, which corresponds to the normal distribution at the 95 percent and 99 percent confidence interval levels is calculated for the data point as follows:

4 $$Z = \left\{ {\begin{array}{*{20}c}    {\frac{{S - 1}}{{\sqrt {Var\left( s \right)} }}\quad S > 0}  \\    {0\quad S = 0}  \\    {\frac{{S + 1}}{{\sqrt {Var\left( s \right)} }}\quad S < 0}  \\   \end{array} } \right.$$

Positive Z values suggest that trends are increasing, whereas negative Z values indicate that trends are diminishing. The α significance level is used to test trends. When |Z|> Z_1-α/2_, the null hypothesis H_0_ of no trend is rejected and the alternative hypothesis H_1_ of a substantial trend is accepted. The usual normal distribution in the table provides Z_1-α/2_. If the estimated Z-statistics value is less than or greater than the crucial Z-statistics value obtained from the normal distribution of the table, the alternative or null hypothesis is rejected or accepted. The MK test was applied to see if a trend in groundwater quality parameters was statistically significant, with significance thresholds of = 0.05 (or 95% confidence intervals) and = 0.01 (or 99% confidence intervals) in this trend study.

### Modified Mann–Kendall test

The modified VAR(S) statistic may be calculated as follows^55^:

5 $$\text{VAR}(\text{S}) = \left(\frac{n\left(n-1\right)\left(2n+5\right)}{18}\right) \left(\frac{n}{{n}_{e}^{*}}\right)$$

The correction factor (n/n*_e_) is now adjusted to the autocorrelated data as

6 $$ \left( {\frac{n}{{n_{e}^{*} }}} \right) = 1 + \left( {\frac{2}{{n^{2}  - 3n^{2}  + 2n}}} \right)\mathop \sum \limits_{{f = 1}}^{{n - 1}} \left( {n - f} \right)\left( {n - f - 1} \right)\left( {n - f - 2} \right)\rho _{e} \left( f \right) $$

ρ(ƒ) represents the autocorrelation function between ranks of observations and can be estimated as:

7 $$ \rho \left( f \right) = 2{\text{Sin Sin}} \left( {\frac{\Pi }{6}\rho e\left( f \right)} \right) $$

### Sen’s slope

In this study, Theil–Sen (TS) test was used to determine the size (%) of the trend. For this test, the spacing between time series data points is evenly spaced and sorted by ascending order by time.

The slope of Sen may be determined as follows:

8 $$\text{Q }= \frac{{x}_{j}-{x}_{i}}{j-i}$$

where, x_j_ and x_i_ are values at periods j and i respectively, in. The median of Sen’s slope is calculated by sorting the total N values of Q from least to largest:

9 $$Q_{med} = \{{Q}_{(N+1)/2}, \,If\, N\, is\, odd$$

10 $$ Q_{{med}}  = \left\{ {\frac{{Q_{{\left( {\frac{N}{2}} \right)}}  + Q_{{\left( {N + \frac{2}{2}} \right)}} }}{2}} \right.,If\,N\,is\,even $$

In those Equations, Q_med_ represents data trend direction and value indicates trend size. Sen’s slope is a robust assessment of trend magnitude used in hydrological time series.

### Classification based on Piper diagram

A Piper diagram is a pictorial approach developed by^56^ to aid in understanding the origins of dissolved component salts in water. This method is dependent on the idea that cations and anions are present in water in sufficient amounts to ensure the electroneutrality of the dissolved salts, and that their algebraic total of electric charges is zero. A Piper diagram depicts the chemistry of a water sample or samples graphically. Separate ternary plots depict the cations and anions. The cation plot’s apexes are calcium, magnesium and sodium cations, as well as potassium cations. To calculate the Piper diagram, initially the concentration value of every cation and anion has to be converted into relative concentration and then plotted into the ternary diagram with the help of Grapher software.

11 $$\text{Concentration (mili equivalent/litre)} =\frac{ Concentration \left(\frac{mg}{L}\right)*Valance}{Atomic \,\,weight}$$

12 $${\text{Relative Humidity }}(\%) ={\frac{Cation \,\,concentration (meq)*100\%}{Sum \,\,of \,\,all \,\,cation \,\,concentration (meq)}}$$

### Pearson’s correlation

Pearson’s correlation coefficient is calculated by dividing the covariance of the two variables by the product of their standard deviations. The definition takes the form of a “product moment,” which is the mean (the first moment around the origin) of the product of the mean-adjusted random variables; hence, the term includes the modifier product-moment. The correlation factor is detonated by r and there is a range for this r value that can determine whether the relation between two continuous variables is strong or weak. If the value of r is between 0.5 and 0.7 then it is moderate, if the value is higher than 0.7 then it is a strong correlation. So, the values nearer to zero can be regarded as no correlation between the parameters. “ + 1” indicates the parameters are proportionally increase and decrease along with time and similarly “-1” means inversely proportionate with each other.

13 $$\text{r }=\frac{\sum \left({x}_{i }- \overline{x  }\right)\left({y}_{i }- \overline{y  }\right)}{\sqrt{\sum {\left({x}_{i }- \overline{x  }\right)}^{2 }\sum {\left({y}_{i }- \overline{y  }\right)}^{2}}}$$

where, r = correlation coefficient, ${x}_{i }=values\,of\, the\,x-variable$ in a sample, $\overline{x }$= mean of the values of the x-variables, ${y}_{i}$= $values\, of\, the\, y-variable$ in a sample and $\overline{y }$= mean of the values of the y-variables.

### Gibbs diagram

The groundwater quality for drinking and irrigation purposes was assessed based on WHO (1984), standards. The Gibbs diagram is widely used to establish the relationship of water composition and aquifer lithological characteristics ^57^. Three distinct fields such as precipitation dominance, evaporation dominance and rock-water interaction dominance areas are shown in the Gibbs diagram. The predominant samples fall in the rock-water interaction dominance and few samples evaporation and precipitation dominance field of the Gibbs diagram. The rock-water interaction dominance field indicates the interaction between rock chemistry and the chemistry of the percolation waters under the subsurface. Gibbs ratios I and II for anion and cation, respectively are given below.

$$ {\text{Gibbs ratio I (for anion)  =  CI}}^{ - } {\text{/ (CI}}^{ - } {\text{  +  HCO}}_{3} ^{ - } {\text{)}}{\text{.}} $$

$$  {\text{Gibbs ratio II (for cation)  =  Na}}^{ + } {\text{/ (Na}}^{ + } {\text{  +  Ca}}^{{2 + }} {\text{)}}{\text{.}}  $$

### QGIS

Quantum GIS, commonly referred to as QGIS, stands out as a free and open-source desktop GIS software that operates seamlessly across different platforms. Offering features such as viewing, editing, and geospatial data analysis, QGIS is enhanced by numerous plug-ins that expand its functionality. Similar to other GIS tools, it is designed to capture, store, analyze, and manage data along with spatially referenced attributes tied to the Earth. Its versatility makes it particularly valuable for addressing water resource challenges, such as assessing water quality and managing water resources at local or regional levels. Many hydrologists rely on GIS technology to integrate diverse data applications into a cohesive and easily manageable system. GIS plays a crucial role in pinpointing potential sources of contamination by creating maps that display the locations of industrial facilities, agricultural zones, and various land uses that could impact the quality of groundwater. By collecting and visualizing information about subsurface water on maps, GIS facilitates a clearer understanding of areas with clean or contaminated water. Additionally, GIS enables the comparison of water quality against safety regulations, ensuring that the water is safe for human use^58^.

## Results and discussion

### Box and Whisker plot

**Figure 2:**
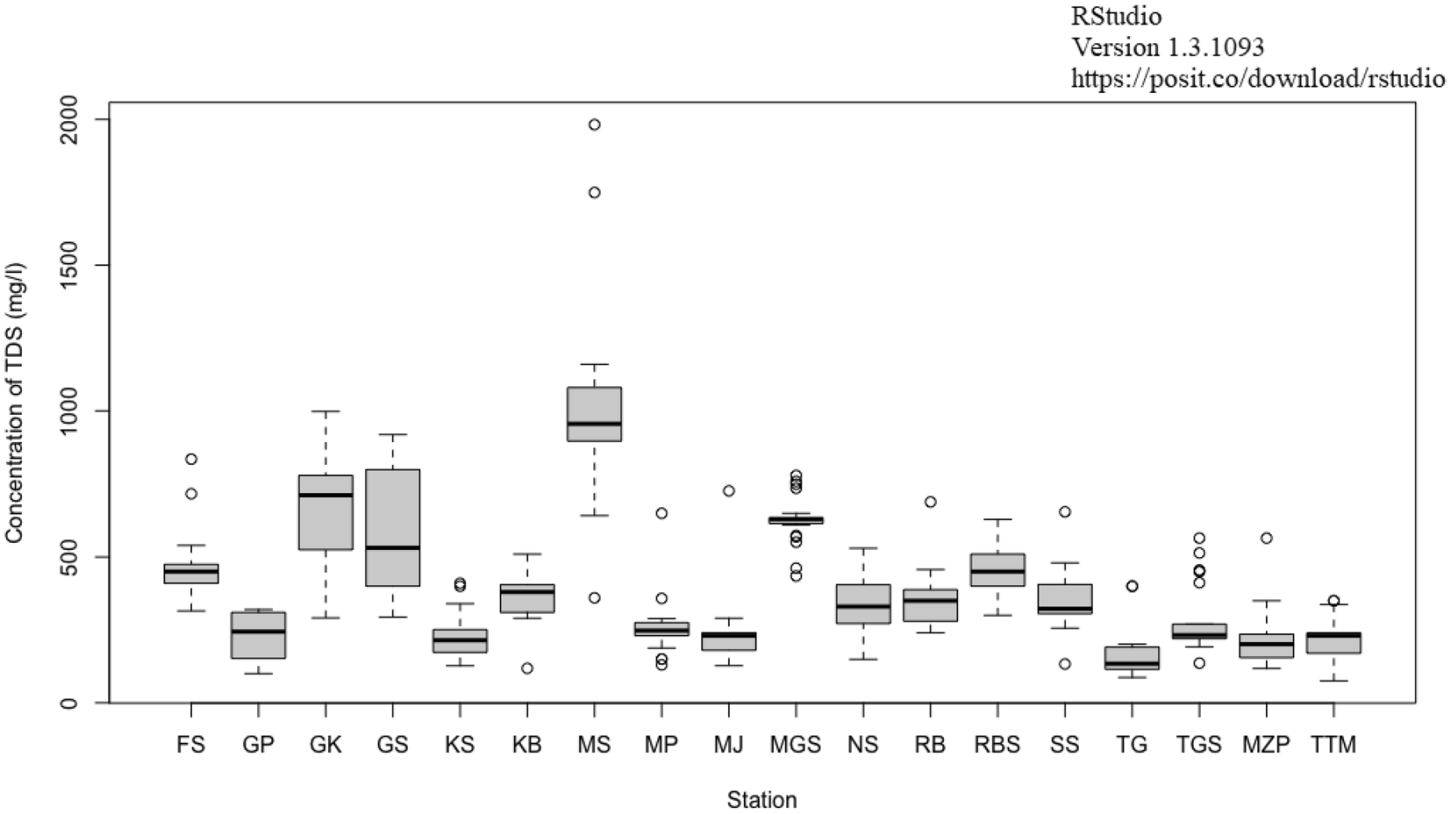
Box and Whiskers plot of groundwater quality parameters for 18 stations Whisker plots of 15 groundwater quality parameters for 18 stations (*FS* Faridpur Sadar, *GP* Gazipur Sreepur, *GK* Gopalganj Kashiani, *GS* Gopalganj Sadar, *KS* Kishoreganj Sadar, *KB* Kishoreganj Bhairab, *MS* Madaripur Sadar, *MP* Mohammadpur, *MJ* Motijheel, *MGS* Munshigani Sreenagar, *NS* Narsingdi Sadar, *RB* Rajbari, *RBS* Rajbari Sadar, *SS* Sherpur Sadar, *TG* Tangail, *TGS* Tangail, MZP-Mirzapur, *TTM* Textile Mill Tangail.

A variability study of groundwater quality parameters is critical for researchers in making decisions. The compact structure of box and whisker plots^55^ facilitates side-by-side comparisons of numerous datasets, which can be difficult to grasp using more comprehensive representations, such as the histogram^59^. These plots graphically illustrate the statistical distribution in a way that a wide variety of people can comprehend. The box and whisker plot take the following form: a centre horizontal line indicating the median and top and bottom horizontal lines representing the interquartile range as shown in Fig. 2.

The bottom and top horizontal lines in the boxes represent the 25th and 75th percentiles, respectively. Vertical lines are used to represent the outside ranges (as shown by the whiskers). If the median line is considerably pushed away from the centre, this may indicate skewness in the distribution. The length of the interquartile range (IQR), as illustrated by the box, is a measure of the relative dispersion of the centre 50% of a dataset, just as the length of each whisker is a measure of the relative dispersion of the dataset’s outer range.

### Mann–Kendall (Trend analysis)

**Figure 3:**
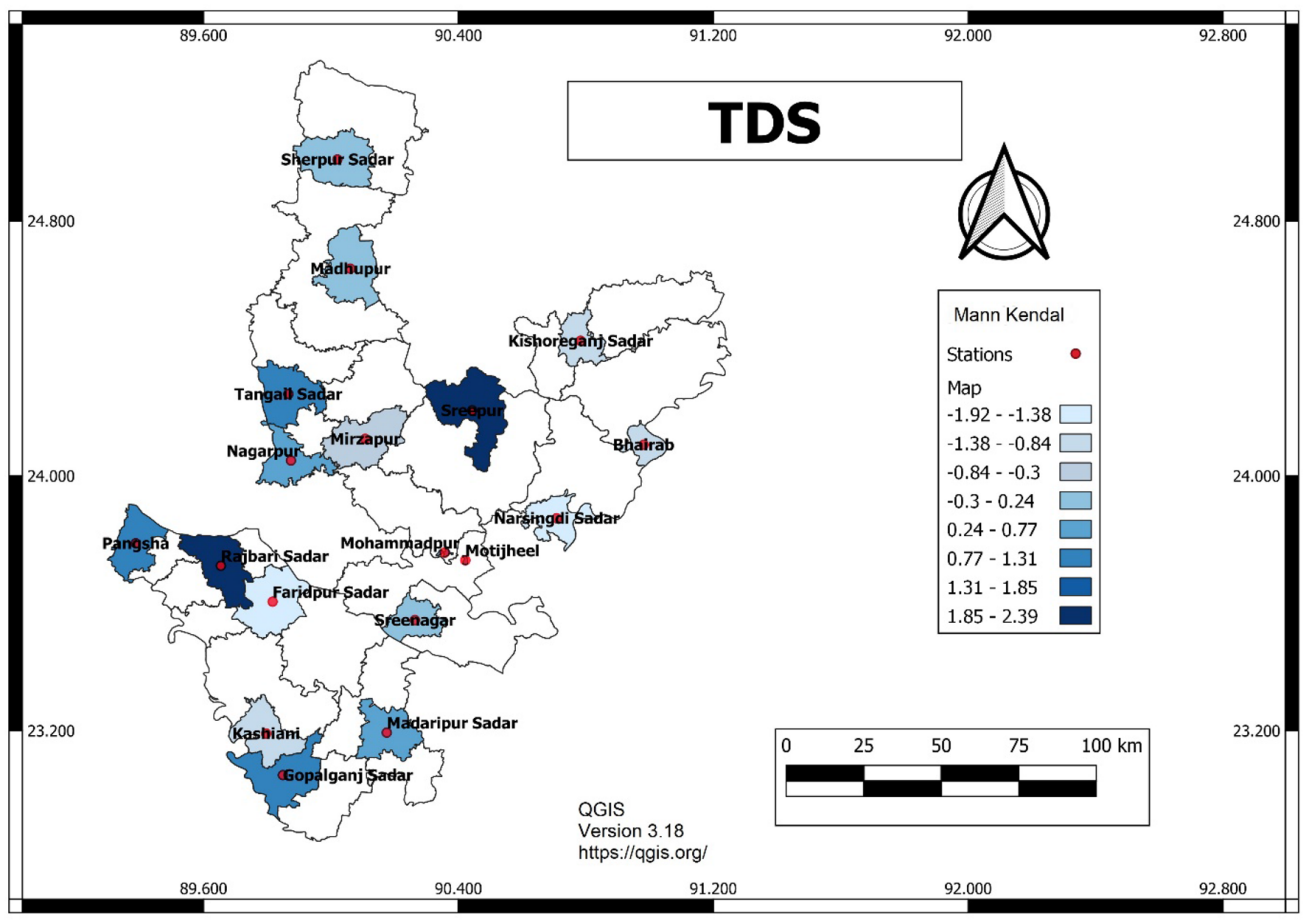
GIS mapping of Mann Kendal value for TDS (QGIS, Version 3.18, https://qgis.org/).

The Mann–Kendall test, Modified Mann–Kendall test and Sen’s slope estimator were applied to analyse 15 variables. Among these variables, the Mann–Kendall test statistic (Z) value of zero means no change, greater than zero means positive and less than zero means negative change. As shown in Fig. 3, most of the stations are in positive trends for TDS except Faridpur Sadar, Gopalganj (Kashiani), Munshiganj Sreenagar, Narsingdi Sadar and Mirzapur. A positive trend means that over time TDS concentration has increased and a negative trend means decreasing. For TDS significantly, negative trend occurs at Faridpur Sadar station and a positive trend at Rajbari Sadar. For calcium the highest decreasing trend occurs at Gopalganj Kashiani station and a positive trend at Textile Mill Tangail. For Sodium, the trend has fallen drastically at Bhairab station and increased at Tangail Textile Mill. Similarly, in Tangail Sadar, potassium, nitrate and silica have shown the highest positive trend, which is very significant.

On the other hand, chloride and sulphate have shown unusual increments in Tangail Textile Mill. Besides, Rajbari Sadar has found a highly increasing trend in carbonate and carbon dioxide. This could be the worst-case scenario if the trend is increasing at this rate. The highest positive trend rate has been pinned point down in Tangail and Rajbari. From MK tests, approximately 77% of the stations showed increasing trends in Carbonate, Bi-Carbonate, Silica, Carbon dioxide, Chloride, Fluoride, Calcium among which Carbonate, Bi-Carbonate showed significant increasing trends at 95% confidence level (CL). Almost all of the stations show positive trend in carbonate (ranging from 2.5 ~ 0.5) and bi carbonate (ranging from 2.51 ~ 0.15) which is a major concern for the region. The reason behind this may be identified as geology is important in the dynamics of groundwater levels in this region. Furthermore, groundwater levels have a substantial impact on groundwater quality.

### Sen’s slope

**Figure 4:**
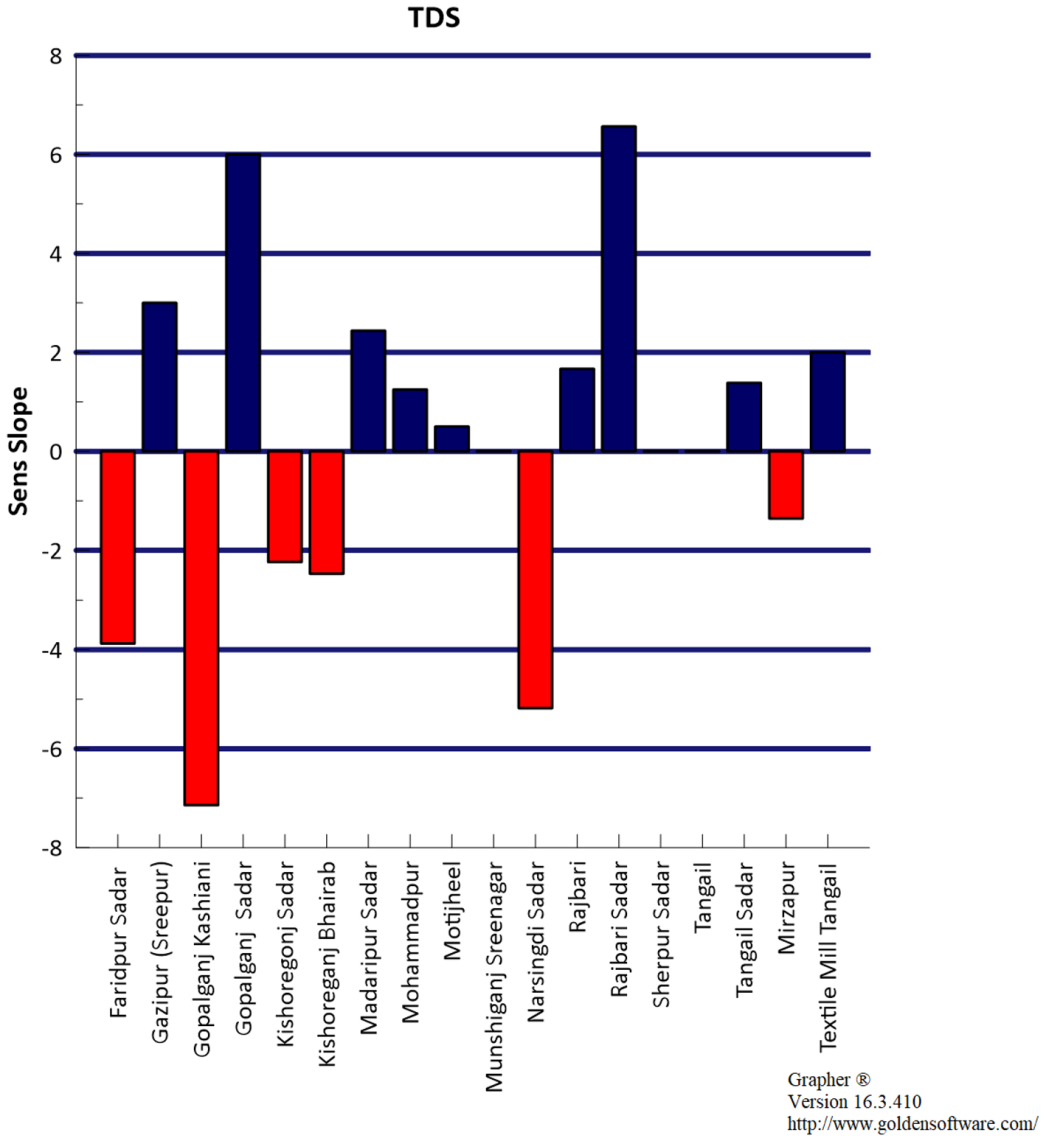
Bar diagram of Sen’s slope for TDS.

**Figure 5:**
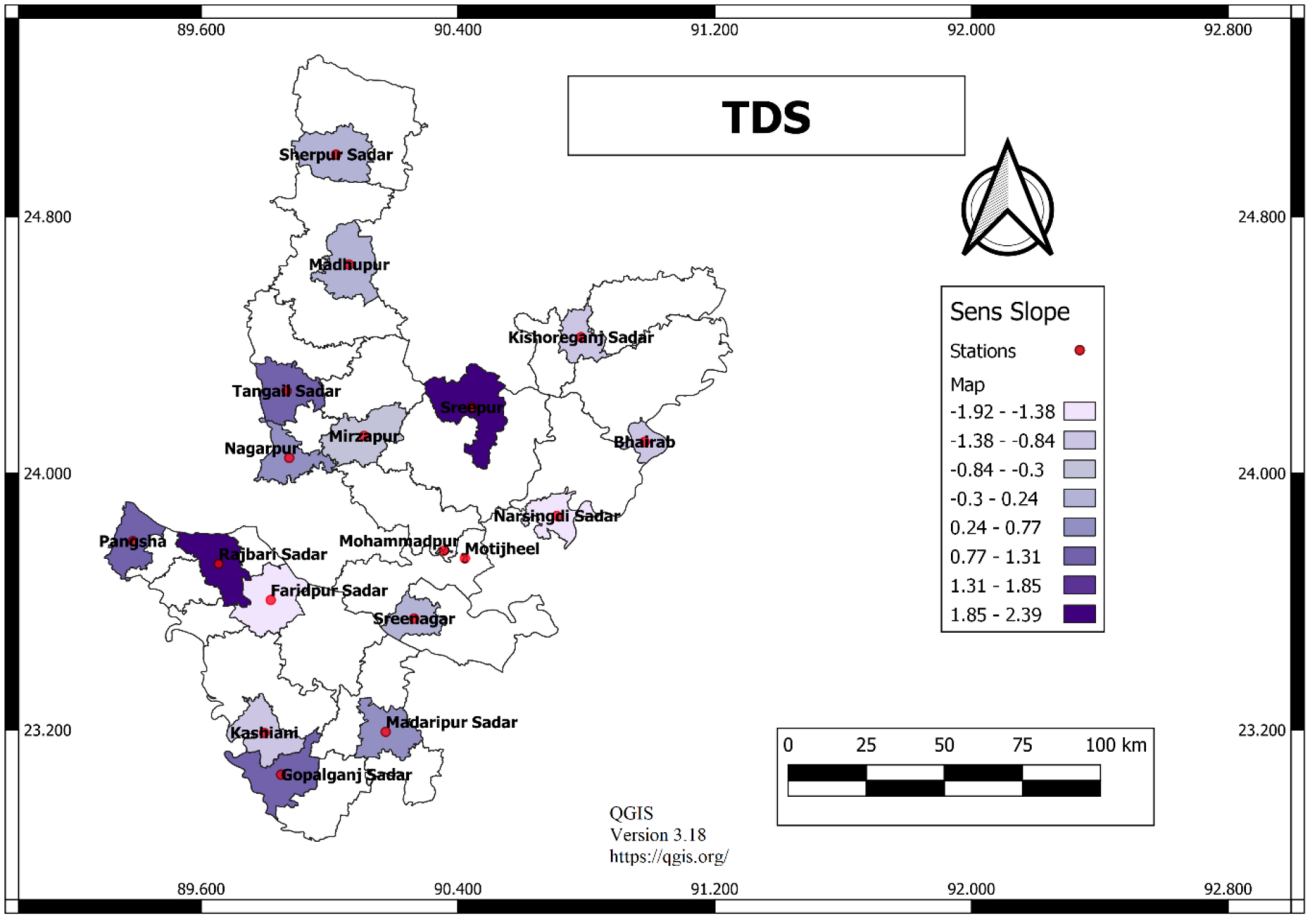
GIS mapping of Sen’s slope value for TDS (QGIS, Version 3.18, https://qgis.org/).

Sen’s slope test was applied in this study to determine the sloping characteristic like positive or negative trends as shown in Fig. 4. A positive slope indicates that over time concentration increases and a negative trend indicates over time concentration decreases. Zero slope means no change occurs over time. Figure 5 presents the GIS mapping of Sen’s slope value for TDS. It shows the variation of Sen’s slope value for TDS in different stations.

### Major chemical composition of groundwater (Piper diagram)

**Figure 6:**
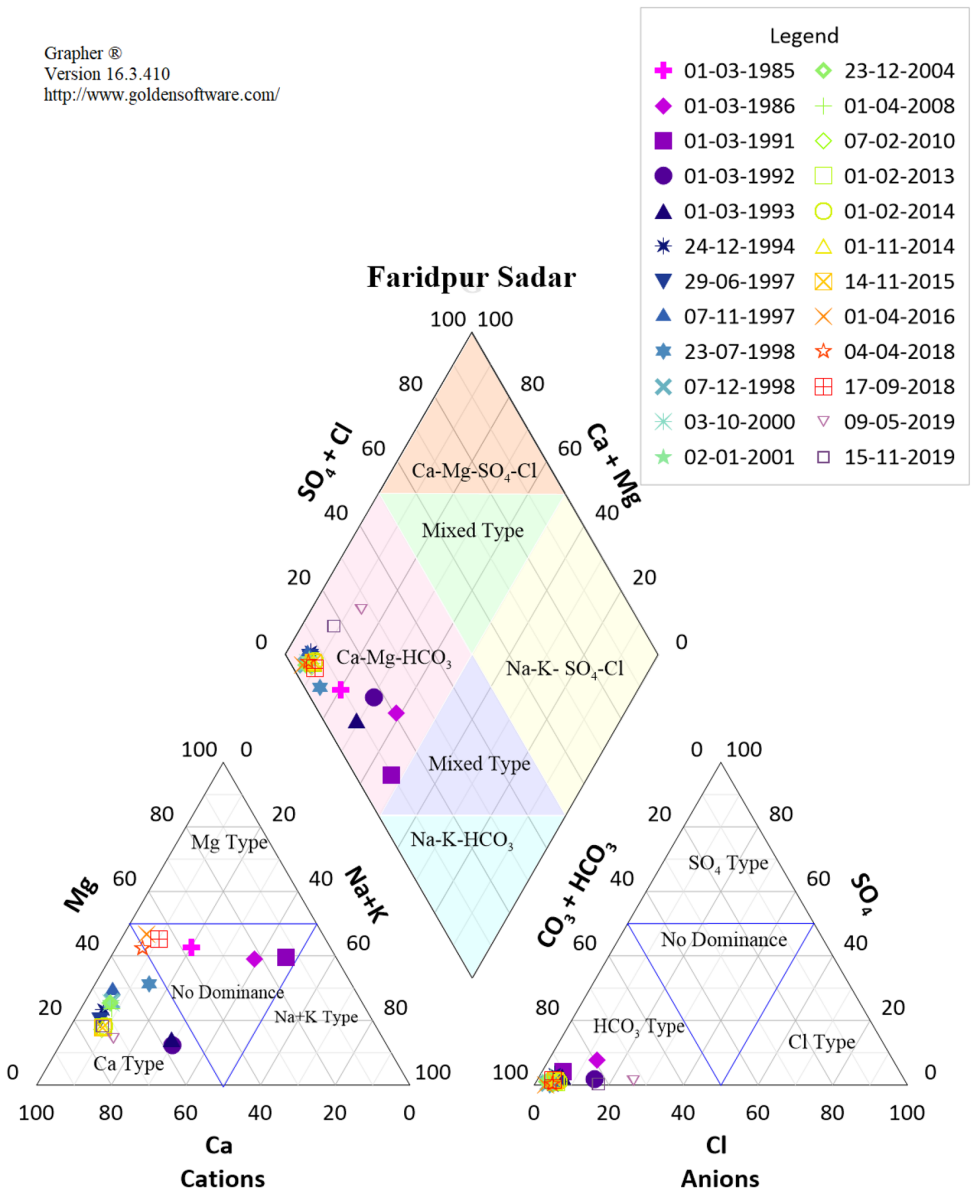
Piper diagram for Faridpur Sadar.

The Piper diagram represents the classification of the water according to hydrochemical facies and different kinds of prominent ions^56^. The relative concentration of groundwater quality is shown in Fig. 6 and it identifies the primary characterization of water.

Piper Diagram detected that the majority of the groundwater samples of every station except Madaripur and Tangail Mirzapur were centred in the diamond’s left corner indicating that the groundwaters in the examined region were predominantly rich in Ca^2+^, Mg^2+^ and HCO_3_^−^. In Madaripur Sadar, Na^+^ + K^+^ and Cl^−^ have the most influence in the middle of the periodic timeline. It identifies that the Madaripur Sadar had slightly salt constituents in the water composition earlier but now it is in mixed condition. All other 17 stations have Alkali type water which implies that the hardness is present in the samples.

This study shows that the most dominant anion is HCO_3_^−^ which is predominantly high in every station. The water contains (HCO_3_^−^) equivalent fractions among total anions of up to 0.99 from a lower value of 0.80 on average for every individual station. In Munshiganj and Madaripur Sadar, the anion changed from HCO_3_^−^ to Cl^−^. It is noticed that the Madaripur Sadar has the highest domination of Cl^−^ anions which is almost 80% of total dissolved anions. In the case of Tangail Sadar the water quality changed to no dominance zone from high alkaline HCO_3_^−^. On the other hand, Tangail (Mirzapur) contains opposite hydrochemical facies like it becomes alkaline from no dominance zone. There are numerous changes in groundwater quality along with time series regarding cations. Faridpur Sadar, Tangail Sadar, Rajbari Sadar, Gopalganj Sadar and Kishoregonj Sadar have the same transition of chemical composition in groundwater samples. In Motijheel and Mohammadpur, the water ion is in Ca^2+^ and HCO_3_^-^ dominant type which was also found in a previous study, which concluded that the sample relatively neutral to slightly alkaline pH and moderate to high concentrations of calcium and bicarbonate ions^60^. Table 1 shows the hydrochemical facies and different kinds of prominent ions of the stations.

**Table 1:** Summary of Piper diagram.

Station Name	Cations	Anions	Diamond
Faridpur Sadar	NDZ, Ca^2+^	HCO_3_^−^ (High)	Ca^2+^–Mg^2+^–HCO_3_^−^
Gazipur (Sreepur)	Na^+^ + K^+^, Mg^2+^, NDZ, Ca^2+^	HCO_3_^−^ (High)	Mix, Ca^2+^–Mg^2+^–HCO_3_^−^
Gopalganj Kashiani	Na^+^ + K^+^, Ca^2+^	HCO_3_^−^ (High)	Ca^2+^–Mg^2+^–HCO_3_^−^
Gopalganj Sadar	NDZ, Ca^2+^	HCO_3_^−^ (High)	Ca^2+^–Mg^2+^–HCO_3_^−^
Kishoregonj Sadar	NDZ, Ca^2+^	HCO_3_^−^ (High)	Ca^2+^–Mg^2+^–HCO_3_^−^
Kishoreganj Bhairab	NDZ, Na^+^ + K^+^, Mg^2+^, Ca^2+^	HCO_3_^−^ (High)	Ca^2+^–Mg^2+^–HCO_3_^−^
Madaripur Sadar	Na^+^ + K^+^	HCO_3_^−^, Cl^-^	Mix, Na^+^–K^+^–SO_4_^2−^
Mohammadpur	Na^+^ + K^+^, Mg^2+^, Ca^2+^	HCO_3_^−^ (High)	Mix, Ca^2+^–Mg^2+^–HCO_3_^−^
Motijheel	Ca^2+^, NDZ, Mg^2+^, Ca^2+^	HCO_3_^−^ (High)	Ca^2+^–Mg^2+^–HCO_3_^−^
Munshiganj Sreenagar	Na^+^ + K^+^, NDZ, Mg^2+^, Ca^2+^	HCO_3_^−^ (High), Cl^-^	Ca^2+^–Mg^2+^–HCO_3_^−^
Narsingdi Sadar	Na^+^ + K^+^, NDZ, Ca^2+^	HCO_3_^−^ (High)	Mix, Ca^2+^- Mg^2+^–HCO_3_^-^
Rajbari	NDZ, Mg^2+^, Ca^2+^	HCO_3_^−^ (High)	Ca^2+^–Mg^2+^–HCO_3_^−^
Rajbari Sadar	NDZ, Ca^2+^	HCO_3_^−^ (High)	Ca^2+^–Mg^2+^–HCO_3_^−^
Sherpur Sadar	NDZ, Ca^2+^	HCO_3_^−^ (High)	Ca^2+^–Mg^2+^–HCO_3_^−^
Tangail	NDZ, Mg^2+^, Ca^2+^	HCO_3_^−^ (High)	Ca^2+^–Mg^2+^–HCO_3_^−^
Tangail Sadar	NDZ, Ca^2+^	HCO_3_^−^ (High), No	Ca^2+^–Mg^2+^–HCO_3_^−^
Mirzapur	Na^+^ + K^+^, NDZ, Mg^2+^, Ca^2+^	No, HCO_3_^−^ (High)	Na^+^ + K^+^,Ca^2+^–Mg^2+^–HCO_3_^−^
Textile Mill Tangail	Ca^2+^, NDZ, Ca^2+^	HCO_3_^−^ (High)	Ca^2+^–Mg^2+^–HCO_3_^−^

*NDZ* No dominance zone.

Initially, the samples were in no dominance zone but along with time change, it became Calcium type cations with a range of 60–80%. The other five samples collected from Motijheel, Sherpur Sadar, Tangail (Madhapur), Textile Mill Tangail, Rajbari (Pangsha) have determined the same kind of transformation in hydro chemical facies. The chemical compositions have changed between Ca^2+^, Mg^2+^ and No dominance zone. The rest of the samples are in different dominant zones according to periodic time changes. The highest milli equivalent concentration of Mg^2+^ is observed in Motijheel, above 80% of total cations and the highest Ca^2+^ is observed in Rajbari (Pangsha) (90%).

In addition, it is seen that all the water samples are influenced by Ca^2+^ cation in recent times except Madaripur Sadar and Sherpur Sadar. For Madaripur Sadar, the water constituents become Na^+^ + K^+^ (90%) types of Cations; for Sherpur, the samples came to fall in no dominance zone. Water hardness is primarily induced by the presence of cations such as calcium and magnesium, as well as anions such as bicarbonate, carbonate, sulphate and chloride in the water. The changes are shown in Table 1 to visualise a clear understanding of the groundwater chemistry in the study areas. Overall, it is identified that the facies have hardness issues.

### Gibbs diagram

**Figure 7:**
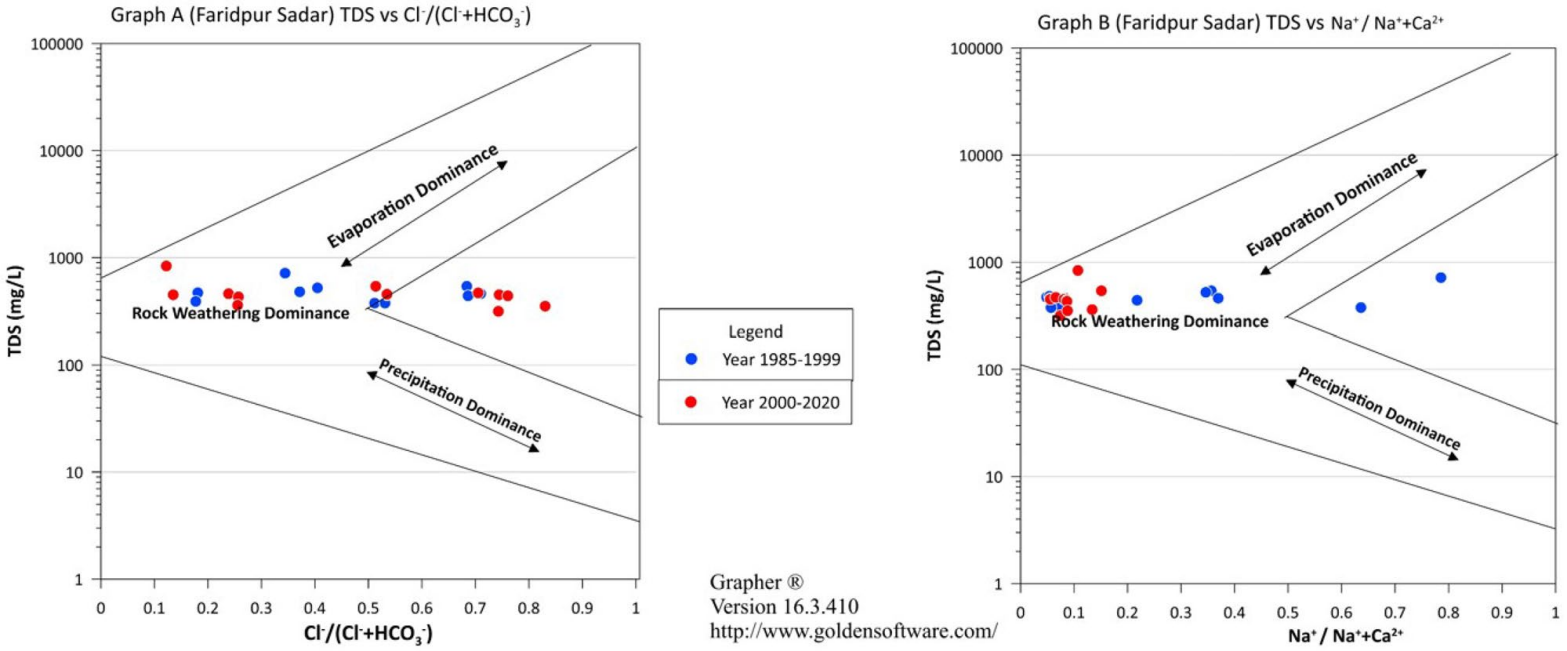
Gibbs diagram for Faridpur Sadar.

The majority of the samples, as shown in Fig. 7, are found in the rock weathering dominating region, suggesting that groundwater chemistry is characterized by elevated concentrations of ions derived from the dissolution of minerals such as silicates, carbonates, and sulfates. Common cations associated with rock weathering include calcium (Ca2 +), magnesium (Mg2 +), with the maximum density found in the range of 0.1 ~ 0.5 except Madaripur Sadar, Munshiganj Sreenagar and Narsingdi Sadar. These 3 stations have shown their cations maximum density range more than 0.5. Besides, only Madaripur Sadar ions have influenced by the evaporation zone which indicates the salinity issues in the last few decades for this region.

But for the anions Cl^-^/(Cl^-^ + HCO_3_^-^) , there are 50% of the stations have shown a maximum density limit of more than 0.5. It is identified that all the stations have hardness issues which can be severe conditions by the time in near future. The diagram also denoted that after the year 2000, all of the stations experienced the same pattern change in terms of cation exchange. Their range is between 0 ~ 0.4 except the Madaripur and Munshiganj. These two stations have crossed the limit 0.5 in the recent decades. Another study earlier shows that, In Dhaka city majority of the ions are largely influenced by rock-water interaction and precipitation, as indicated by the Gibbs plot analysis The dominant hydrochemical facies in the groundwater are Ca^2+^-Mg^2+^-HCO_3_^-^ followed by other types like Ca^2+^–Mg^2+^–Cl^-^–SO_4_^2-^, Na^+^–K^+^–Cl^−^–SO_4_^2−^, and Na^+^–K^+^–HCO_3_^−^^43^. Table 2 shows the noticeable changes in Gibbs diagram result.

**Table 2:** Summary of the Gibbs diagram.

Station Name	Dominant Zone	Range: Cl-/(Cl^-^ + HCO_3_^-^)	Maximum Density Region: Cl^-^/(Cl^-^ + HCO_3_^-^)	Range: Na^+^/(Na^+^ + Ca^2+^)	Maximum Density Region: Na^+^/(Na^+^ + Ca^2+^)
Faridpur Sadar	Rock Weathering	0.15 ~ 0.85	0.7 ~ 0.9	0.1 ~ 0.8	0.1 ~ 0.2
Gazipur (Sreepur)	Rock Weathering	0.4 ~ 0.85	0.4 ~ 0.6	0 ~ 0.75	0 ~ 0.2
Gopalganj Kashiani	Rock Weathering	0.35 ~ 0.9	0.7 ~ 0.9	0 ~ 0.45	0 ~ 0.2
Gopalganj Sadar	Rock Weathering	0.25 ~ 0.9	0.25 ~ 0.5	0 ~ 0.75	0 ~ 0.4
Kishoregonj Sadar	Rock Weathering	0.2 ~ 0.9	0.2 ~ 0.4	0 ~ 0.8	0 ~ 0.1
Kishoreganj Bhairab	Rock Weathering	0.05 ~ 0.95	0.65 ~ 0.85	0 ~ 0.9	0 ~ 0.3
Madaripur Sadar	Evaporation	0.55 ~ 1	0.85 ~ 1	0.55 ~ .95	0.6 ~ 0.8
Mohammadpur	Rock Weathering	0 ~ 0.9	0 ~ 0.3	0 ~ 0.8	0 ~ 0.3
Motijheel	Rock Weathering	0.05 ~ 0.95	0.6 ~ 0.8	0.05 ~ 0.6	0.05 ~ 0.7
Munshiganj Sreenagar	Rock Weathering	0.25 ~ 1	0.5 ~ 0.75	0.1 ~ 0.95	0.5 ~ 0.75
Narsingdi Sadar	Rock Weathering	0.1 ~ 0.9	0.45 ~ 0.65	0.15 ~ 0.9	0.5 ~ 0.7
Rajbari Pangsha	Rock Weathering	0.05 ~ 0.85	0.45 ~ 0.7	0 ~ 0.55	0 ~ 0.2
Rajbari Sadar	Rock Weathering	0.2 ~ 1	0.3 ~ 0.5	0 ~ 0.95	0 ~ 0.2
Sherpur Sadar	Rock Weathering	0.15 ~ 1	0.8 ~ 1	0.15 ~ 0.85	0.2 ~ 0.4
Tangail (Madhupur)	Rock Weathering	0.05 ~ 0.9	0.05 ~ 0.25	0 ~ 0.65	0.2 ~ 0.35
Tangail Sadar	Rock Weathering	0 ~ 1	0.3 ~ 0.55	0 ~ 0.5	0 ~ 0.25
Tangail Mirzapur	Rock Weathering	0 ~ 0.8	0 ~ 0.2	0 ~ 0.95	0 ~ 0.3
Textile Mill Tangail	Rock Weathering	0 ~ 0.8	0 ~ 0.2	0 ~ 0.5	0 ~ 0.15

### Correlation of the parameters

**Figure 8:**
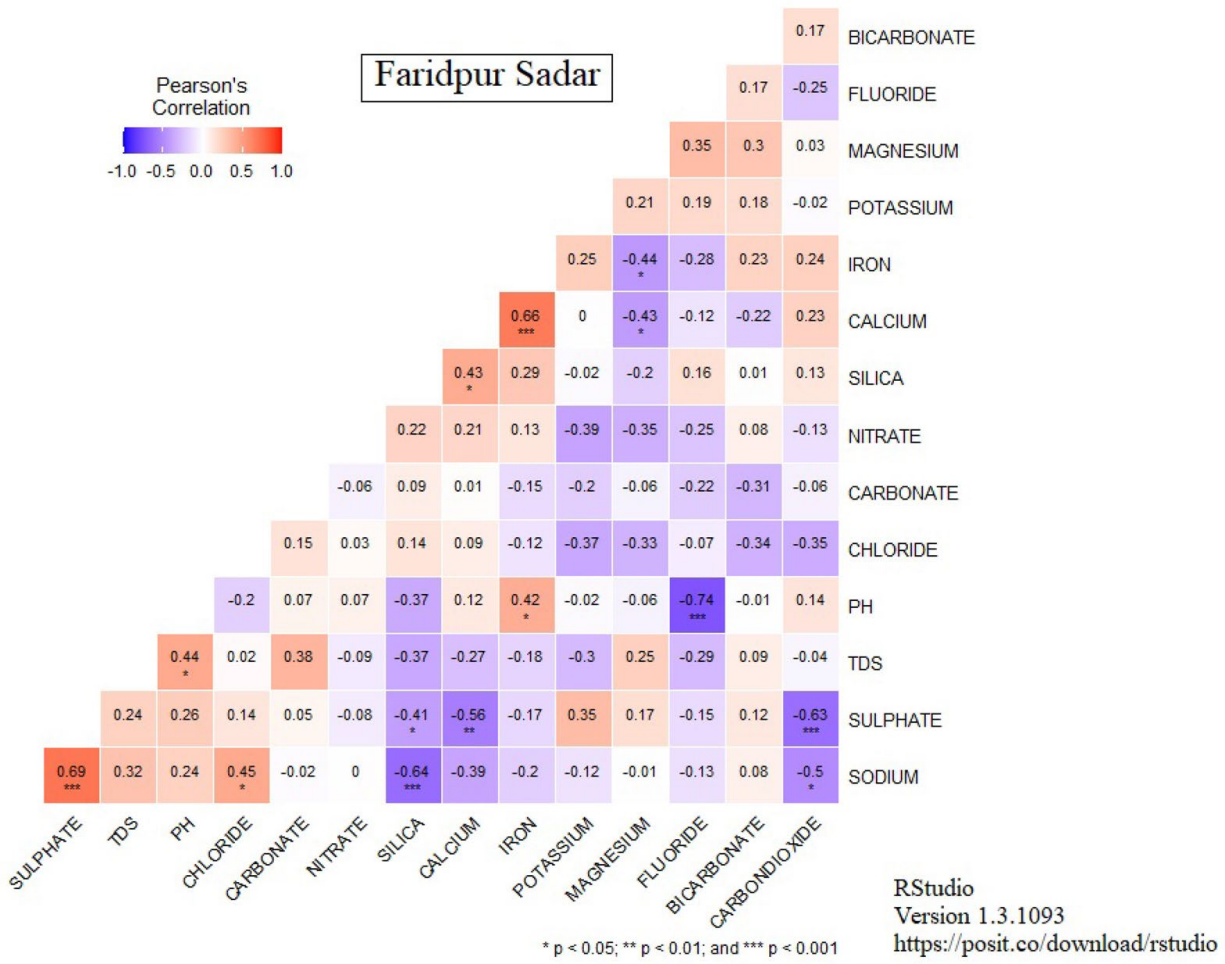
Pearson’s correlation matrix for Faridpur Sadar.

Pearson’s matrix has explored strong correlations, indicating that those parameters have a close association with each other. Figure 8 and Table. 3 show that there is a significant correlation between Na^+^ and SO_4_^2−^ in Faridpur Sadar (0.69), Mohammadpur (0.92), Narsingdi Sadar (0.72), Rajbari Pangsha (0.7) and Tangail Sadar (0.69). On the other hand, Mg^2+^ and SO_4_^2-^ have a notable co-relation that is observed in Madaripur Sadar (0.82).

**Table 3:** Summary of Pearson correlation matrix.

No	Station Name	High positive correlation	High negative correlation	No relation
1	Faridpur Sadar	Na^+^ and SO_4_^2−^ (0.69)	pH and F^-^ (0.74)	Ca^2+^ and K^+^, Na^+^ and NO_3_^-^
2	Gazipur (Sreepur)	Cl^-^ and CO_3_^2−^ (0.65)	Na^+^ and Si^2+^ (0.75)	SO_4_^2-^ and K^+^
3	Gopalganj Kashiani	Ca^2+^ and Fe^2+^ (0.65), TDS and HCO_3_^-^ (0.65)	-	-
4	Gopalganj Sadar	TDS and HCO_3_^−^ (0.75), Ca^2+^ and CO_3_^2-^ (0.85)	Ca^2+^ and Na^+^ (0.65)	CO_3_^2-^ and pH, CO_3_^2-^ and NO_3_^-^
5	Kishoregonj Sadar	Na^+^ and CO_3_^2−^ (0.74)	Ca^2+^ and Na^+^ (0.66)	TDS and CO_3_^2-^
6	Kishoreganj Bhairab	Na^+^ and Cl^−^ (0.77)	Ca^2+^ and Na^+^ (0.75)	K^+^ and F^-^ , pH and SO_4_^2-^
7	Madaripur Sadar	Mg^2+^ and SO_4_^2−^(0.82)		SO_4_^2-^ and pH
8	Mohammadpur	Na and SO_4_^2−^ (0.92), TDS and F^-^ (0.82), TDS and HCO_3_^−^ (0.69), HCO_3_^−^ and CO_3_^2−^ (0.69)	Na^+^ and CO_3_^2-^(0.65)	TDS and Na^+^
9	Motijheel	Cl^−^ and SO_4_^2−^ (0.77)		HCO_3_^-^ and NO_3_^-^, F^-^ and Na^+^
10	Munshiganj Sreenagar	Na^+^ and Cl^-^ (0.66)	Mg^2+^ and Si^2+^ (0.8)	CO_3_^2-^ and Cl^-^
11	Narsingdi Sadar	Na^+^ and Cl^-^ (0.77), SO_4_^2-^ and Na^+^ (0.72)	CO_3_^2-^ and SO_4_^2-^(0.67), Si^2+^ and Mg^2+^(0.66)	Na^+^ and Fe^2+^, TDS and Mg^2+^
12	Rajbari	TDS and Cl^-^ (0.76), Ca^2+^ and CO_3_^2-^ (O.76),SO_4_^2-^ and Cl^-^ (0.74), Na^+^ and SO_4_^2-^ (0.7)	-	TDS and Si^2+^ , TDS and NO_3_^-^
13	Rajbari Sadar	-	-	HCO_3_^-^ and K^+^
14	Sherpur Sadar	Na^+^ and Cl^-^ (0.88), TDS and Cl^-^ (0.77)	F^-^ and Ca^2+^ (0.67)	Ca^2+^ and pH, Fe^2+^ and CO_3_^2-^ , Mg^2+^ and K^+^
15	Tangail	HCO_3_^-^ and Si^2+^ (0.83)	CO_3_^2-^ and Na^+^ (0.74)	Na^+^ and Mg^2+^ , Si^2+^ and Cl, Na^+^ and K^+^
16	Tangail Sadar	Si^2+^ and CO_3_^2-^ (0.68), Na^+^ and SO_4_^2-^ (0.69)	Cl^-^ and Fe^2+^ (0.69)	K^+^ and F^-^
17	Mirzapur	HCO_3_^-^ and Na^+^ (0.72), HCO_3_^-^ and TDS (0.72),TDS and Na^+^ (0.73)	Ca^2+^ and Cl^-^ (0.77)	-
18	Textile Mill Tangail	Cl^-^ and SO_4_^2−-^ (0.88)	NO_3_^-^and pH (0.67)	Mg^2+^ and Fe^2+^

These relations conclude that the stations have a high hardness possibly bring critical complexities in a chemical reaction inside groundwater. The reason is that gypsum and other common minerals leach into the water, producing sulphate as a by-product. Its concentration is more likely to rise with domestic and industrial sewage. This is mostly dependent on the depth of the water when it comes into touch with the aquifer under the rock surface [65]. In a previous study, it is found that in Dhaka division near Savar, SO_4_ showed a strong negative correlation with dissolved oxygen and HCO_3_^-^, while HCO_3_^-^ showed a strong negative correlation with DO and a positive correlation with SO_4_^2−^ [66].

Besides, Kishorganj Sadar and Tangail Mirzapur have an increasing correlation of temporary hardness between Na^+^ and CO_3_^2−^ (0.74) and HCO_3_^-^ and Na^+^ (0.72) respectively. Another severe problem observed in Kishorganj Bhairab (0.77), Munshiganj, Sreenagar (0.66), Narsingdi Sadar (0.77) and Sherpur Sadar (0.88) is a strong positive correlation between Na^+^ and Cl^-^. In this case, if Na^+^ and Cl^-^ increase simultaneously then the salinity of the groundwater will be increased, thus implying the effect on both the agriculture field and human health.

The majority of the water samples revealed detectable levels of chlorine. When combined with Ca^2+^ and Mg^2+^, large quantities of Cl^-^ increase corrosiveness of water. TDS has a high association with sodium, showing the relevance of halite in the aquifer’s overall ionic composition. When TDS levels in groundwater are over this threshold, it causes undesirable taste and gastrointestinal discomfort as reported [67].

Some parameters show highly negative values, determining that these variables have an inverse relation. The highly negative correlations are shown in Table 3. Tables A1–A19 related to groundwater quality in various locations are supplied as supplementary materials. In addition, FiguresA1–A3 are provided as Supplementary Materials related to Pearson’s correlation matrix, GIS mapping of Man Kendal value and Piper diagram using the selected water quality parameters in various locations, respectively.

### Practical implication

The analysis of groundwater trends using non-parametric methods can provide valuable insights and has been widely used by researchers in different parts of the world for trend analysis of groundwater quality [68]. By identifying pollution sources and trends, the research supports to improve water treatment and pollution control strategies, aiming for better water quality in the future. It provides a basis for regular monitoring and management of groundwater quality to ensure it remains suitable for long-term agricultural use, thus supporting food security in the region. The findings can guide local authorities and farmers in selecting appropriate water sources for irrigation, drinking, and industrial purposes, based on the levels of salinity, and alkalinity. By highlighting the correlation between different water quality indicators, the study offers insights into managing groundwater resources more effectively, ensuring sustainable agricultural practices. By analyzing the parameters, the research provides a basis for designing effective landfill management and leachate treatment systems to prevent further groundwater contamination. This involves developing strategies for better pollutant control and taking preventive measures by local governing bodies to ensure the safety of groundwater resources. The findings can guide future research on how to improve water quality in areas where groundwater does not meet the necessary standards for drinking and irrigation.

## Conclusions

Man Kendal and Sens slope tests revealed that the groundwater hardness exceeds the allowable limit for the majority of aquifer system locations. Some stations have already crossed the limits of water quality standards instead it shows a positive trend in the Man Kendal and Modified Man Kendal Test as well. The chemical composition of groundwater demonstrated that numerous processes, such as salinization, water hardness and alkalinity, regulate water quality. From the Piper diagram, almost all stations have shown Mg^2+^ –HCO_3_^−^ type characteristics except Madaripur Sadar. Madaripur Sadar had a high probability of increasing Na^+^ and Cl^-^ ions earlier. The Pearson Correlation coefficient also confirms the increments of this salt. Besides, Mg^2+^ and SO_4_^2−^ strongly correlate in almost every station, indicating the sample has total hardness.

Furthermore, it is worth noting that the primary justification for doing groundwater-quality research in the region field data is often readily unavailable and inadequate. One of the limitations of this study is that thedata is not properly consistent and the other one is to continue this study on a larger magnitude, more data should be needed for the study area. Such locations, predicted to be more sensitive to groundwater pollution and exploitation, urgently require scientific research to ensure the long-term management of the regional groundwater resources. The study findings will be valuable to policymakers and decision-makers in developing effective groundwater consumption and making prediction analysis for Pliocene aquifer systems in other regions of the world to ensure safe and good quality of groundwater. The study suggests that while the groundwater in the area can be safely used for long-term irrigation, some samples may not be suitable for industrial uses due to high alkali concentrations. Future research should focus on understanding the factors contributing to these changes and how they can be mitigated. It is recommended to monitor the groundwater quality regularly, especially focusing on iron concentration, TDS, and pH values, to ensure its suitability for various uses. Further research could explore ways to treat the water, making it safe for drinking and industrial purposes, especially considering the high correlation between SSP and SAR, which indicates a specific area of water chemistry that could be targeted for improvement.

### Supplementary Information

Supplementary Information 1.

Supplementary Information 2.

## Data Availability

Further information, data and figures are supplied as Supplementary Materials.
