# Supplementary material for: GIS based geostatistical modelling and trends analysis of groundwater quality for suitable uses in Dhaka division
Source: Sci Rep. 2024 Jul 29;14:17449. doi: 10.1038/s41598-024-66567-z (PMC11286770; doi:10.1038/s41598-024-66567-z)
Supplement: Supplementary file 1 — Supplementary Information 1. [file 41598_2024_66567_MOESM1_ESM.docx]

Appendix A1


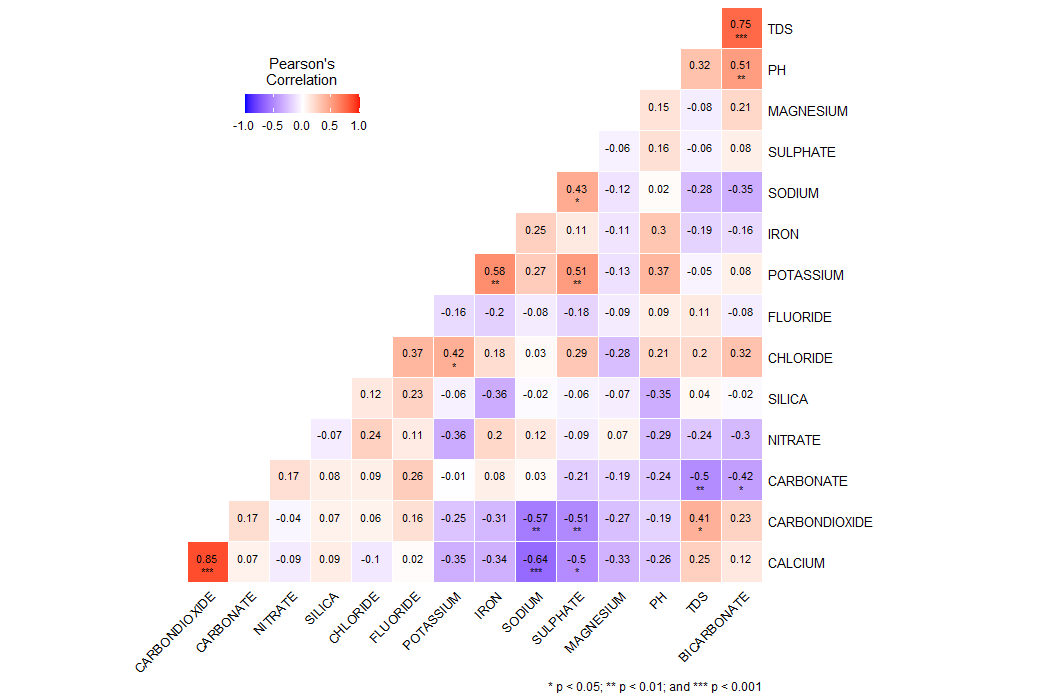

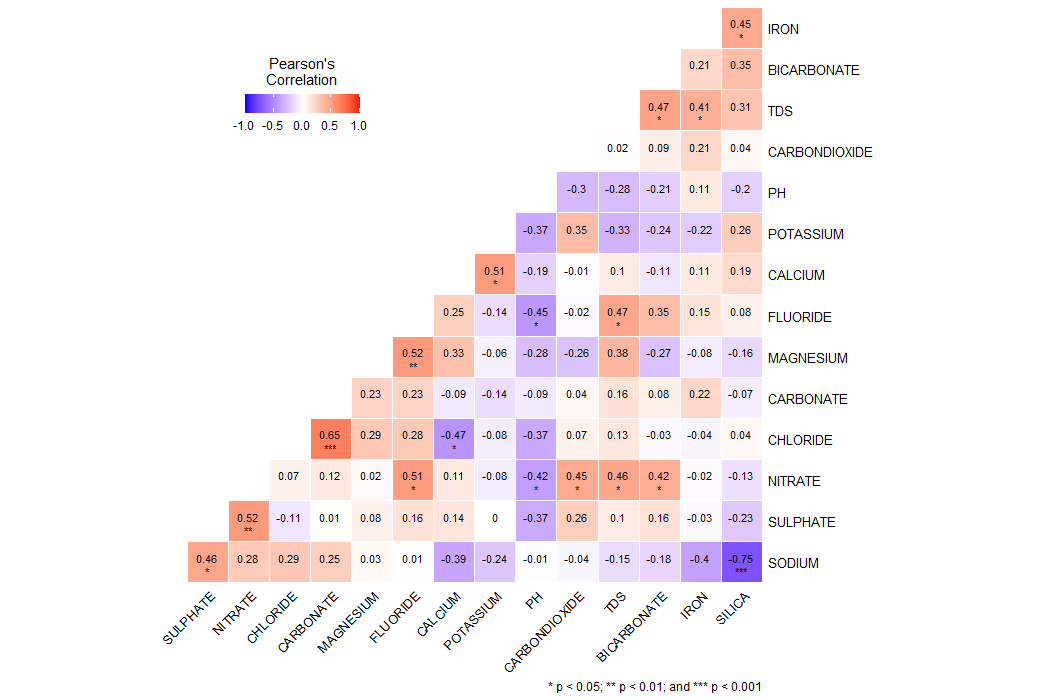


(2) Gopalganj Sadar

(1) Gazipur


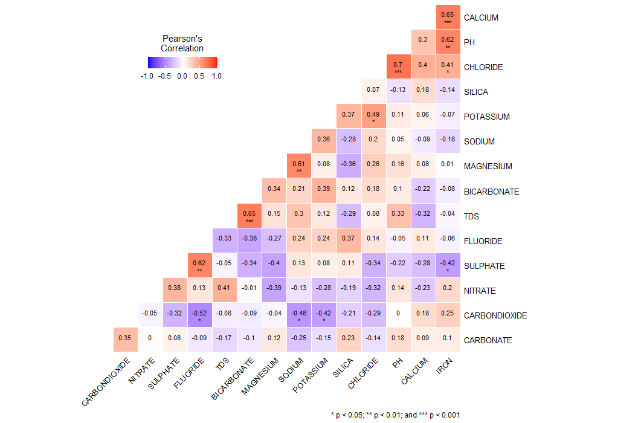


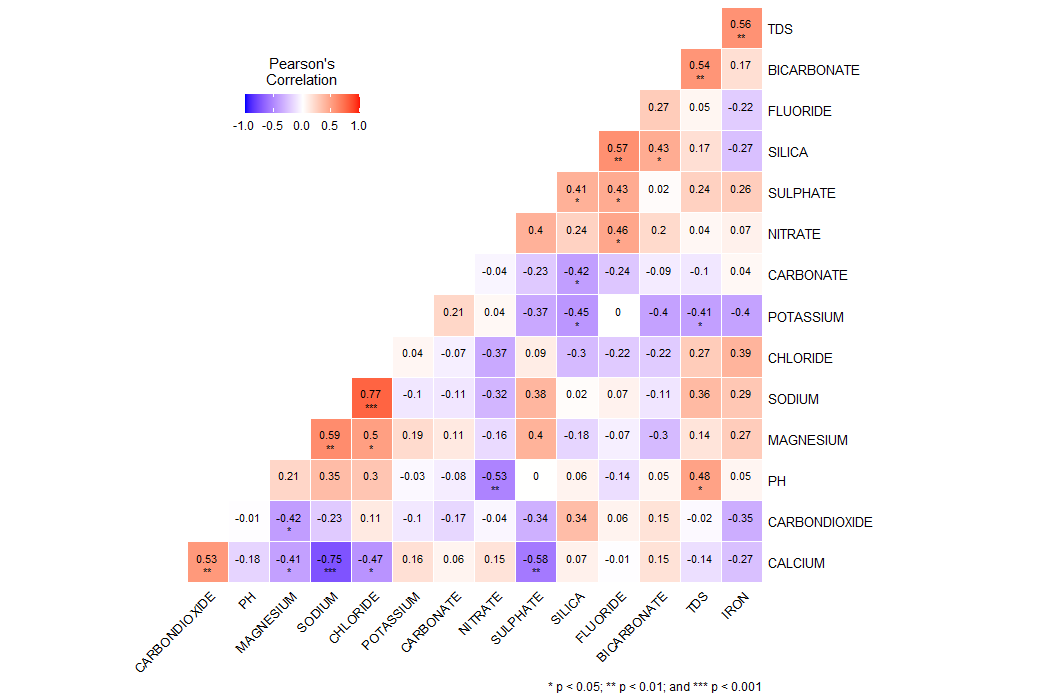


(4) Kishoreganj Bhairab

(3) Gopalganj Kashiani


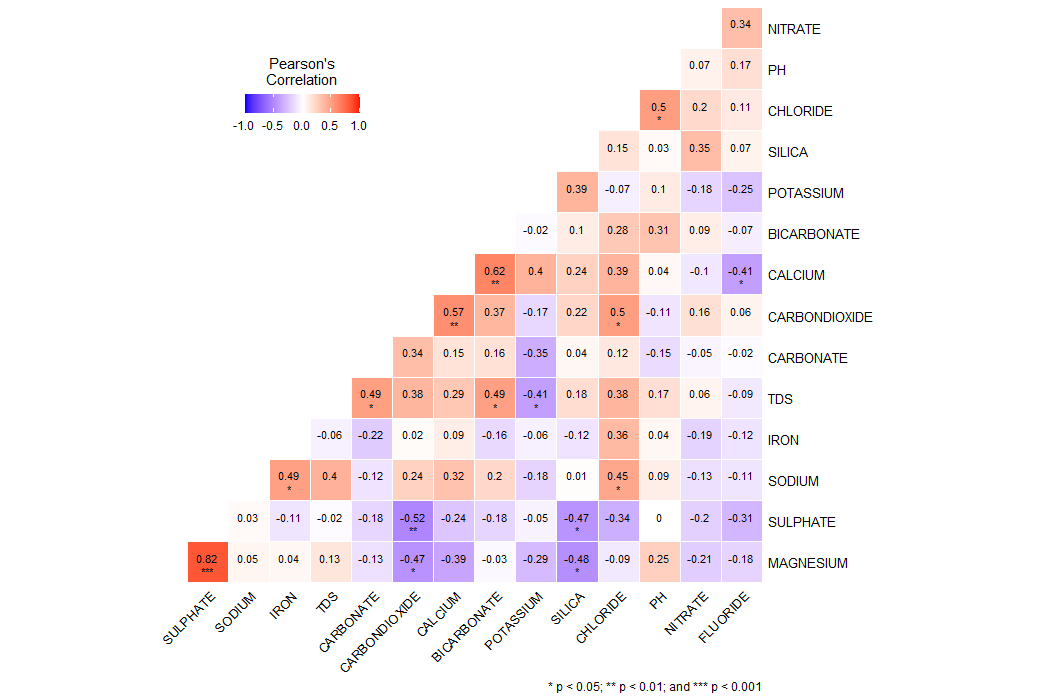

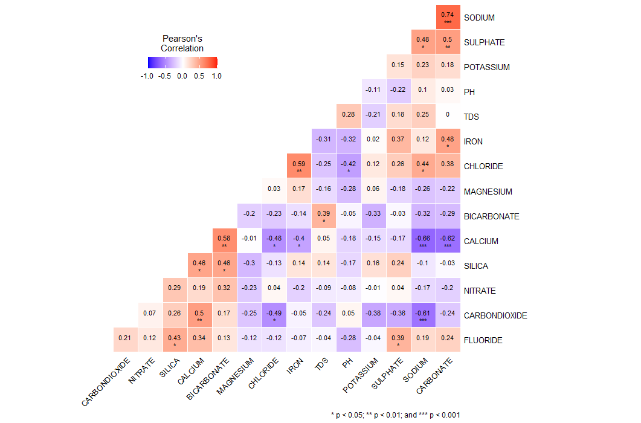


(5) Kishoreganj Sadar

(6) Madaripur Sadar


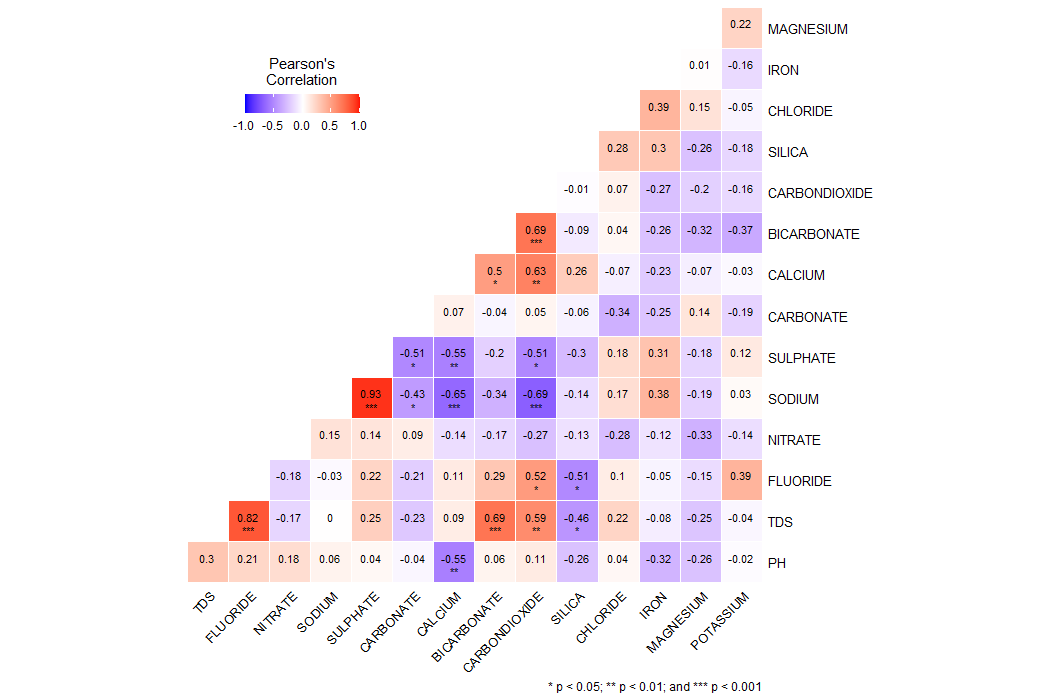


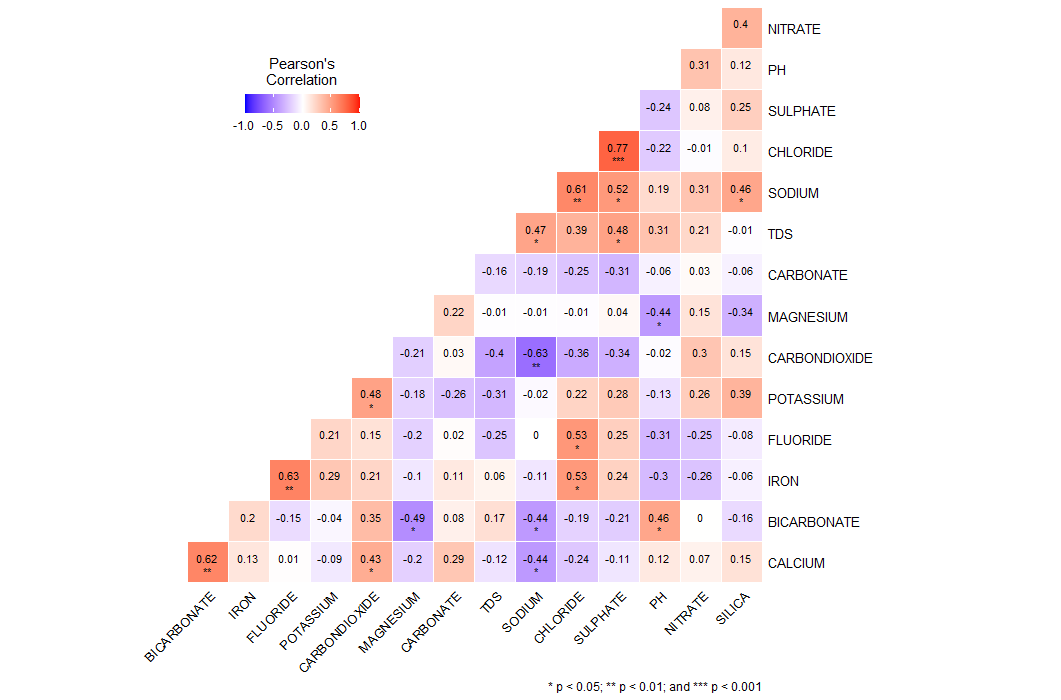


(8) Motijheel

(7) Mohammadpur


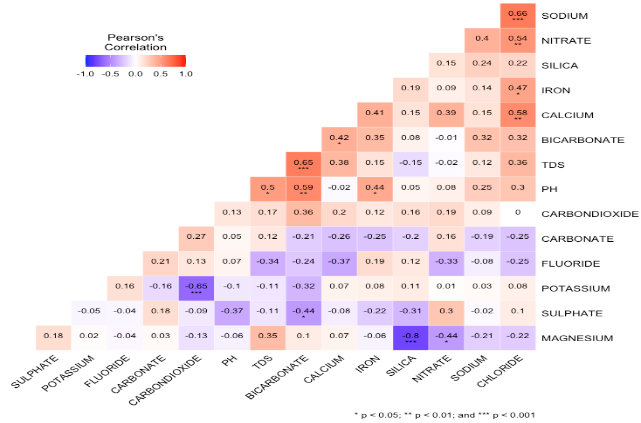


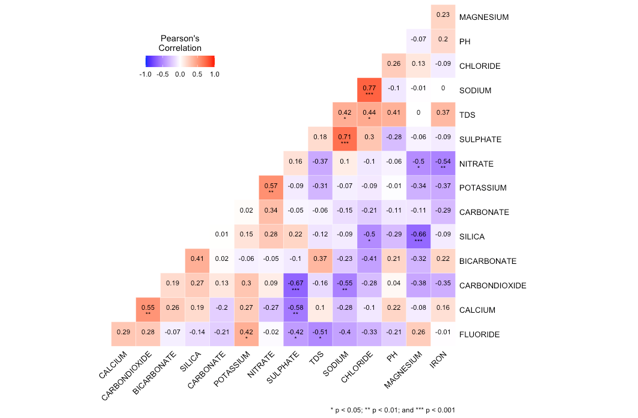


(10) Narsingdi Sadar

(9) Munshiganj


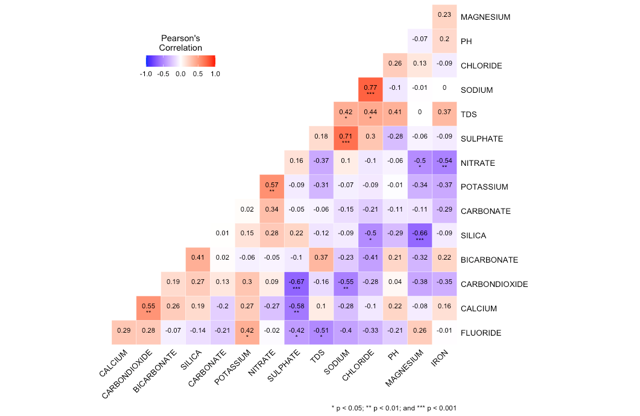


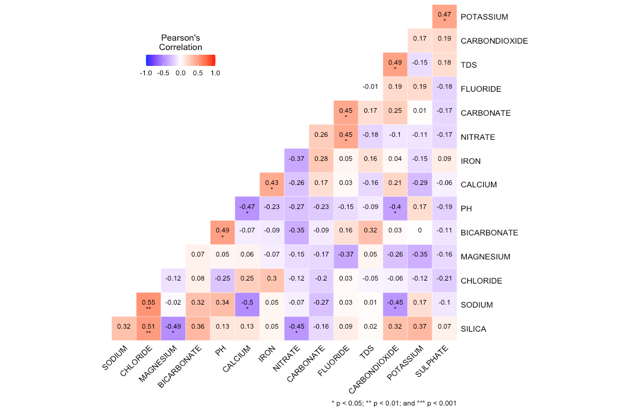


(11) Rajbari Pangsha

(12) Rajbari Sadar


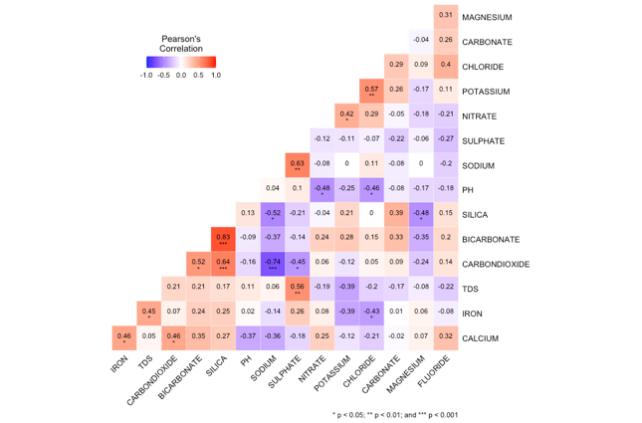

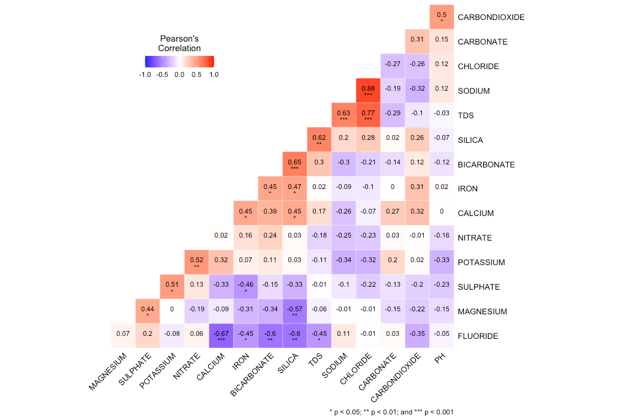


(14) Tangail Madhupur

(13) Sherpur Sadar


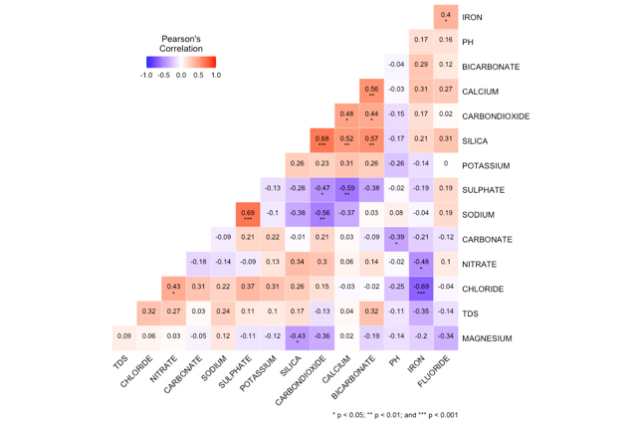


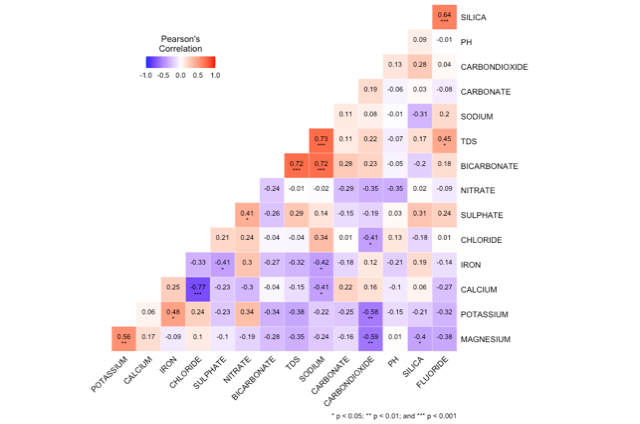


(16) Tangail Mirzapur

(15) Tangail Sadar


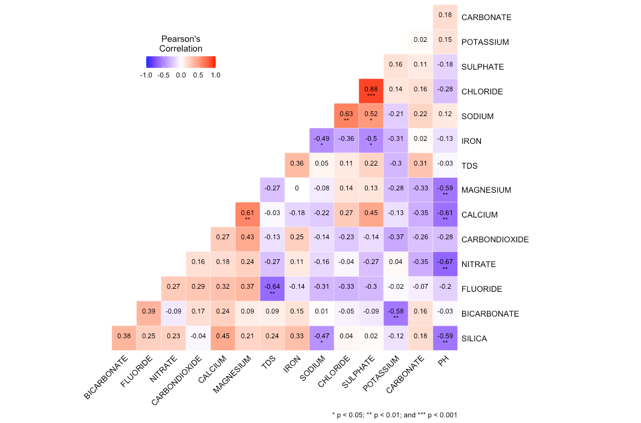


(17) Tangail Textile Mill

Appendix A2


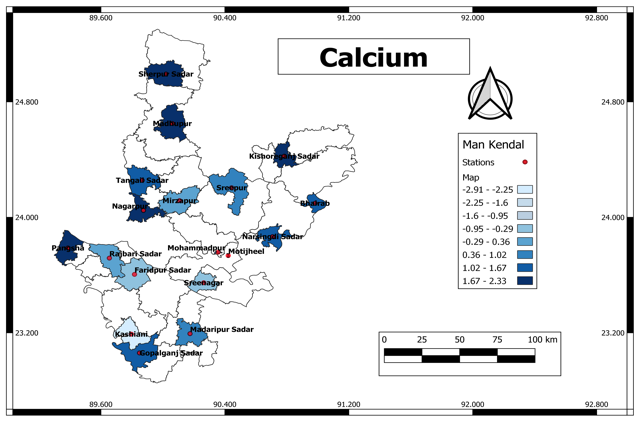

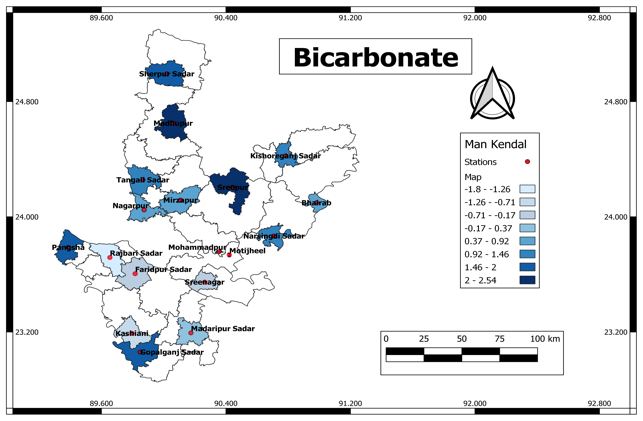


(2) Calcium

(1) Bicarbonate


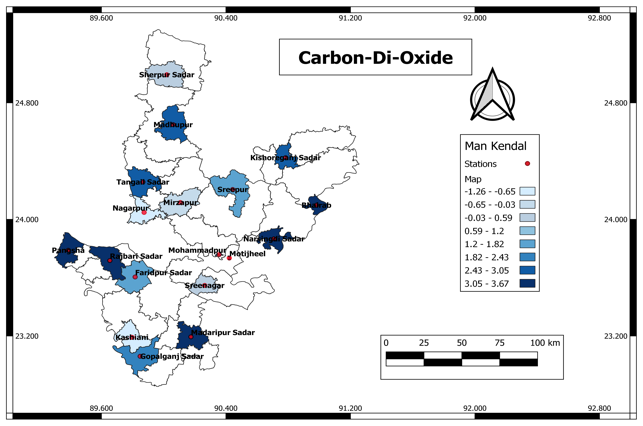


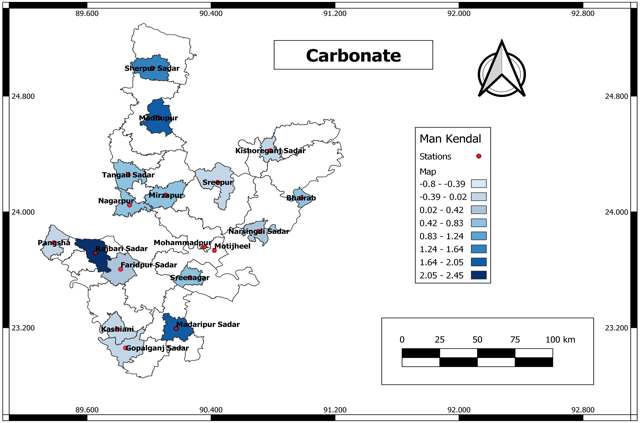


(4) Carbonate

(3) Carbondioxide


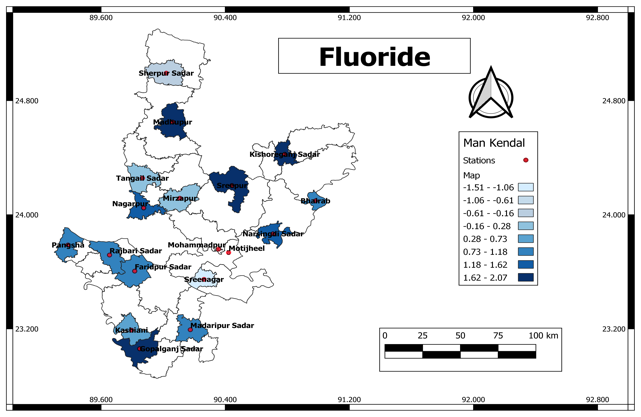

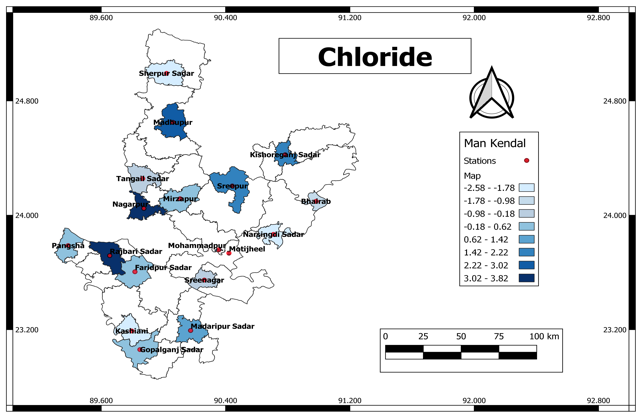


(5) Chloride

(6) Fluoride


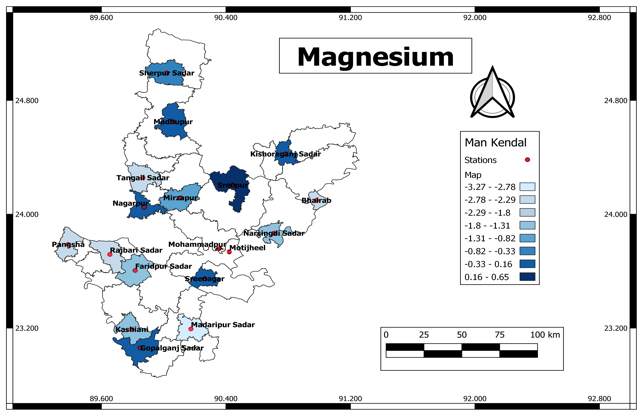

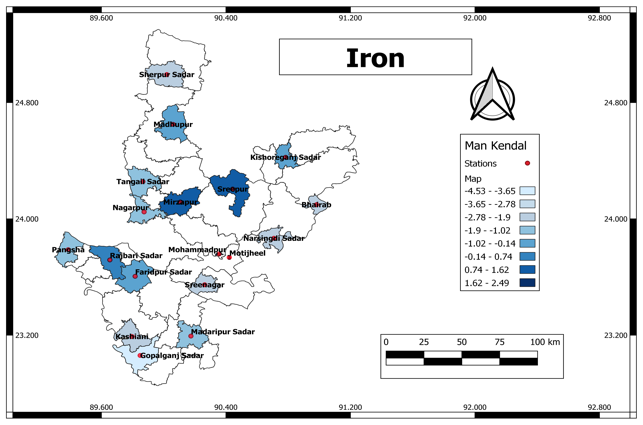


(8) Magnesium

(7) Iron


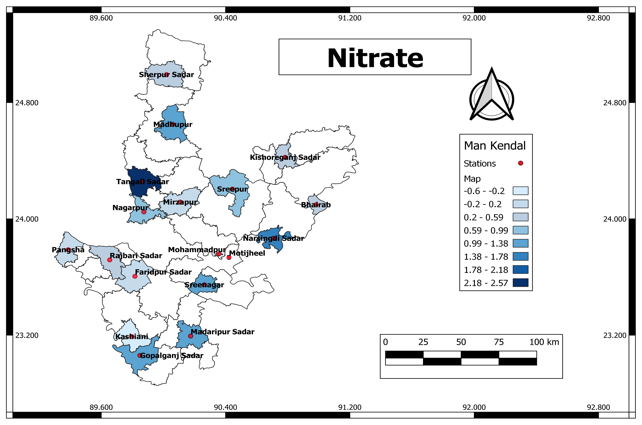


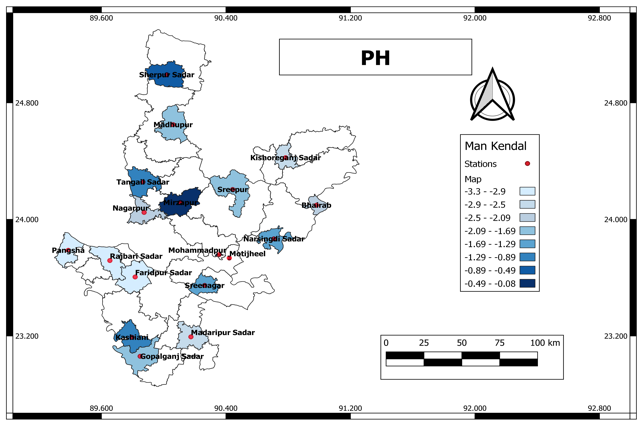


(10) PH

(9) Nitrate


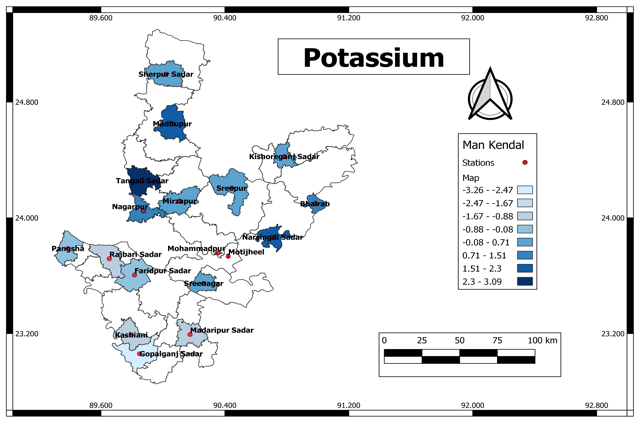

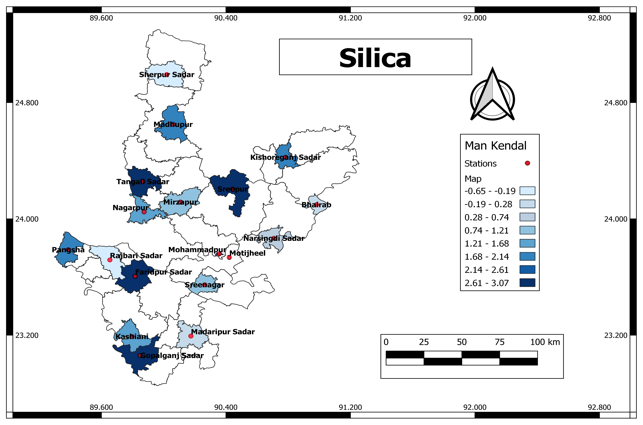


(11) Potasium

(12) Silica


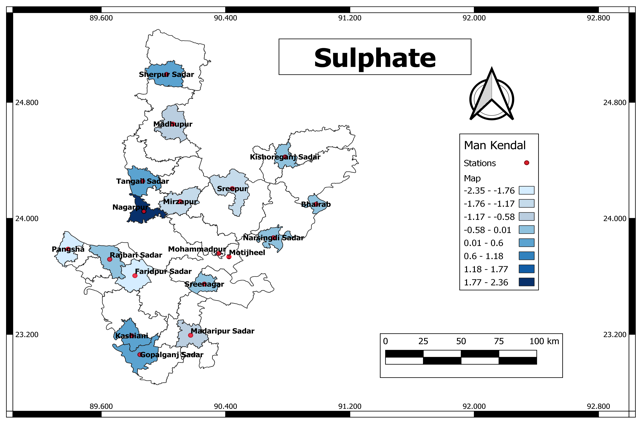

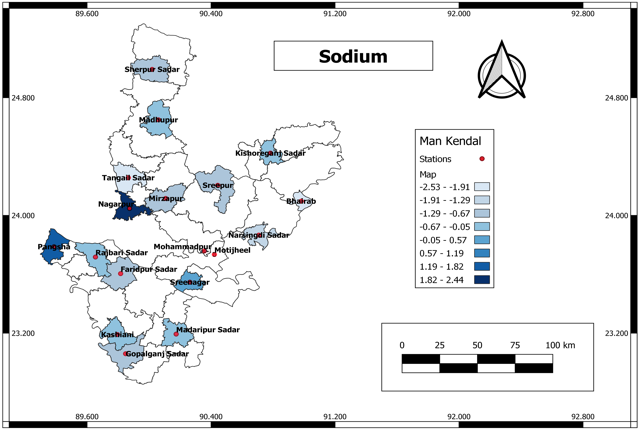


(14) Sulphate

(13) Sodium

Appendix A3


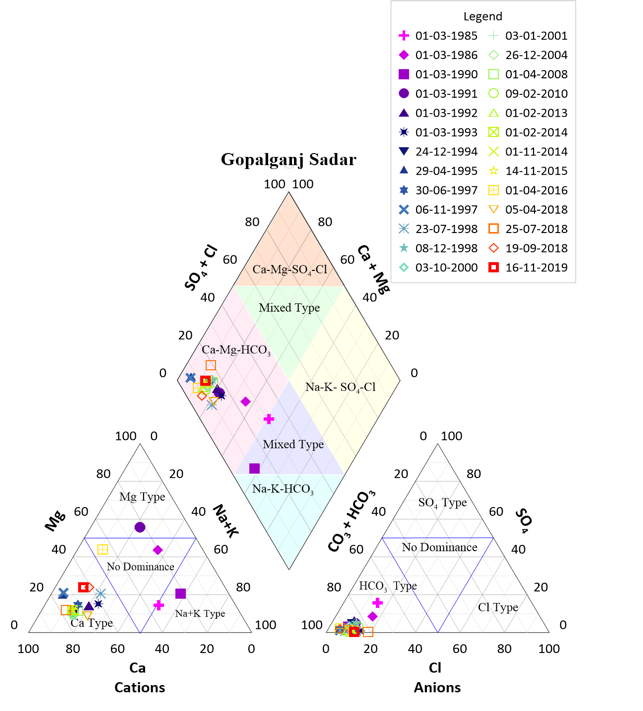

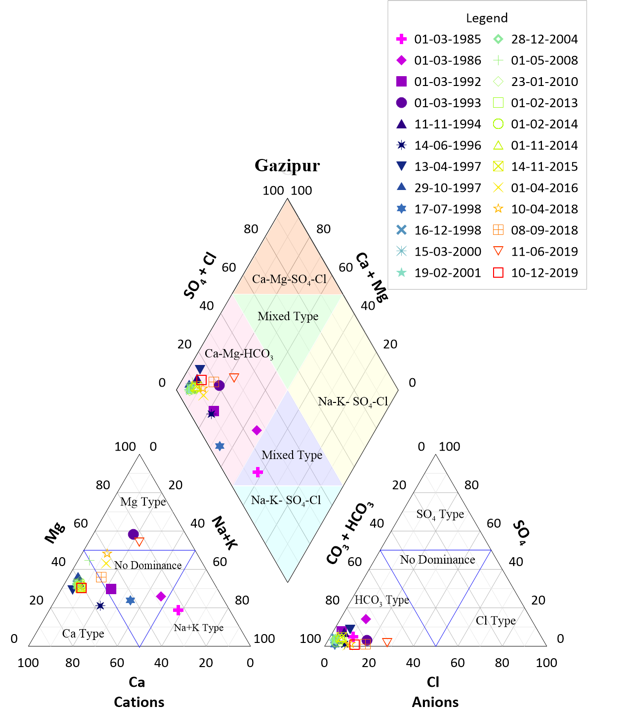


(2) Gopalganj Sadar

(1) Gazipur


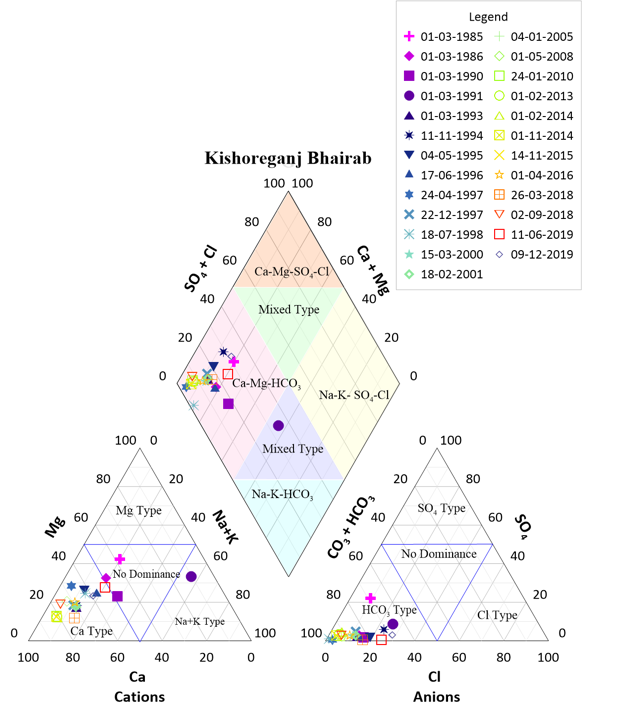

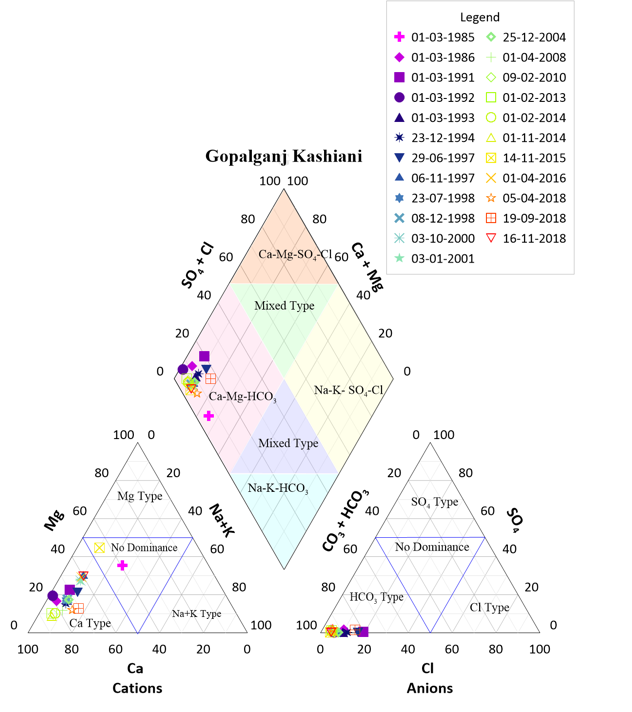


(4) Kishoreganj Bhairab

(3) Gopalganj Kashiani


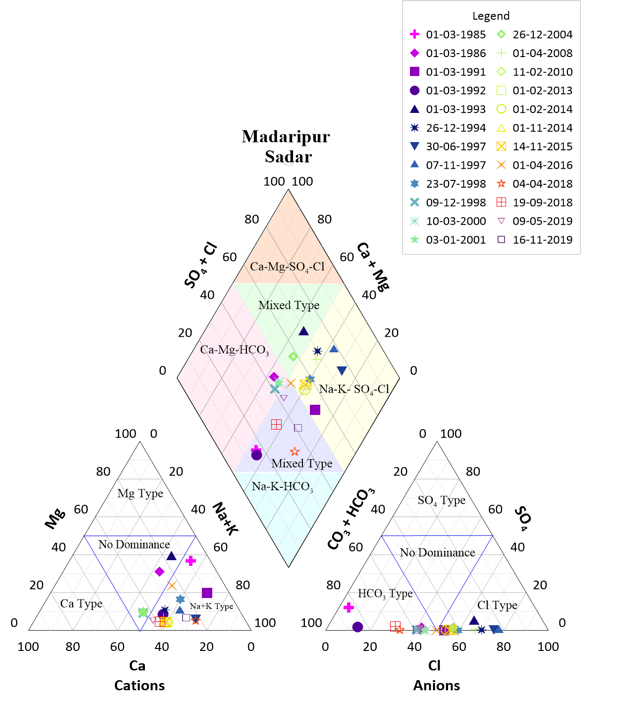

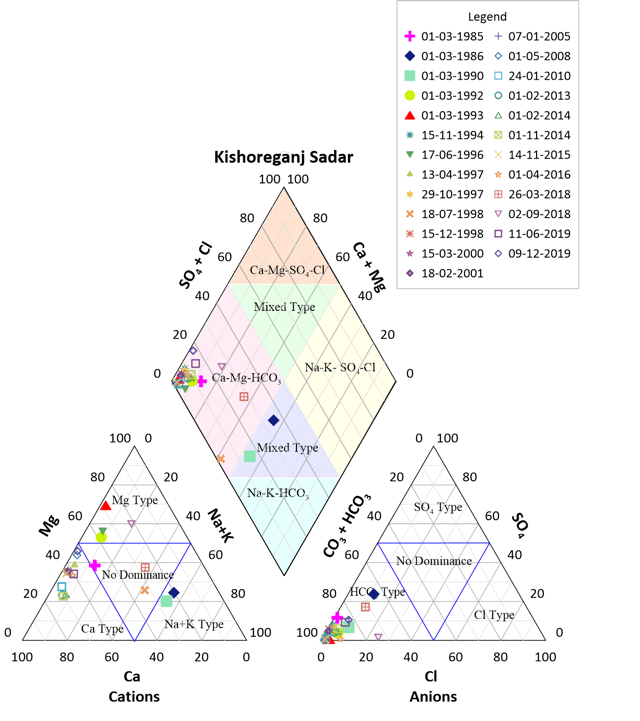


(5) Kishoreganj Sadar

(6) Madaripur Sadar


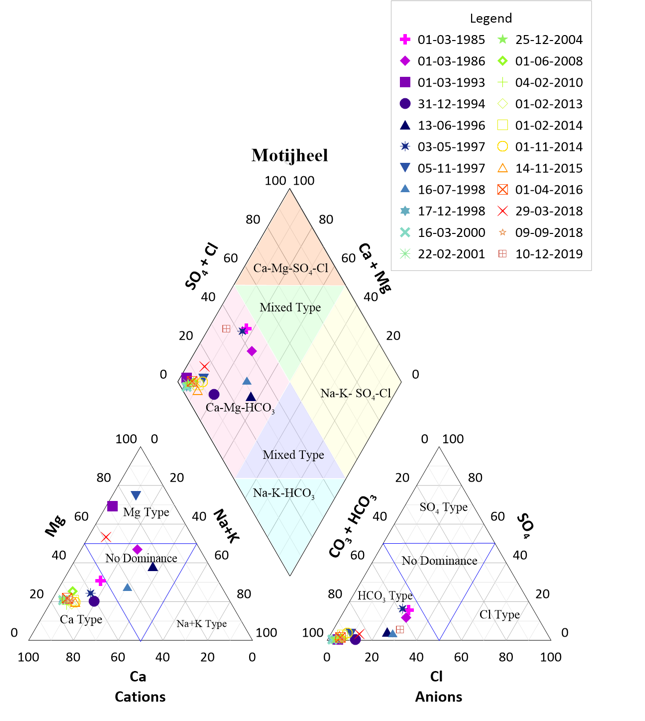

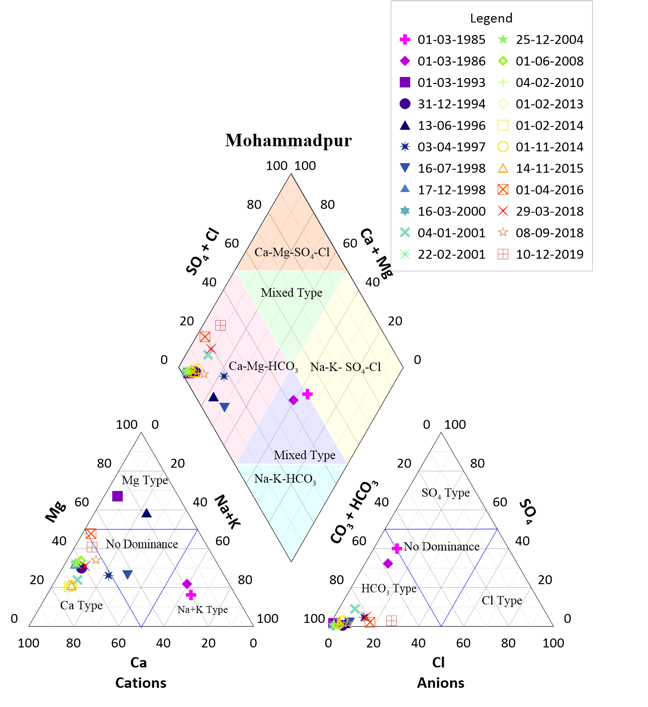


(7) Mohammadpur

(8) Motijheel


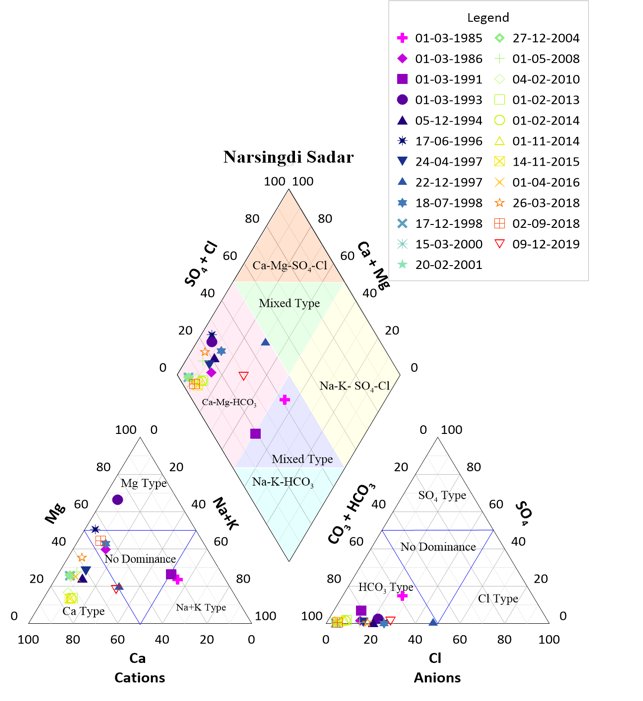

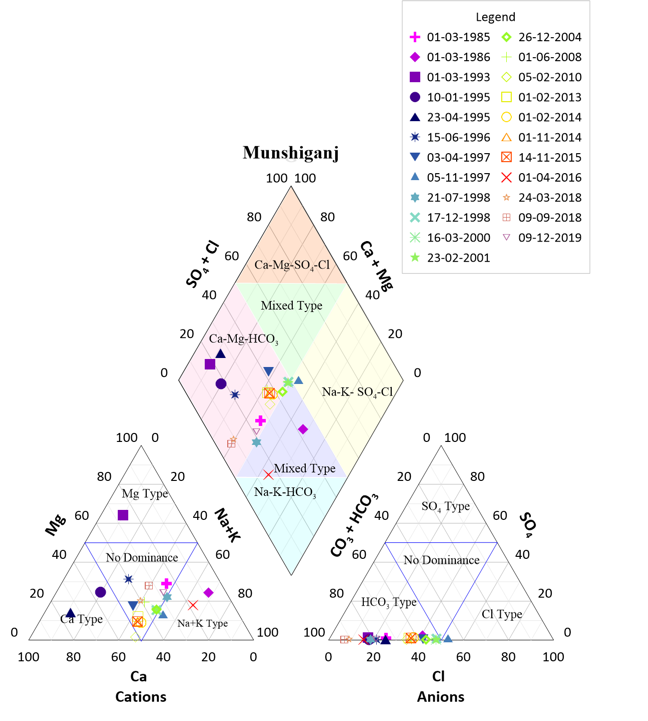


(10) Narsingdi Sadar

(9) Munshiganj


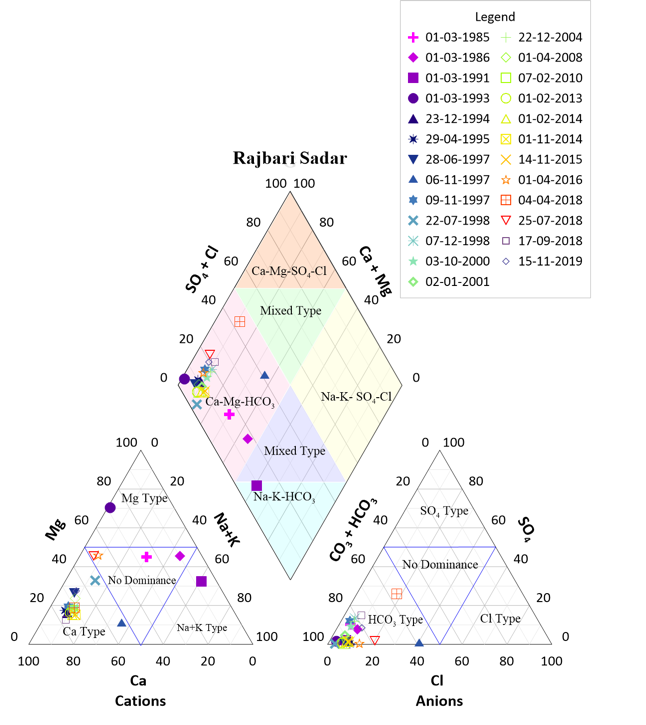

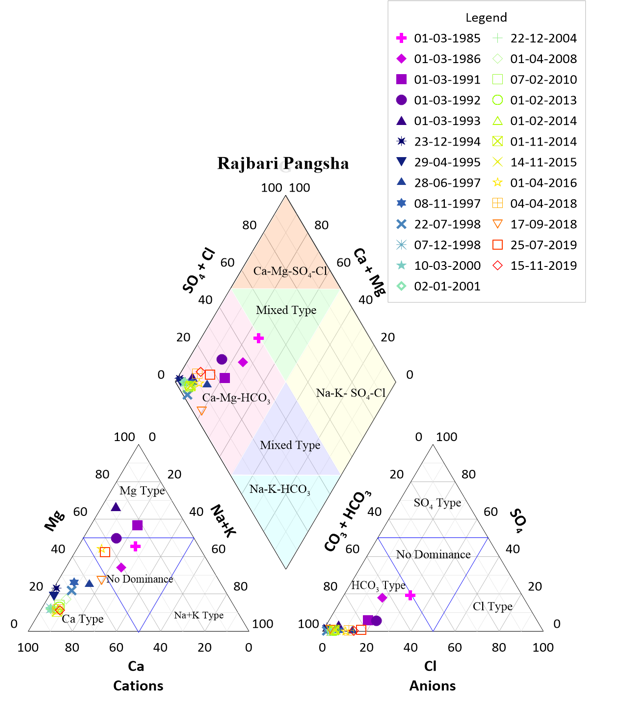


(11) Rajbari Pangsha

(12) Rajbari Sadar


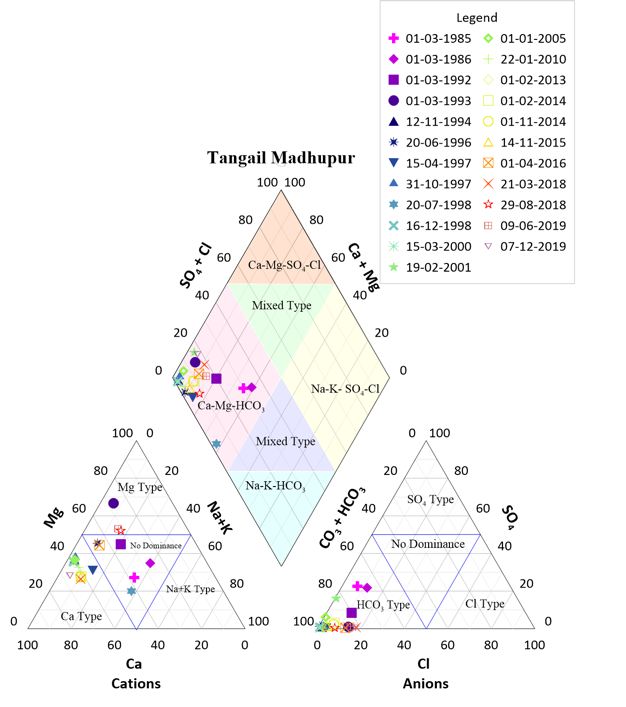

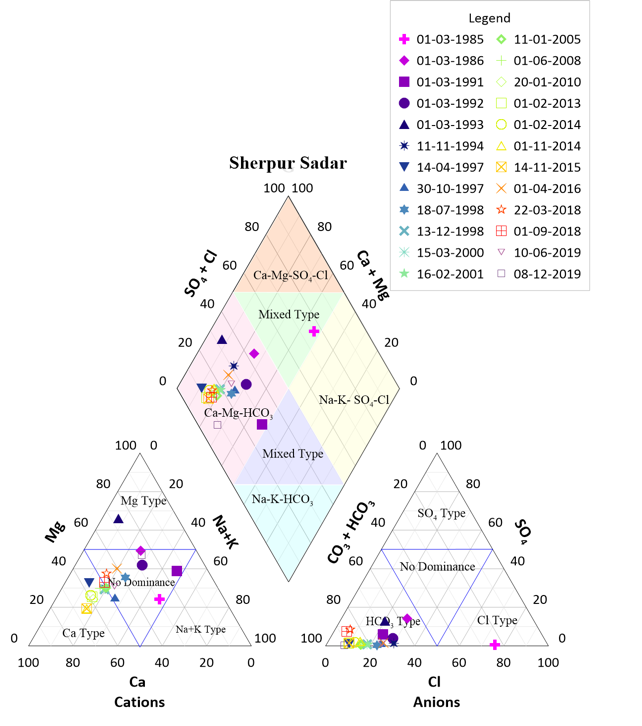


(14) Tangail Madhupur

(13) Sherpur Sadar


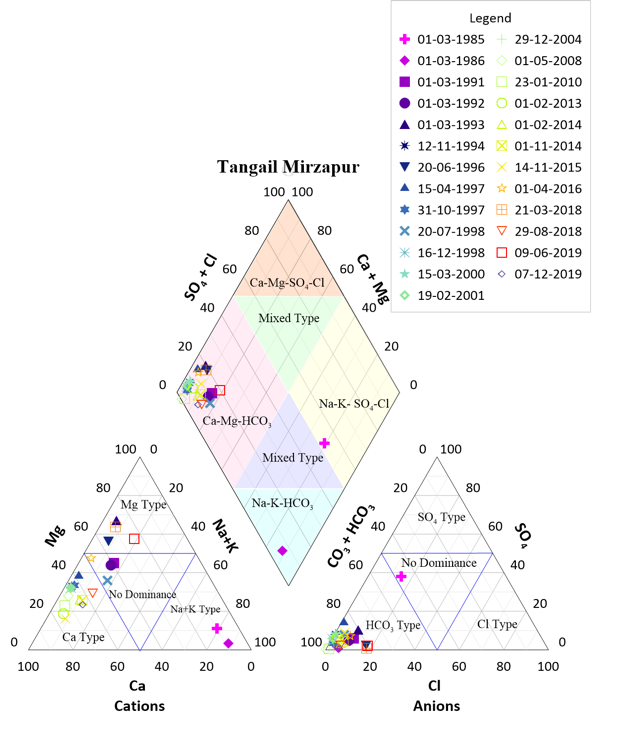

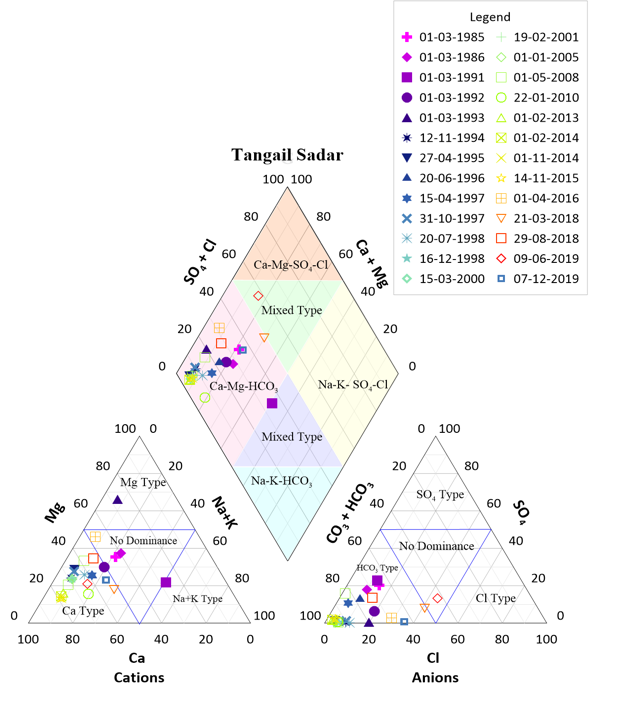


(15) Tangail Sadar

(16) Tangail Mirzapur


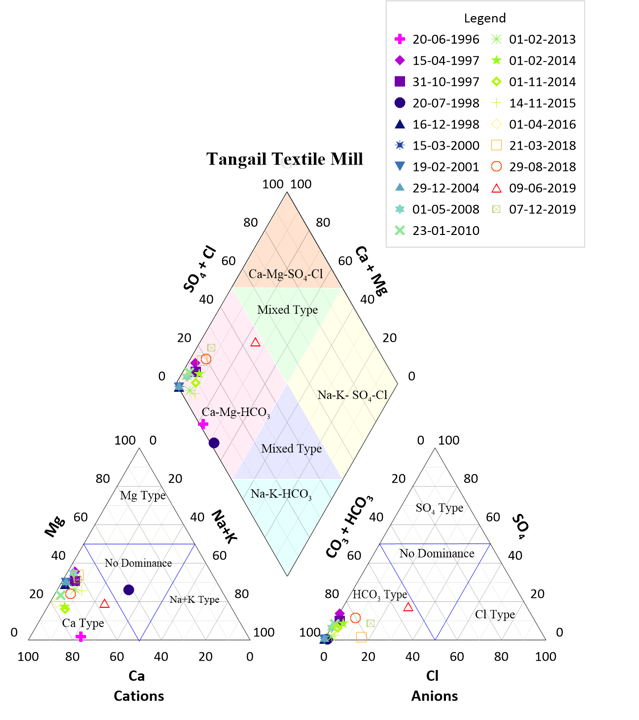


(17) Tangail Textile Mill

Appendix A4


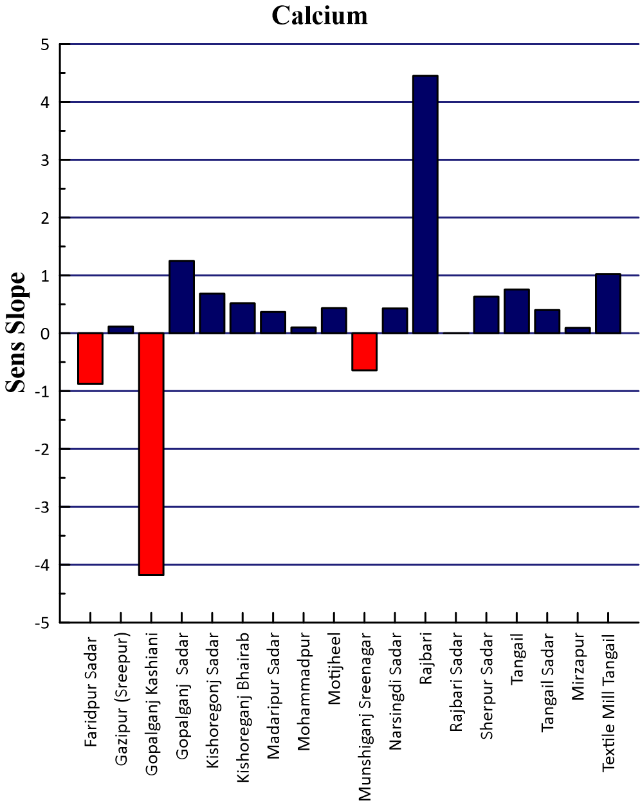

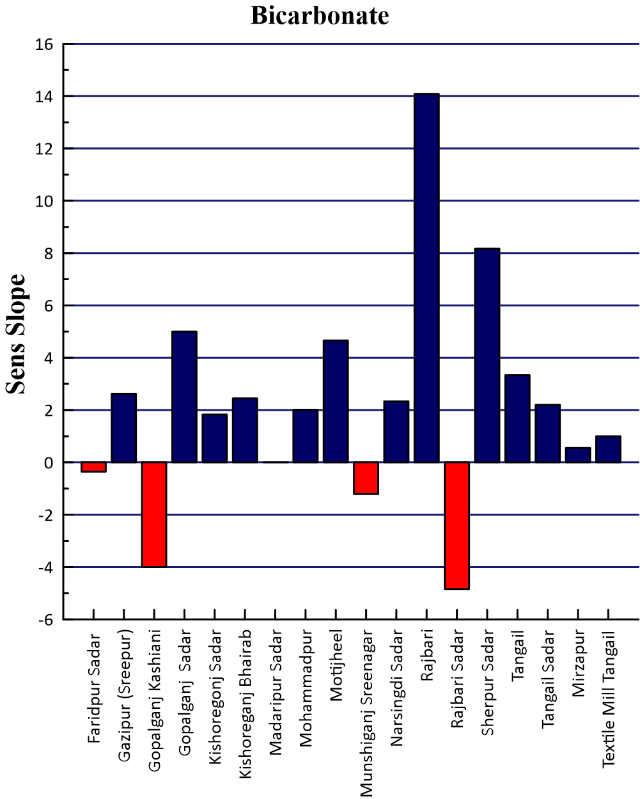


(2) Calcium

(1) Bicarbonate


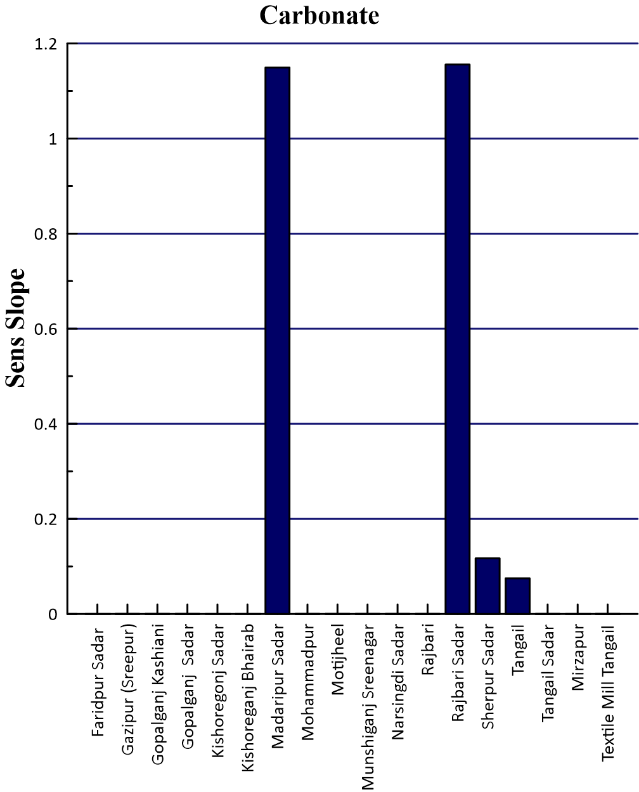

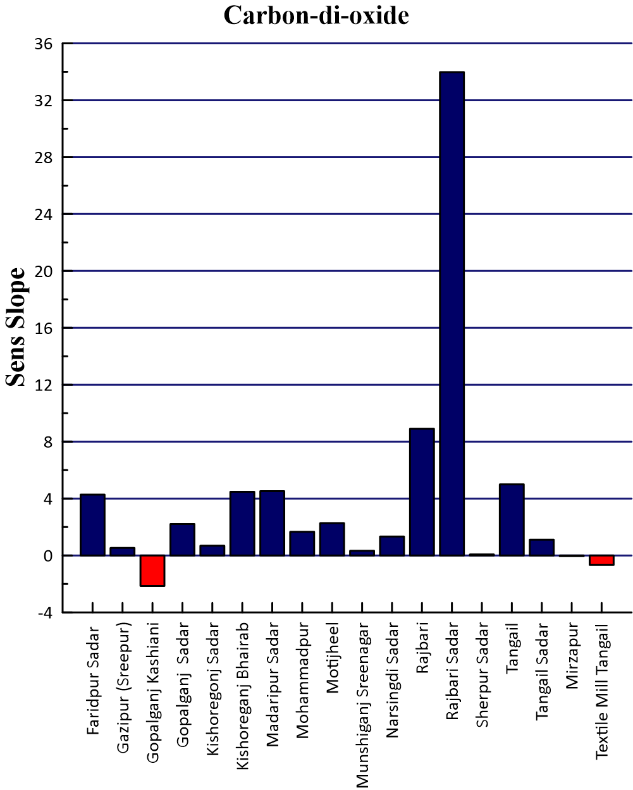


(4) Carbonate

(3) Carbondioxide


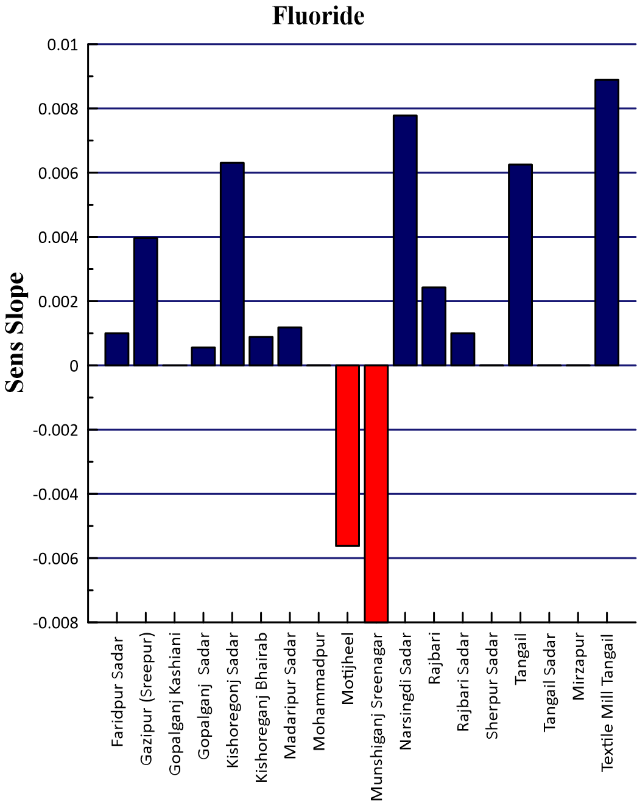

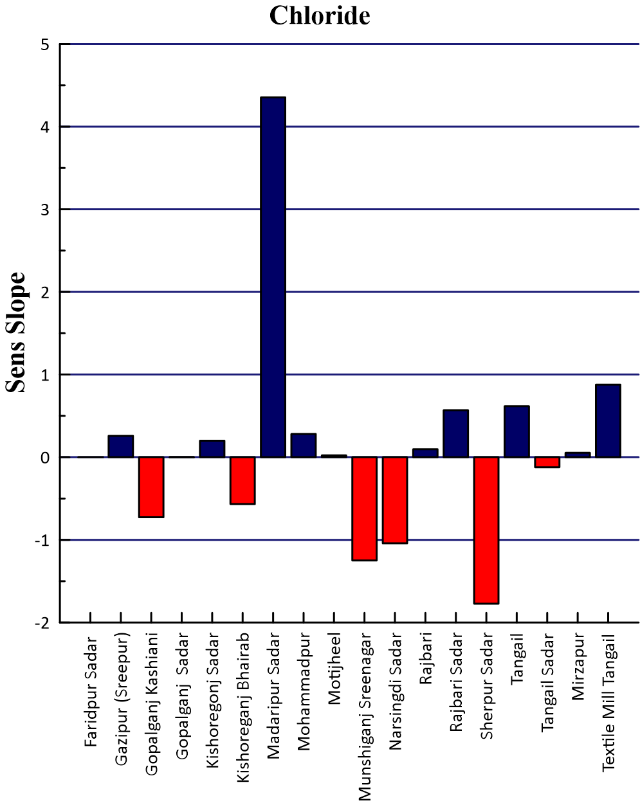


(6) Fluoride

(5) Chloride


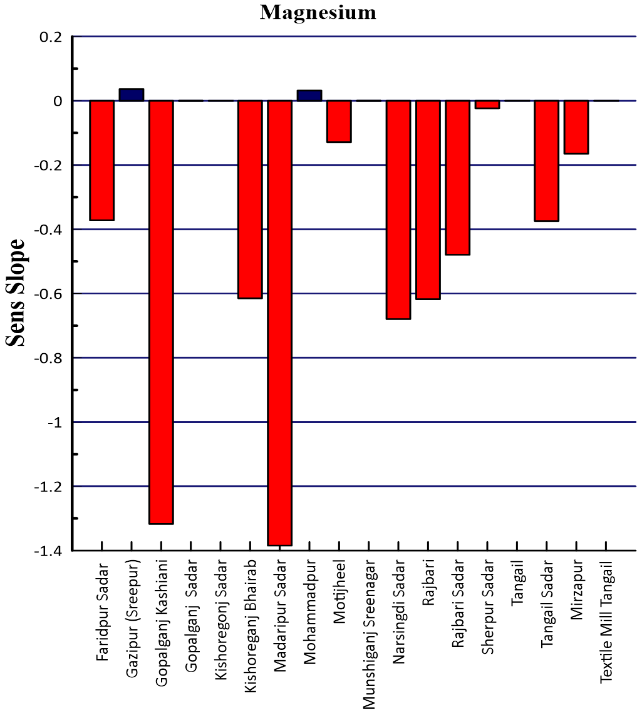

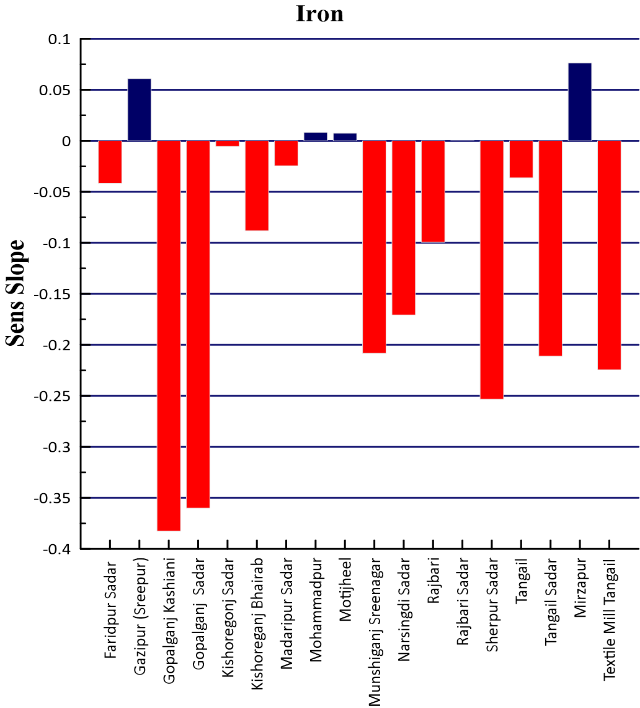


(8) Magnesium

(7) Iron


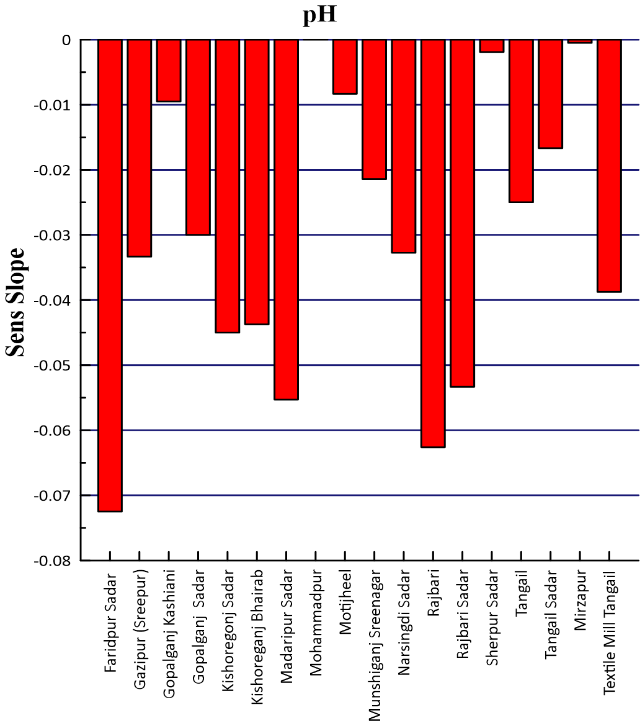

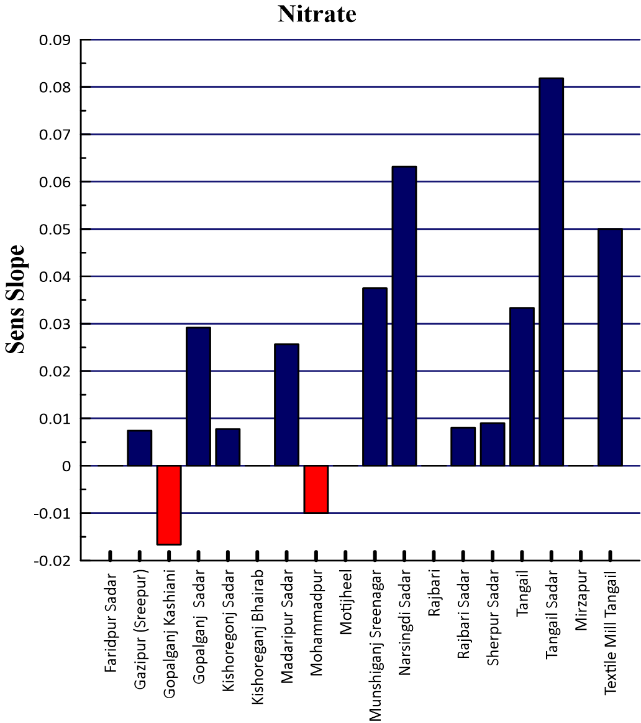


(10) PH

(9) Nitrate


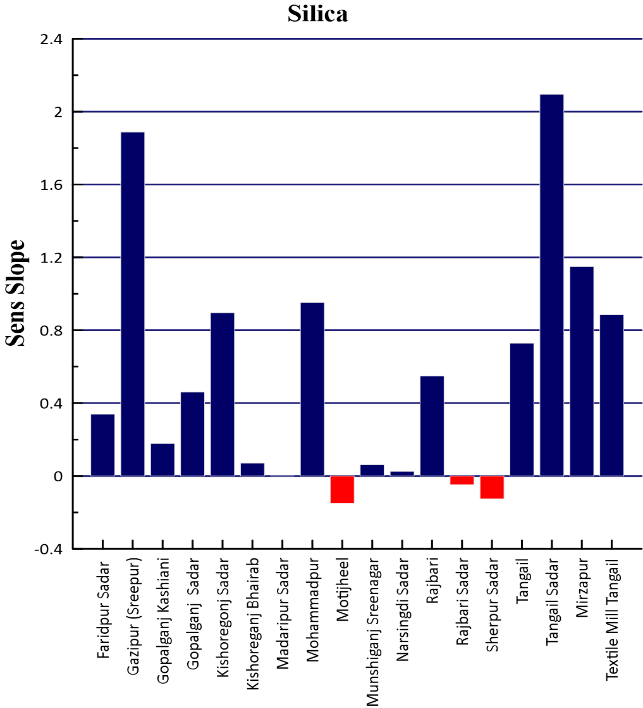

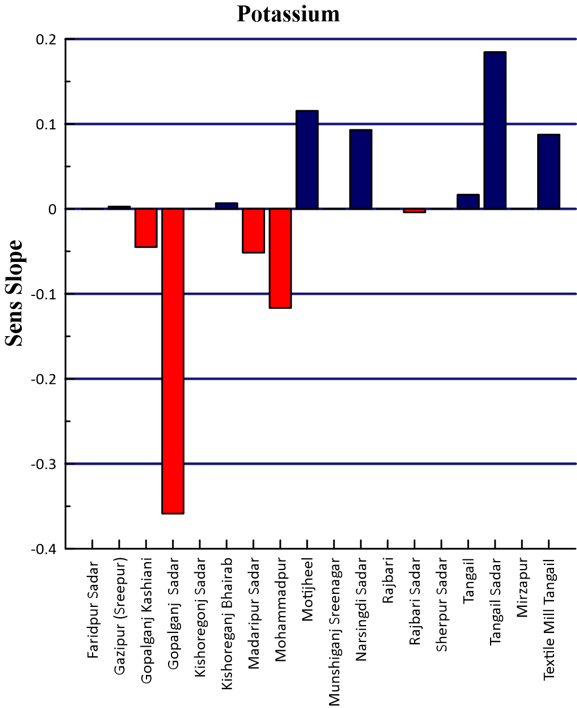


(11) Potasium

(12) Silica


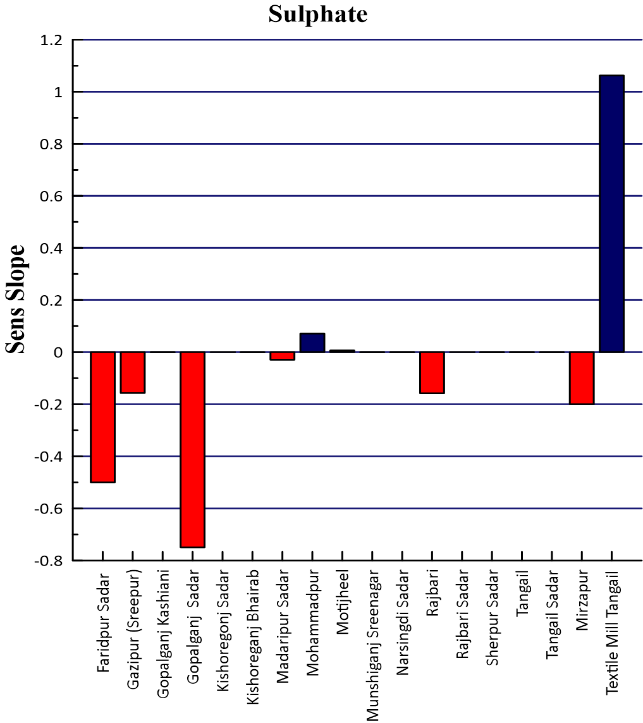

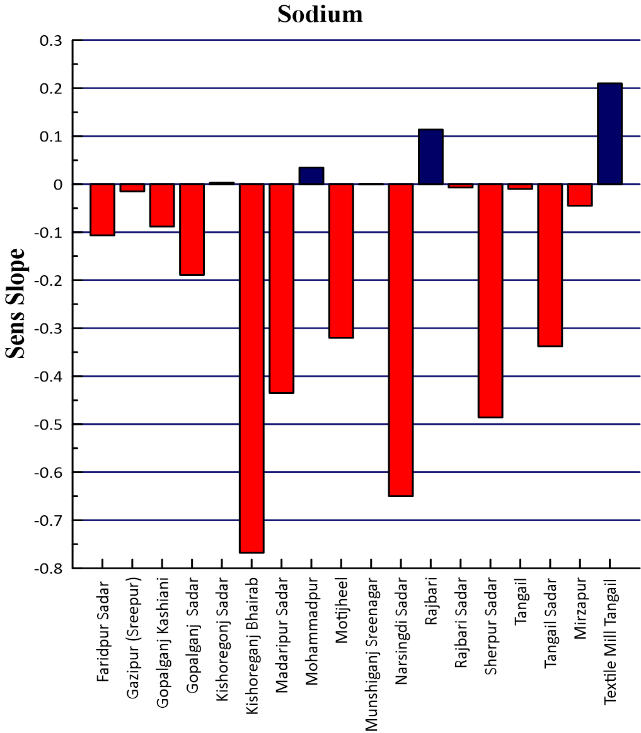


(14) Sulphate

(13) Sodium

Appendix A5


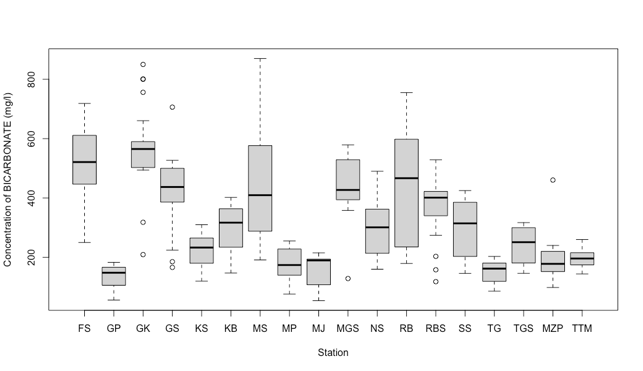


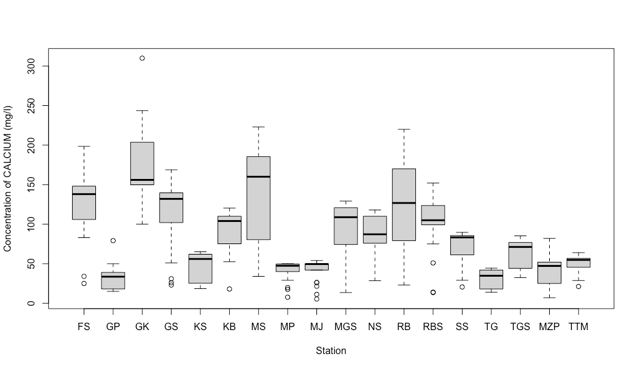


(2) Calcium

(1) Bicarbonate


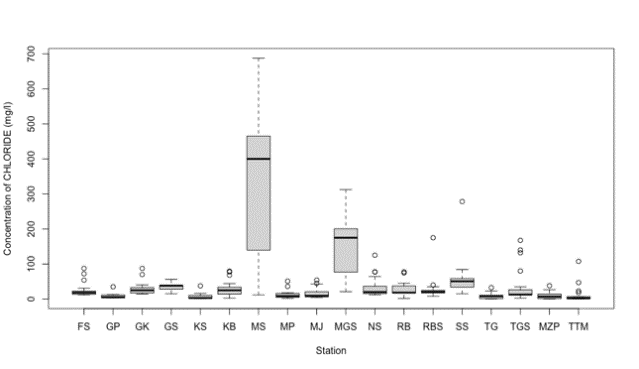

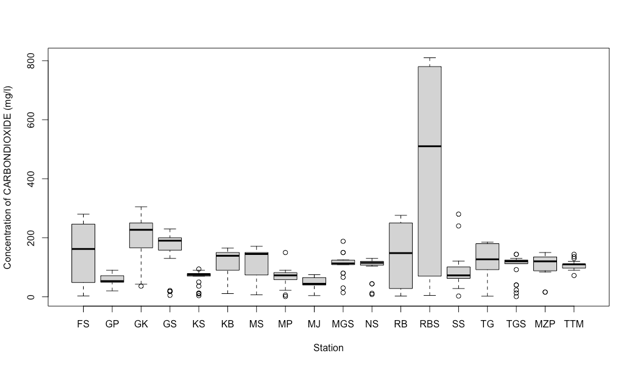


(4) Chloride

(3) Carbondioxide


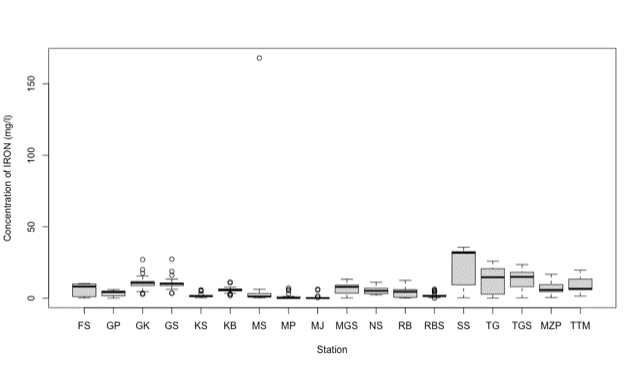

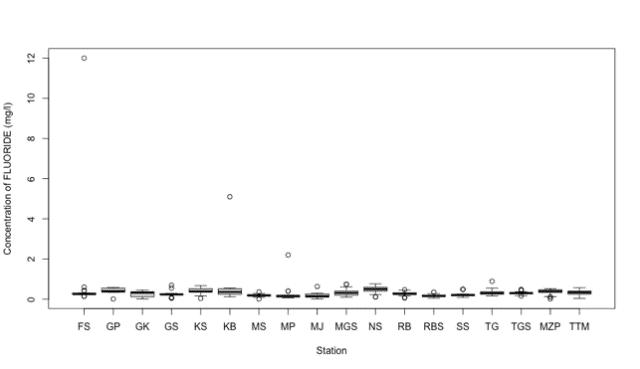


(6) Iron

(5) Fluoride


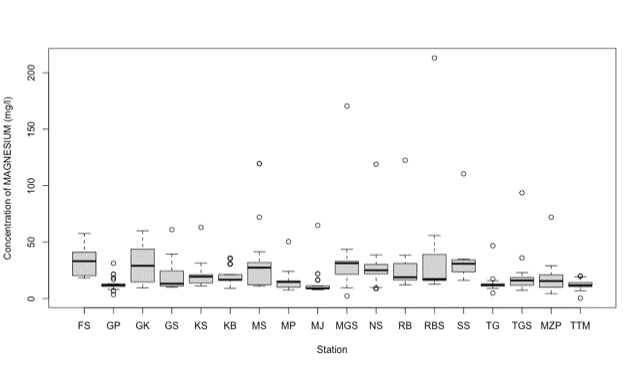


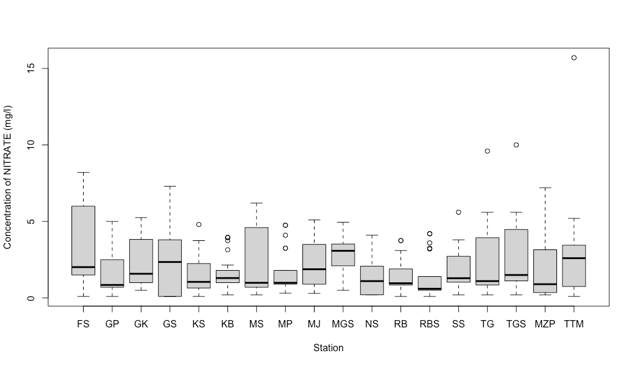


(8) Nitrate

(7) Magnesium


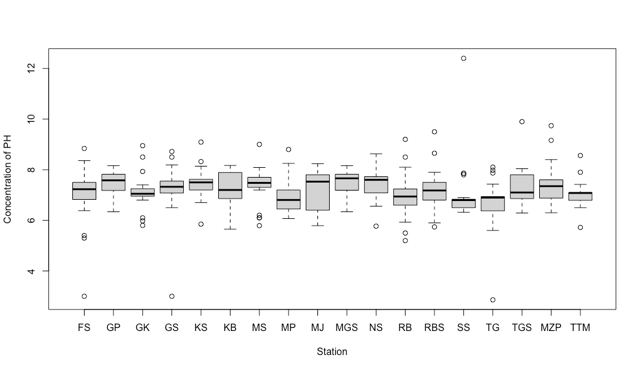


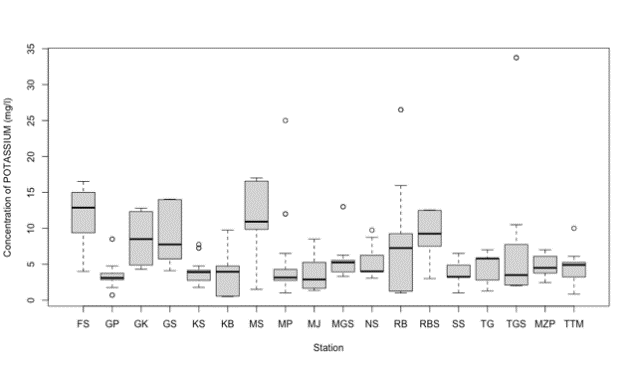


(10) Potasium

(9) PH


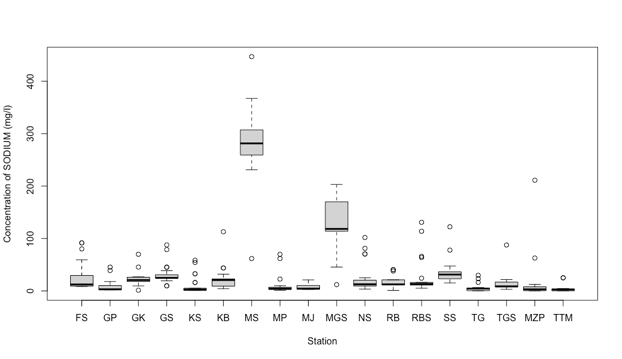

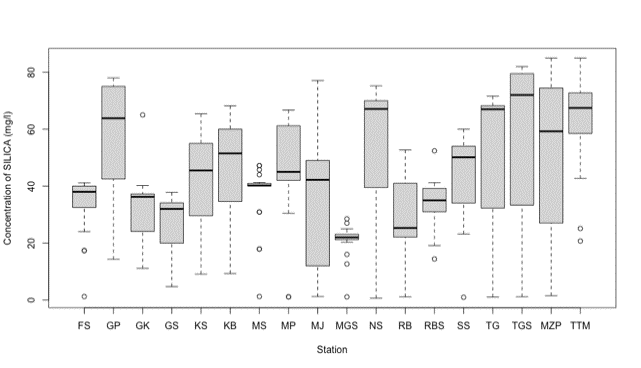


(12) Sodium

(11) Silica


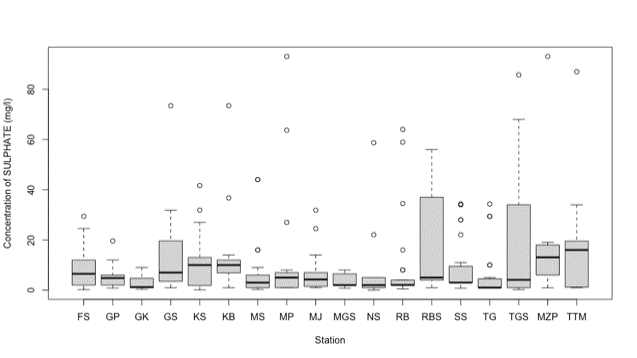


(13) Sulphate

Appendix A6

1. Faridpur Sadar

1. Gazipur

1. Gopalganj Kashiani

1. Gopalganj Sadar

1. Kishoreganj Bhairab

1. Kishoregank Sadar

1. Madaripur Sadar
2. Mohammadpur

1. Motijheel

1. Monshiganj Sreenahar

1. Narsingdi Sadar

1. Rajbari

1. Rajbari Sadar
2. Sherpur Sadar

1. Tangail

1. Tangail Sadar
2. Mirzapur

1. Taxtile Mill Tangail

Appendix A7

`
