# Supplementary material for: GIS based geostatistical modelling and trends analysis of groundwater quality for suitable uses in Dhaka division
Source: Sci Rep. 2024 Jul 29;14:17449. doi: 10.1038/s41598-024-66567-z (PMC11286770; doi:10.1038/s41598-024-66567-z)
Supplement: Supplementary file 2 — Supplementary Information 2. [file 41598_2024_66567_MOESM2_ESM.docx]

**Supplementary Material**

**Table A1 Groundwater Quality of Sherpur Sadar**

| **SL** | **WELL ID** | **DATE** | **TDS** | **CALCIUM** | **MAGNESIUM** | **SODIUM** | **POTASSIUM** | **CHLORIDE** | **CARBONATE** | **BICARBONATE** | **SULPHATE** | **NITRATE** | **IRON** | **SILICA** | **FLUORIDE** | **CARBONDIOXIDE** | **pH** |
| --- | --- | --- | --- | --- | --- | --- | --- | --- | --- | --- | --- | --- | --- | --- | --- | --- | --- |
| 1 | GQ8988042 | 01-03-1985 | 655 | 67.5 | 33.94 | 122.36 | 3.2 | 278.3 | 1.76 | 145.8 | 3 | 0.8 | 11.36 | 60 | 0.24 | 2.5 | 6.8 |
| 2 | GQ8988042 | 01-03-1986 | 307 | 29.05 | 34.93 | 31.42 | 4.75 | 52.68 | 11.69 | 149.4 | 34.3 | 0.2 | 7.25 | 25.2 | 0.49 | 65 | 6.8 |
| 3 | GQ8988042 | 01-03-1991 | 406 | 20.6 | 34.44 | 77.8 | 3.2 | 61.62 | 11.69 | 307.9 | 22.04 | 2.2 | 35.6 | 51.1 | 0.23 | 40.45 | 6.5 |
| 4 | GQ8988042 | 01-03-1992 | 256 | 31 | 28 | 36 | 3.25 | 57 | 12.00 | 212 | 11 | 5.6 | 12.8 | 23.7 | 0.49 | 65 | 6.9 |
| 5 | GQ8988042 | 01-03-1993 | 280 | 73.6 | 110.4 | 20.8 | 4.75 | 32 | 11.69 | 160 | 28 | 1.6 | 12.8 | 0.96 | 0.23 | 28 | 6.32 |
| 6 | GQ8988042 | 11-11-1994 | 310 | 85.2 | 30.52 | 36.07 | 3.25 | 55.5 | 12 | 194 | 3 | 1.01 | 35.5 | 52.6 | 0.17 | 99 | 6.81 |
| 7 | GQ8988042 | 14-04-1997 | 310 | 81.6 | 28.57 | 15.28 | 4.75 | 23.5 | 68 | 220 | 3 | 2.2 | 32.25 | 51.1 | 0.15 | 280 | 6.74 |
| 8 | GQ8988042 | 30-10-1997 | 320 | 79.6 | 24.17 | 47.45 | 3.2 | 73 | 68 | 244 | 2.95 | 1.7 | 33 | 42.9 | 0.2 | 240 | 12.4 |
| 9 | GQ8988042 | 18-07-1998 | 304 | 41 | 22.59 | 29.8 | 2.32 | 45.9 | 11.69 | 242 | 0.79 | 0.2 | 19.59 | 23.17 | 0.24 | 60 | 7.86 |
| 10 | GQ8988042 | 13-12-1998 | 420 | 88 | 30.52 | 36.14 | 3.25 | 55.6 | 1.76 | 422 | 3 | 1.05 | 35.6 | 55 | 0.18 | 100 | 6.82 |
| 11 | GQ8988042 | 15-03-2000 | 354 | 88 | 31 | 36.4 | 3.25 | 56 | 2.8 | 420 | 3 | 1.05 | 35.6 | 55 | 0.18 | 102 | 6.8 |
| 12 | GQ8988042 | 16-02-2001 | 425 | 85 | 31 | 36.5 | 3.2 | 60 | 11.69 | 425 | 3 | 1.05 | 32 | 60 | 0.18 | 100 | 6.8 |
| 13 | GQ8988042 | 11-01-2005 | 420 | 85 | 31 | 36.5 | 3.2 | 60.2 | 68 | 422 | 3 | 1.05 | 32.2 | 60 | 0.18 | 100 | 6.8 |
| 14 | GQ8988042 | 01-06-2008 | 354 | 89.6 | 30.64 | 31.1 | 3.76 | 48.58 | 68 | 321 | 2.95 | 1.38 | 35.14 | 53 | 0.12 | 121 | 6.47 |
| 15 | GQ8988042 | 20-01-2010 | 304 | 79.2 | 19.77 | 24.5 | 2.5 | 37 | 1.76 | 338 | 3 | 1.5 | 32.15 | 48 | 0.15 | 80 | 6.85 |
| 16 | GQ8988042 | 01-02-2013 | 325 | 85.2 | 22.41 | 23.23 | 5 | 35.75 | 96 | 367 | 6 | 3.25 | 31 | 52.65 | 0.18 | 65 | 6.7 |
| 17 | GQ8988042 | 01-02-2014 | 330 | 86 | 23.14 | 21.12 | 6.5 | 32.5 | 11.69 | 373 | 7 | 3.8 | 32 | 51.15 | 0.2 | 60 | 6.5 |
| 18 | GQ8988042 | 01-11-2014 | 320 | 88.8 | 16.08 | 22.75 | 6.1 | 35 | 2.8 | 345 | 8 | 3.5 | 31.75 | 49.15 | 0.23 | 65 | 6.4 |
| 19 | GQ8988042 | 14-11-2015 | 320 | 88.8 | 16.08 | 22.75 | 6.1 | 35 | 96 | 345 | 8 | 3.5 | 31.75 | 49.15 | 0.23 | 65 | 6.4 |
| 20 | GQ8988042 | 01-04-2016 | 480 | 55 | 33 | 30 | 1 | 84 | 11.69 | 398 | 7 | 1.05 | 0.2 | 59.3 | 0.15 | 98 | 7.8 |
| 21 | GQ8988042 | 22-03-2018 | 406 | 67.5 | 33 | 23.23 | 6.5 | 20 | 2.80 | 422 | 34 | 3.25 | 2.55 | 48 | 0.09 | 102 | 6.8 |
| 22 | GQ8988042 | 01-09-2018 | 354 | 85.2 | 34.44 | 31.1 | 6.1 | 16 | 96.00 | 220 | 28 | 1.2 | 3.6 | 48 | 0.26 | 102 | 6.48 |
| 23 | GQ8988042 | 10-06-2019 | 304 | 85 | 34.44 | 47.45 | 2.5 | 55 | 68.00 | 146 | 3 | 0.5 | 4.91 | 25.2 | 0.26 | 65 | 7.86 |
| 24 | GQ8988042 | 08-12-2019 | 133 | 31 | 34.44 | 36.5 | 2.5 | 15 | 68 | 146 | 1 | 0.8 | 2.97 | 23.7 | 0.49 | 40.45 | 6.75 |

Note: All units are mg/L except pH (-).

**Table A2 Groundwater Quality of Rajbari (Pangsha)**

| **SL** | **WELL ID** | **DATE** | **TDS** | **CALCIUM** | **MAGNESIUM** | **SODIUM** | **POTASSIUM** | **CHLORIDE** | **CARBONATE** | **BICARBONATE** | **SULPHATE** | **NITRATE** | **IRON** | **SILICA** | **FLUORIDE** | **CARBONDIOXIDE** | **pH** |
| --- | --- | --- | --- | --- | --- | --- | --- | --- | --- | --- | --- | --- | --- | --- | --- | --- | --- |
| 1 | GQ8273069 | 01-03-1985 | 380 | 40 | 38.4 | 40.71 | 1.25 | 74.55 | 17.59 | 179.3 | 64 | 0.8 | 7.61 | 18.7 | 0.32 | 2.39 | 6.6 |
| 2 | GQ8273069 | 01-03-1986 | 388 | 61.13 | 30.98 | 37.96 | 9.25 | 44.2 | 17.66 | 233.1 | 58.93 | 1.5 | 0.1 | 18.7 | 0.23 | 3.99 | 6.98 |
| 3 | GQ8273069 | 01-03-1991 | 689 | 23 | 35.7 | 19.89 | 9.25 | 77.53 | 55.08 | 466.6 | 34.5 | 0.8 | 0.56 | 18.7 | 0.32 | 13.47 | 8.5 |
| 4 | GQ8273069 | 01-03-1992 | 400 | 45 | 38.4 | 21.23 | 1.25 | 45 | 40 | 179.3 | 16 | 3.1 | 6 | 41 | 0.32 | 3.99 | 7.1 |
| 5 | GQ8273069 | 01-03-1993 | 262 | 81.6 | 122.4 | 6.5 | 26.5 | 10 | 21.00 | 235 | 8 | 1 | 12.5 | 1.1 | 0.23 | 28 | 7.45 |
| 6 | GQ8273069 | 23-12-1994 | 240 | 101.6 | 18.8 | 1.04 | 1.2 | 1.6 | 21 | 217 | 4 | 0.8 | 6.5 | 25.2 | 0.06 | 20 | 9.2 |
| 7 | GQ8273069 | 29-04-1995 | 250 | 97.2 | 13.92 | 2.6 | 1.25 | 4 | 64.8 | 250 | 2 | 1.1 | 7.25 | 23 | 0.32 | 62 | 7.24 |
| 8 | GQ8273069 | 28-06-1997 | 372 | 126.8 | 32.23 | 21.23 | 26.5 | 32.5 | 21 | 321 | 1 | 1.9 | 0.2 | 52.7 | 0.47 | 126 | 7.39 |
| 9 | GQ8273069 | 08-11-1997 | 400 | 126.8 | 30.27 | 11.38 | 9.25 | 17.5 | 21 | 357 | 2 | 1 | 0.19 | 38.4 | 0.19 | 82 | 7.19 |
| 10 | GQ8273069 | 22-07-1998 | 345 | 79.18 | 15.05 | 9.9 | 2.8 | 6 | 64.8 | 398 | 0.47 | 2.5 | 2.26 | 10.94 | 0.16 | 148 | 8.02 |
| 11 | GQ8273069 | 07-12-1998 | 280 | 213.2 | 19 | 11.38 | 1.25 | 17.5 | 21 | 743 | 2.05 | 0.9 | 6.02 | 25.3 | 0.23 | 276 | 6.69 |
| 12 | GQ8273069 | 10-03-2000 | 270 | 216 | 18.7 | 11.4 | 1.25 | 17.55 | 64.8 | 750 | 2.05 | 0.85 | 6 | 26.3 | 0.23 | 270 | 6.7 |
| 13 | GQ8273069 | 02-01-2001 | 280 | 216 | 18.7 | 11.4 | 1.25 | 17.55 | 4.4 | 750 | 2.05 | 0.85 | 6 | 25 | 0.23 | 270 | 6.75 |
| 14 | GQ8273069 | 22-12-2004 | 285 | 220 | 18.8 | 12.1 | 1.25 | 17.8 | 17.66 | 755 | 2.05 | 0.85 | 6.1 | 25 | 0.23 | 270 | 6.75 |
| 15 | GQ8273069 | 01-04-2008 | 354 | 186 | 26.22 | 15.1 | 15.96 | 21.97 | 21 | 598 | 1.87 | 0.96 | 3.58 | 43.6 | 0.41 | 179 | 7.05 |
| 16 | GQ8273069 | 07-02-2010 | 345 | 170 | 18.27 | 12.18 | 9.25 | 18.25 | 55.08 | 616 | 2 | 1.5 | 3.15 | 40.15 | 0.3 | 170 | 7.04 |
| 17 | GQ8273069 | 01-02-2013 | 345 | 165.2 | 16.32 | 12.61 | 9.25 | 19.25 | 17.66 | 593 | 2 | 2.75 | 4.1 | 40.75 | 0.27 | 150 | 6.5 |
| 18 | GQ8273069 | 01-02-2014 | 340 | 160 | 14.62 | 11.79 | 8.75 | 18.15 | 4.4 | 567 | 3 | 3.1 | 4.4 | 41 | 0.25 | 145 | 6.6 |
| 19 | GQ8273069 | 01-11-2014 | 350 | 156 | 12.18 | 12.83 | 7.25 | 19.75 | 55.08 | 538 | 4 | 3.75 | 4.5 | 42 | 0.27 | 148 | 6.5 |
| 20 | GQ8273069 | 14-11-2015 | 350 | 156 | 12.18 | 12.83 | 7.25 | 19.75 | 21 | 538 | 4 | 3.75 | 4.5 | 42 | 0.27 | 148 | 6.5 |
| 21 | GQ8273069 | 01-04-2016 | 457 | 45 | 27 | 12.42 | 1 | 42 | 55.08 | 510 | 0.5 | 0.5 | 0.1 | 22.1 | 0.3 | 250 | 8.1 |
| 22 | GQ8273069 | 04-04-2018 | 269 | 156 | 13.92 | 11.4 | 9.25 | 16 | 4.40 | 200 | 1 | 0.1 | 0.56 | 41 | 0.08 | 276 | 5.93 |
| 23 | GQ8273069 | 17-09-2018 | 423 | 97.2 | 30.27 | 40.71 | 1.25 | 16 | 64.80 | 750 | 8 | 0.4 | 4.82 | 22.1 | 0.32 | 20 | 5.5 |
| 24 | GQ8273069 | 25-07-2019 | 388 | 61.13 | 35.7 | 21.23 | 1.25 | 37.5 | 40.00 | 230 | 2.05 | 0.9 | 4.8 | 25 | 0.32 | 148 | 6.94 |
| 25 | GQ8273069 | 15-11-2019 | 404 | 213.2 | 18.27 | 21.23 | 9.25 | 41 | 17.59 | 398 | 1 | 0.9 | 0.5 | 42 | 0.45 | 270 | 5.2 |

Note: All units are mg/L except pH (-).

**Table A3 Groundwater Quality of Tangail (Mirzapur)**

| **SL** | **WELL ID** | **DATE** | **TDS** | **CALCIUM** | **MAGNESIUM** | **SODIUM** | **POTASSIUM** | **CHLORIDE** | **CARBONATE** | **BICARBONATE** | **SULPHATE** | **NITRATE** | **IRON** | **SILICA** | **FLUORIDE** | **CARBONDIOXIDE** | **pH** |
| --- | --- | --- | --- | --- | --- | --- | --- | --- | --- | --- | --- | --- | --- | --- | --- | --- | --- |
| 1 | GQ9366051 | 01-03-1985 | 340 | 7 | 4.8 | 62.79 | 3.75 | 26.83 | 23.52 | 98.29 | 93.1 | 7.2 | 1.52 | 85 | 0.47 | 88 | 7.6 |
| 2 | GQ9366051 | 01-03-1986 | 565 | 18.03 | 4.25 | 211.11 | 3.75 | 18.89 | 58.80 | 460.3 | 4.9 | 0.35 | 0.5 | 12.7 | 0.47 | 135 | 7.36 |
| 3 | GQ9366051 | 01-03-1991 | 179 | 23 | 16 | 7 | 6.25 | 12 | 15.00 | 152 | 10 | 7.2 | 13.5 | 27.01 | 0.13 | 136 | 6.4 |
| 4 | GQ9366051 | 01-03-1992 | 195 | 37 | 24 | 12 | 6.1 | 19 | 58.8 | 240 | 16 | 5.6 | 5.7 | 26.5 | 0.35 | 16 | 6.9 |
| 5 | GQ9366051 | 01-03-1993 | 169 | 48 | 72 | 7.8 | 7 | 12 | 15 | 146 | 18 | 1.8 | 3.81 | 1.52 | 0.11 | 16 | 6.63 |
| 6 | GQ9366051 | 12-11-1994 | 150 | 49.7 | 15.4 | 0.46 | 3.75 | 0.7 | 68 | 125 | 18 | 0.3 | 5.5 | 72 | 0.46 | 136 | 7.35 |
| 7 | GQ9366051 | 20-06-1996 | 150 | 22 | 20.95 | 1.82 | 6.25 | 20.8 | 23.52 | 123 | 4 | 1.8 | 9.5 | 32.3 | 0.23 | 84 | 8.37 |
| 8 | GQ9366051 | 15-04-1997 | 170 | 72.8 | 29.06 | 0.78 | 7 | 1.2 | 6.8 | 131 | 19 | 0.7 | 8.5 | 45 | 0.35 | 88 | 6.88 |
| 9 | GQ9366051 | 31-10-1997 | 180 | 82 | 26.36 | 2.6 | 5.68 | 4 | 68 | 193 | 9 | 0.9 | 11.25 | 44.8 | 0.01 | 150 | 9.16 |
| 10 | GQ9366051 | 20-07-1998 | 150 | 32.31 | 15.12 | 12.6 | 2.45 | 5.8 | 6.8 | 178 | 13.08 | 0.2 | 4.43 | 20.29 | 0.4 | 135 | 7.8 |
| 11 | GQ9366051 | 16-12-1998 | 350 | 52 | 15.5 | 0.45 | 3.8 | 0.7 | 80 | 218 | 18.05 | 0.35 | 5.6 | 75 | 0.47 | 135 | 7.35 |
| 12 | GQ9366051 | 15-03-2000 | 230 | 52 | 15.5 | 0.45 | 3.75 | 0.7 | 15 | 220 | 18 | 0.35 | 5.7 | 80 | 0.47 | 135 | 7.35 |
| 13 | GQ9366051 | 19-02-2001 | 350 | 52 | 15.6 | 0.45 | 3.76 | 0.7 | 68 | 220 | 18 | 0.35 | 5.75 | 85 | 0.48 | 130 | 7.4 |
| 14 | GQ9366051 | 29-12-2004 | 350 | 52 | 15.6 | 0.45 | 3.76 | 0.8 | 15 | 225 | 18 | 0.35 | 5.8 | 85 | 0.48 | 130 | 7.4 |
| 15 | GQ9366051 | 01-05-2008 | 201 | 51.2 | 12.69 | 0.08 | 5.68 | 0.14 | 80 | 181 | 0.92 | 2.46 | 16.32 | 72.2 | 0.41 | 119 | 6.72 |
| 16 | GQ9366051 | 23-01-2010 | 205 | 47.2 | 9.01 | 1.13 | 4.5 | 1.75 | 23.52 | 190 | 1 | 2 | 16.75 | 61.42 | 0.35 | 122 | 6.9 |
| 17 | GQ9366051 | 01-02-2013 | 210 | 59.2 | 9.01 | 3.09 | 4.25 | 4.75 | 68 | 204 | 18 | 3.15 | 7 | 58.25 | 0.35 | 85 | 7.6 |
| 18 | GQ9366051 | 01-02-2014 | 220 | 42.8 | 10.47 | 4.42 | 5.75 | 6.8 | 15 | 178 | 6 | 4.86 | 13.75 | 67.5 | 0.35 | 117 | 6.3 |
| 19 | GQ9366051 | 01-11-2014 | 230 | 40.4 | 9.98 | 4.64 | 6.1 | 7.15 | 6.8 | 174 | 7 | 5.2 | 13.5 | 65.7 | 0.39 | 110 | 6.5 |
| 20 | GQ9366051 | 14-11-2015 | 235 | 52.8 | 6.82 | 3.99 | 4.9 | 6.15 | 23.52 | 175 | 18 | 4.8 | 6.6 | 59.25 | 0.4 | 96 | 7.1 |
| 21 | GQ9366051 | 01-04-2016 | 237 | 25 | 15 | 0.1 | 4.25 | 14 | 23.52 | 226 | 18 | 2 | 4.5 | 74.43 | 0.33 | 120 | 8.4 |
| 22 | GQ9366051 | 21-03-2018 | 117.7 | 18.03 | 24 | 1.82 | 5.68 | 18 | 6.80 | 125 | 1 | 0.6 | 7.1 | 59.25 | 0.44 | 96 | 9.74 |
| 23 | GQ9366051 | 29-08-2018 | 154.9 | 49.7 | 15.6 | 12 | 3.8 | 11 | 80.00 | 152 | 6 | 0.5 | 2.54 | 27.01 | 0.11 | 135 | 6.83 |
| 24 | GQ9366051 | 09-06-2019 | 150 | 18.03 | 26.36 | 12.6 | 6.25 | 38 | 68.00 | 156 | 6 | 0.9 | 6.75 | 80 | 0.52 | 85 | 6.96 |
| 25 | GQ9366051 | 07-12-2019 | 130 | 40.4 | 9.01 | 7 | 3.76 | 14 | 80 | 226 | 2 | 0.6 | 3.81 | 27.01 | 0.13 | 135 | 6.96 |

Note: All units are mg/L except pH (-).

**Table A4 Groundwater Quality of Madaripur Sadar**

| **SL** | **WELL ID** | **DATE** | **TDS** | **CALCIUM** | **MAGNESIUM** | **SODIUM** | **POTASSIUM** | **CHLORIDE** | **CARBONATE** | **BICARBONATE** | **SULPHATE** | **NITRATE** | **IRON** | **SILICA** | **FLUORIDE** | **CARBONDIOXIDE** | **pH** |
| --- | --- | --- | --- | --- | --- | --- | --- | --- | --- | --- | --- | --- | --- | --- | --- | --- | --- |
| 1 | GQ5454074 | 01-03-1985 | 1160 | 47.5 | 119.4 | 330.97 | 9.85 | 11.57 | 11.57 | 364.6 | 44.03 | 0.9 | 0.88 | 40.2 | 0.14 | 8.78 | 7.5 |
| 2 | GQ5454074 | 01-03-1986 | 360 | 37.05 | 27.33 | 61.78 | 17 | 93.93 | 11.59 | 191.2 | 4.95 | 4.6 | 1.52 | 40.2 | 0.23 | 6.8 | 7.37 |
| 3 | GQ5454074 | 01-03-1991 | 954 | 34 | 41.4 | 270.19 | 11.1 | 308.1 | 9 | 444.8 | 0.9 | 4.25 | 6.21 | 40 | 0.28 | 13.48 | 9 |
| 4 | GQ5454074 | 01-03-1992 | 1080 | 200.8 | 29 | 357.62 | 10.75 | 83 | 11.39 | 870 | 16 | 0.2 | 1.23 | 17.89 | 0.14 | 74 | 7.2 |
| 5 | GQ5454074 | 01-03-1993 | 931 | 79.6 | 119.4 | 247 | 9.85 | 380 | 23.52 | 265 | 44.03 | 0.9 | 1.33 | 1.22 | 0.15 | 44 | 7.45 |
| 6 | GQ5454074 | 26-12-1994 | 950 | 140 | 27.35 | 258.37 | 16.75 | 397.5 | 3.2 | 287 | 3 | 0.7 | 1.23 | 40.9 | 0.1 | 100 | 7.9 |
| 7 | GQ5454074 | 30-06-1997 | 822 | 99.6 | 16.36 | 367.25 | 15.25 | 565 | 11.39 | 289 | 3 | 2.2 | 0.87 | 47.2 | 0.23 | 74 | 7.46 |
| 8 | GQ5454074 | 07-11-1997 | 960 | 168.8 | 39.55 | 446.88 | 10.75 | 687.5 | 3.2 | 330 | 1 | 0.6 | 168 | 30.9 | 0.15 | 124 | 7.56 |
| 9 | GQ5454074 | 23-07-1998 | 865 | 80.4 | 33.96 | 231.1 | 4.35 | 411 | 64 | 354 | 0.28 | 0.2 | 1.17 | 17.89 | 0.28 | 145 | 8.09 |
| 10 | GQ5454074 | 09-12-1998 | 1070 | 220.4 | 28 | 260 | 17 | 400 | 76 | 840 | 3 | 0.7 | 1.15 | 41 | 0.16 | 144 | 7.67 |
| 11 | GQ5454074 | 10-03-2000 | 950 | 220 | 29 | 260 | 17 | 400 | 3.2 | 841 | 1 | 0.7 | 1.15 | 40 | 0.15 | 145 | 7.7 |
| 12 | GQ5454074 | 03-01-2001 | 950 | 220 | 29 | 260 | 17 | 400 | 11.39 | 841 | 1 | 0.7 | 1.15 | 40 | 0.15 | 145 | 7.8 |
| 13 | GQ5454074 | 26-12-2004 | 950 | 223 | 29.8 | 262 | 17 | 450 | 11.39 | 553 | 16 | 6.2 | 4.24 | 47.2 | 0.15 | 150 | 7.8 |
| 14 | GQ5454074 | 01-04-2008 | 1048 | 200.8 | 22.5 | 357.62 | 16.38 | 517 | 30.6 | 374 | 6 | 1.08 | 0.99 | 44 | 0.19 | 163 | 7.69 |
| 15 | GQ5454074 | 11-02-2010 | 1982 | 170 | 12.18 | 312 | 11.2 | 480 | 64 | 594 | 1 | 2.5 | 0.75 | 45.75 | 0.2 | 171 | 7.7 |
| 16 | GQ5454074 | 01-02-2013 | 1090 | 160 | 12.18 | 302.25 | 10.75 | 465 | 30.6 | 559 | 3 | 4.25 | 0.5 | 40.48 | 0.23 | 155 | 7.5 |
| 17 | GQ5454074 | 01-02-2014 | 1080 | 158 | 10.96 | 302 | 11.1 | 465 | 64 | 547 | 2 | 5.3 | 0.4 | 41.25 | 0.25 | 150 | 7.3 |
| 18 | GQ5454074 | 01-11-2014 | 1070 | 160 | 11.45 | 292.5 | 9.85 | 450 | 11.39 | 553 | 3 | 6.2 | 0.65 | 40.75 | 0.23 | 146 | 7.4 |
| 19 | GQ5454074 | 14-11-2015 | 1070 | 160 | 11.45 | 292.5 | 9.85 | 450 | 30.6 | 553 | 3 | 6.2 | 0.65 | 40.75 | 0.23 | 146 | 7.4 |
| 20 | GQ5454074 | 01-04-2016 | 1749 | 120 | 72 | 301 | 1.5 | 576 | 64 | 870 | 1 | 5 | 0.2 | 40.2 | 0.2 | 150 | 7.3 |
| 21 | GQ5454074 | 04-04-2018 | 642 | 80.4 | 11.45 | 292.5 | 9.85 | 87 | 7.2 | 286 | 1 | 4.6 | 2.3 | 30.9 | 0.18 | 155 | 5.79 |
| 22 | GQ5454074 | 19-09-2018 | 865 | 160 | 11.45 | 260 | 16.38 | 99 | 45.2 | 289 | 9 | 0.8 | 6.21 | 40.2 | 0.14 | 13.48 | 6.2 |
| 23 | GQ5454074 | 09-05-2019 | 1080 | 160 | 12.18 | 231.1 | 9.85 | 162.5 | 72 | 240 | 6 | 0.7 | 4.24 | 40 | 0.01 | 145 | 6.1 |
| 24 | GQ5454074 | 16-11-2019 | 847 | 79.6 | 12.18 | 231.1 | 10.75 | 116 | 23.52 | 240 | 0.9 | 0.7 | 5 | 40 | 0.36 | 145 | 6.1 |

Note: All units are mg/L except pH (-).

**Table A5 Groundwater Quality of Kishoreganj (Bhairab)**

| **SL** | **WELL ID** | **DATE** | **TDS** | **CALCIUM** | **MAGNESIUM** |  | **SODIUM** | **POTASSIUM** | **CHLORIDE** | **CARBONATE** | **BICARBONATE** | **SULPHATE** | **NITRATE** | **IRON** | **SILICA** | **FLUORIDE** | **CARBONDIOXIDE** | **pH** |
| --- | --- | --- | --- | --- | --- | --- | --- | --- | --- | --- | --- | --- | --- | --- | --- | --- | --- | --- |
| 1 | GQ4811048 | 01-03-1985 | 375 | 52.5 | 35.7 |  | 31.51 | 0.55 | 22.86 | 11.39 | 269 | 73.48 | 3.95 | 7.6 | 68.2 | 0.55 | 10.84 | 6.9 |
| 2 | GQ4811048 | 01-03-1986 | 400 | 75.15 | 30.37 |  | 32.03 | 0.55 | 34.79 | 23.52 | 316.8 | 9.76 | 1 | 11.25 | 19.3 | 0.19 | 22.26 | 6.55 |
| 3 | GQ4811048 | 01-03-1990 | 431 | 74 | 21.18 |  | 43.88 | 9.5 | 44.03 | 11.39 | 363.5 | 6.86 | 1.4 | 7.3 | 35 | 0.51 | 22.26 | 8.17 |
| 4 | GQ4811048 | 01-03-1991 | 491 | 18 | 35.4 |  | 112.87 | 0.55 | 79.52 | 30.6 | 286.2 | 36.75 | 0.2 | 6.3 | 64 | 0.53 | 90 | 8.17 |
| 5 | GQ4811048 | 01-03-1993 | 455 | 106.8 | 16.08 |  | 22.1 | 0.55 | 34 | 64 | 252 | 8 | 1.6 | 10.75 | 9.3 | 0.2 | 43 | 7.02 |
| 6 | GQ4811048 | 11-11-1994 | 350 | 102.8 | 16.36 |  | 21.12 | 0.5 | 32.5 | 11.39 | 147 | 11 | 1 | 6 | 54.6 | 0.3 | 160 | 8.13 |
| 7 | GQ4811048 | 04-05-1995 | 300 | 119.6 | 30.76 |  | 20.8 | 9.5 | 32 | 11.39 | 207 | 4 | 1.6 | 3.5 | 31.6 | 0.51 | 114 | 7.48 |
| 8 | GQ4811048 | 17-06-1996 | 290 | 75.2 | 19.29 |  | 23.4 | 7.5 | 36 | 64 | 234 | 5 | 1.4 | 6.3 | 34.6 | 0.12 | 90 | 7.85 |
| 9 | GQ4811048 | 24-04-1997 | 300 | 117.2 | 30.52 |  | 4.22 | 9.75 | 6.5 | 64 | 232 | 1 | 1.3 | 2.82 | 39.9 | 0.24 | 74 | 6.86 |
| 10 | GQ4811048 | 22-12-1997 | 310 | 105.2 | 16.6 |  | 12.94 | 9.5 | 19.9 | 23.52 | 211 | 12 | 1.1 | 3.06 | 40.3 | 0.51 | 128 | 7.76 |
| 11 | GQ4811048 | 18-07-1998 | 310 | 69.78 | 16.73 |  | 14.5 | 3.95 | 2.7 | 76 | 284 | 3.27 | 0.2 | 2.6 | 16.82 | 0.19 | 22.26 | 7.89 |
| 12 | GQ4811048 | 15-03-2000 | 431 | 104 | 16.5 |  | 20.8 | 0.55 | 32.7 | 30.6 | 386 | 10.5 | 1.05 | 6 | 60 | 0.3 | 165 | 8 |
| 13 | GQ4811048 | 18-02-2001 | 500 | 105 | 16 |  | 21 | 0.55 | 32.7 | 3.2 | 385 | 10 | 1 | 6 | 62 | 0.3 | 165 | 7.9 |
| 14 | GQ4811048 | 04-01-2005 | 510 | 110 | 16.5 |  | 23 | 0.55 | 32.8 | 3.2 | 380 | 10 | 1 | 6 | 62 | 0.3 | 165 | 7.9 |
| 15 | GQ4811048 | 01-05-2008 | 402 | 120.4 | 20.62 |  | 12 | 1.98 | 18.68 | 64 | 349 | 9.47 | 1.08 | 5.21 | 64 | 0.51 | 139 | 7.81 |
| 16 | GQ4811048 | 24-01-2010 | 405 | 117.6 | 11.2 |  | 9.1 | 2 | 14 | 11.39 | 402 | 9 | 1.8 | 5.1 | 57 | 0.37 | 143.1 | 7.7 |
| 17 | GQ4811048 | 01-02-2013 | 380 | 107.2 | 9.74 |  | 7.8 | 4.1 | 12 | 30.6 | 361 | 12 | 3.15 | 5.15 | 52 | 0.53 | 130 | 6.55 |
| 18 | GQ4811048 | 01-02-2014 | 370 | 101.2 | 9.01 |  | 7.47 | 4.75 | 11.5 | 11.39 | 337 | 14 | 3.75 | 5.2 | 51.5 | 0.55 | 135 | 6.3 |
| 19 | GQ4811048 | 01-11-2014 | 385 | 104 | 9.74 |  | 6.98 | 4.1 | 10.75 | 76 | 345 | 12 | 3.95 | 5.1 | 50.5 | 5.1 | 142 | 6.1 |
| 20 | GQ4811048 | 14-11-2015 | 385 | 104 | 9.74 |  | 6.98 | 4.1 | 10.75 | 76 | 345 | 12 | 3.95 | 5.1 | 50.5 | 0.52 | 142 | 6.1 |
| 21 | GQ4811048 | 01-04-2016 | 380 | 110 | 19.2 |  | 19.2 | 0.85 | 25 | 11.39 | 380 | 10 | 2.15 | 4.85 | 68.2 | 0.36 | 150 | 7.2 |
| 22 | GQ4811048 | 26-03-2018 | 118.3 | 101.2 | 9.74 |  | 20.8 | 4.1 | 25 | 3.2 | 212 | 1 | 0.9 | 1.77 | 57 | 0.36 | 150 | 5.65 |
| 23 | GQ4811048 | 02-09-2018 | 301 | 110 | 16.6 |  | 7.8 | 0.55 | 19 | 64 | 385 | 12 | 1 | 6.34 | 57 | 0.55 | 142 | 6.9 |
| 24 | GQ4811048 | 11-06-2019 | 380 | 110 | 35.7 |  | 43.88 | 9.75 | 77.5 | 76 | 248 | 2 | 1.6 | 6.82 | 9.3 | 0.2 | 165 | 7.2 |
| 25 | GQ4811048 | 09-12-2019 | 321 | 69.78 | 16.73 |  | 21 | 4.1 | 68 | 23.52 | 234 | 9.76 | 0.7 | 6.2 | 9.3 | 0.2 | 160 | 7.16 |

Note: All units are mg/L except pH (-).

**Table A6 Comparison of Man Kendal Test and Modified Man Kendal Test Result**

|  | **TDS** | **CALCIUM** | **MAGNESIUM** | **SODIUM** | **POTASSIUM** | **CHLORIDE** | **BICARBONATE** | **SULPHATE** | **NITRATE** | **IRON** | **SILICA** | **FLUORIDE** | **CARBONDIOXIDE** | **pH** | **CARBONATE** |
| --- | --- | --- | --- | --- | --- | --- | --- | --- | --- | --- | --- | --- | --- | --- | --- |
| Mann Kandal (Average) | 0.11 | 0.77 | -1.07 | -0.53 | 0.19 | 0.44 | 0.85 | -0.34 | 0.61 | -0.79 | 1.34 | 0.64 | 1.87 | -1.73 | 0.52 |
| Modified Mann Kendal (Average) | 0.14 | 0.66 | -1.03 | -0.42 | 0.14 | 0.25 | 0.74 | -0.37 | 0.55 | -0.84 | 1.42 | 0.61 | 1.63 | -1.84 | 0.68 |
| Difference (%) | 0.03 | 0.11 | 0.05 | -0.11 | 0.04 | 0.18 | 0.11 | 0.03 | 0.06 | 0.05 | 0.08 | 0.03 | 0.25 | 0.11 | 0.15 |

Note: All units are mg/L except pH (-).

**Table A7 Groundwater Quality of Rajbari Sadar**

| **SL** | **WELL ID** | **DATETIME** | **TDS** | **CALCIUM** | **MAGNESIUM** | **SODIUM** | **POTASSIUM** | **CHLORIDE** | **CARBONATE** | **BICARBONATE** | **SULPHATE** | **NITRATE** | **IRON** | **SILICA** | **FLUORIDE** | **CARBONDIOXIDE** | **PH** |
| --- | --- | --- | --- | --- | --- | --- | --- | --- | --- | --- | --- | --- | --- | --- | --- | --- | --- |
| 1 | GQ8276070 | 01-03-1985 | 510 | 51 | 55.8 | 63.71 | 12.5 | 17.89 | 2.03 | 510.5 | 49.05 | 0.5 | 1.92 | 32 | 0.14 | 33.01 | 7.5 |
| 2 | GQ8276070 | 01-03-1986 | 318 | 14 | 39.49 | 66.01 | 12.55 | 17.19 | 29.35 | 203.2 | 19.64 | 4.2 | 0.11 | 24 | 0.25 | 5 | 7.19 |
| 4 | GQ8276070 | 01-03-1991 | 517 | 13.51 | 39 | 130.84 | 12.55 | 24.83 | 8 | 528.8 | 9.8 | 0.4 | 1.45 | 41.2 | 0.14 | 4.45 | 9.5 |
| 6 | GQ8276070 | 01-03-1993 | 429 | 142 | 213 | 5.2 | 3 | 8 | 8 | 401 | 5 | 1.1 | 1.44 | 14.4 | 0.05 | 76 | 7.08 |
| 7 | GQ8276070 | 23-12-1994 | 320 | 128 | 16.6 | 10.98 | 12.5 | 16.9 | 2.03 | 290 | 5 | 0.5 | 1.38 | 38.8 | 0.15 | 40 | 7.6 |
| 8 | GQ8276070 | 29-04-1995 | 330 | 119.6 | 16.85 | 11.38 | 3.75 | 17.5 | 2.8 | 274 | 1 | 1.4 | 5.25 | 26 | 0.18 | 70 | 7.71 |
| 9 | GQ8276070 | 28-06-1997 | 300 | 144.8 | 34.92 | 13.33 | 7.5 | 20.5 | 36 | 399 | 4 | 0.2 | 1.38 | 39.2 | 0.16 | 70 | 7.18 |
| 10 | GQ8276070 | 06-11-1997 | 380 | 152 | 18.31 | 113.75 | 7.5 | 175 | 2.03 | 433 | 1.51 | 0.5 | 4.65 | 52.4 | 0.16 | 134 | 6.48 |
| 11 | GQ8276070 | 09-11-1997 | 360 | 117.6 | 19.28 | 7.02 | 12.5 | 10.8 | 29.35 | 340 | 45 | 1.1 | 2.05 | 36.3 | 0.1 | 54 | 8.65 |
| 12 | GQ8276070 | 22-07-1998 | 530 | 97.71 | 36.12 | 24.3 | 5.55 | 10.6 | 48.4 | 481 | 1.51 | 0.7 | 4.43 | 19.1 | 0.14 | 54 | 7.9 |
| 13 | GQ8276070 | 07-12-1998 | 510 | 104 | 17 | 11.05 | 12.55 | 17 | 2.8 | 422 | 56 | 0.6 | 1.45 | 39 | 0.15 | 780 | 7.18 |
| 14 | GQ8276070 | 03-10-2000 | 400 | 104 | 17 | 11.05 | 12.5 | 17 | 8 | 420 | 37 | 0.6 | 1.45 | 38 | 0.15 | 790 | 7.2 |
| 15 | GQ8276070 | 02-01-2001 | 420 | 105 | 16 | 11 | 12.5 | 17 | 29.35 | 420 | 19.64 | 0.6 | 1.45 | 40 | 0.15 | 790 | 7.25 |
| 16 | GQ8276070 | 22-12-2004 | 425 | 105 | 16 | 11 | 12.5 | 18 | 2.8 | 425 | 49.05 | 0.6 | 1.5 | 40 | 0.15 | 790 | 7.25 |
| 17 | GQ8276070 | 01-04-2008 | 482 | 123.6 | 26.28 | 13.22 | 9.97 | 21 | 29.35 | 399 | 9.8 | 0.39 | 1.56 | 41.2 | 0.21 | 697 | 7.17 |
| 18 | GQ8276070 | 07-02-2010 | 450 | 115.6 | 17.2 | 14.95 | 9.25 | 23 | 29.35 | 449 | 1 | 1 | 1.15 | 35 | 0.2 | 720.5 | 7.23 |
| 19 | GQ8276070 | 01-02-2013 | 460 | 110 | 14.61 | 13 | 8.25 | 20 | 48.4 | 413 | 3 | 3.25 | 1.4 | 32 | 0.25 | 510 | 7.1 |
| 20 | GQ8276070 | 01-02-2014 | 470 | 108 | 13.4 | 15.11 | 9.15 | 23.25 | 36 | 401 | 4 | 3.2 | 1.5 | 31 | 0.27 | 490 | 6.9 |
| 21 | GQ8276070 | 01-11-2014 | 450 | 99.2 | 12.67 | 16.25 | 8.25 | 25 | 48.4 | 367 | 5 | 4.2 | 1.3 | 32 | 0.25 | 598 | 6.8 |
| 22 | GQ8276070 | 14-11-2015 | 450 | 99.2 | 12.67 | 16.25 | 8.25 | 25 | 29.35 | 367 | 5 | 4.2 | 1.3 | 32 | 0.25 | 598 | 6.8 |
| 23 | GQ8276070 | 01-04-2016 | 629 | 75 | 45 | 13.35 | 3 | 40 | 2.8 | 414 | 1.51 | 0.5 | 0.2 | 39.2 | 0.1 | 810 | 7.5 |
| 24 | GQ8276070 | 04-04-2018 | 428 | 99.2 | 16 | 13.33 | 7.5 | 23 | 2.8 | 118 | 45 | 3.6 | 0.38 | 19.1 | 0.06 | 134 | 5.74 |
| 25 | GQ8276070 | 25-07-2018 | 450 | 97.71 | 55.8 | 7.02 | 12.5 | 35 | 36 | 158 | 4 | 0.4 | 3.5 | 31 | 0.05 | 780 | 6.8 |
| 26 | GQ8276070 | 17-09-2018 | 563 | 128 | 12.67 | 15.11 | 7.5 | 21 | 48.4 | 274 | 56 | 0.1 | 6.22 | 38 | 0.1 | 790 | 5.9 |
| 27 | GQ8276070 | 15-11-2019 | 544 | 152 | 39 | 13 | 12.5 | 35 | 48.4 | 340 | 37 | 0.9 | 5.57 | 32 | 0.35 | 790 | 6.1 |

Note: All units are mg/L except pH (-).

**Table A8 Groundwater Quality of Narsingdi Sadar**

| **SL** | **WELL ID** | **DATETIME** | **TDS** | **CALCIUM** | **MAGNESIUM** | **SODIUM** | **POTASSIUM** | **CHLORIDE** | **CARBONATE** | **BICARBONATE** | **SULPHATE** | **NITRATE** | **IRON** | **SILICA** | **FLUORIDE** | **CARBONDIOXIDE** | **PH** |
| --- | --- | --- | --- | --- | --- | --- | --- | --- | --- | --- | --- | --- | --- | --- | --- | --- | --- |
| 1 | GQ6860053 | 01-03-1985 | 455 | 35.5 | 23.7 | 101.89 | 4 | 77.53 | 14.58 | 263 | 58.69 | 1.4 | 3.6 | 0.11 | 75 | 11.79 | 6.8 |
| 2 | GQ6860053 | 01-03-1986 | 405 | 72.64 | 38.57 | 25.15 | 3.07 | 38.76 | 11.73 | 358.6 | 4.86 | 0.22 | 11.2 | 0.4 | 17.4 | 8 | 8.1 |
| 4 | GQ6860053 | 01-03-1991 | 355 | 28.5 | 19.8 | 70.48 | 3.07 | 28.82 | 15.33 | 295.5 | 22.06 | 3.25 | 8.61 | 0.4 | 70 | 44 | 6.71 |
| 6 | GQ6860053 | 01-03-1993 | 274 | 79.2 | 118.8 | 20.1 | 4 | 34 | 13.27 | 174 | 5 | 0.2 | 5.38 | 0.76 | 0.64 | 44 | 7.14 |
| 7 | GQ6860053 | 05-12-1994 | 290 | 107.66 | 24.9 | 21.12 | 4 | 32.5 | 15.33 | 180 | 1 | 0.2 | 7 | 0.43 | 69.5 | 106 | 7.66 |
| 8 | GQ6860053 | 17-06-1996 | 330 | 52 | 35.41 | 3.55 | 4.5 | 47 | 13.27 | 204 | 1 | 1.1 | 4.3 | 0.38 | 21.9 | 114 | 8.04 |
| 9 | GQ6860053 | 24-04-1997 | 330 | 108.4 | 30.77 | 18.53 | 8.75 | 28.5 | 15.33 | 220 | 2 | 1 | 8.75 | 0.61 | 39.5 | 108 | 7.07 |
| 10 | GQ6860053 | 22-12-1997 | 530 | 118 | 28.08 | 81.25 | 4 | 125 | 13.27 | 207 | 1 | 1.9 | 4.8 | 0.11 | 23.7 | 104 | 8.63 |
| 11 | GQ6860053 | 18-07-1998 | 405 | 57.1 | 33.81 | 17.9 | 3.07 | 64.2 | 11.73 | 290 | 0.03 | 0.2 | 5.16 | 0.62 | 16.22 | 122 | 8.1 |
| 12 | GQ6860053 | 17-12-1998 | 290 | 110 | 24.92 | 8.15 | 4 | 12.55 | 15.33 | 464 | 1.02 | 0.22 | 7 | 0.43 | 70 | 110 | 7.7 |
| 13 | GQ6860053 | 15-03-2000 | 500 | 110 | 25 | 8.15 | 4 | 12.55 | 15.33 | 465 | 1 | 0.2 | 7 | 0.42 | 70 | 120 | 7.65 |
| 14 | GQ6860053 | 20-02-2001 | 500 | 110 | 25.05 | 8.2 | 4 | 12.5 | 13.27 | 465 | 1 | 0.2 | 7 | 0.4 | 75 | 120 | 7.6 |
| 15 | GQ6860053 | 27-12-2004 | 490 | 112 | 25.55 | 8.2 | 4 | 12.6 | 15.33 | 467 | 1 | 0.2 | 7 | 0.4 | 75 | 120 | 7.6 |
| 16 | GQ6860053 | 01-05-2008 | 298 | 113.6 | 29.52 | 12.68 | 5.19 | 20.08 | 14.58 | 167 | 0.89 | 0.81 | 7.61 | 0.68 | 67.1 | 119 | 7.1 |
| 17 | GQ6860053 | 04-02-2010 | 270 | 98 | 13.39 | 11.53 | 4.5 | 17.75 | 14.58 | 366 | 2 | 1.75 | 2.5 | 0.58 | 58 | 122 | 7.2 |
| 18 | GQ6860053 | 01-02-2013 | 260 | 87.2 | 9.74 | 11.7 | 9.74 | 18 | 14.58 | 309 | 5 | 3.8 | 2.5 | 0.55 | 68.15 | 120 | 7.3 |
| 19 | GQ6860053 | 01-02-2014 | 250 | 83.2 | 9.01 | 11.05 | 8.75 | 17 | 14.58 | 301 | 4 | 3.25 | 2.75 | 0.52 | 67.5 | 115 | 7.6 |
| 20 | GQ6860053 | 01-11-2014 | 270 | 80.8 | 8.76 | 10.4 | 7.3 | 16 | 11.73 | 311 | 5 | 4.1 | 2.25 | 0.5 | 65.15 | 110 | 7.75 |
| 21 | GQ6860053 | 14-11-2015 | 270 | 80.8 | 8.76 | 10.4 | 7.3 | 16 | 98 | 311 | 5 | 4.1 | 2.25 | 0.5 | 65.15 | 110 | 7.75 |
| 22 | GQ6860053 | 01-04-2016 | 310 | 106.7 | 24.3 | 12.79 | 4.87 | 12 | 15.33 | 490 | 3.35 | 2.25 | 7.14 | 0.6 | 75.2 | 130 | 7.3 |
| 23 | GQ6860053 | 26-03-2018 | 149 | 80.8 | 29.52 | 8.15 | 3.07 | 23 | 9.2 | 160 | 1 | 1 | 2.58 | 0.51 | 68.15 | 120 | 5.77 |
| 24 | GQ6860053 | 02-09-2018 | 382 | 57.1 | 33.81 | 12.68 | 3.07 | 15 | 98 | 301 | 2 | 1.8 | 3.5 | 0.23 | 39.5 | 120 | 6.56 |
| 25 | GQ6860053 | 09-12-2019 | 394 | 113.6 | 24.3 | 70.48 | 8.75 | 77 | 11.73 | 309 | 5 | 1.4 | 3.47 | 0.76 | 39.5 | 119 | 6.71 |

Note: All units are mg/L except pH (-).

**Table A9 Groundwater Quality of Munshiganj**

| **SL** | **WELL ID** | **DATETIME** | **TDS** | **CALCIUM** | **MAGNESIUM** | **SODIUM** | **POTASSIUM** | **CHLORIDE** | **CARBONATE** | **BICARBONATE** | **SULPHATE** | **NITRATE** | **IRON** | **SILICA** | **FLUORIDE** | **CARBONDIOXIDE** | **PH** |
| --- | --- | --- | --- | --- | --- | --- | --- | --- | --- | --- | --- | --- | --- | --- | --- | --- | --- |
| 1 | GQ5984101 | 01-03-1985 | 551 | 59.95 | 43.56 | 129.91 | 5.33 | 107.3 | 58.8 | 424.4 | 6 | 2.1 | 13.2 | 21.25 | 0.74 | 124 | 7.9 |
| 2 | GQ5984101 | 01-03-1986 | 462 | 13.5 | 25.21 | 130.25 | 5.25 | 107.3 | 64.68 | 128.5 | 8 | 2.8 | 1 | 16 | 0.42 | 80 | 6.95 |
| 3 | GQ5984101 | 01-03-1993 | 780 | 113.6 | 170.4 | 45.5 | 5.75 | 70 | 65.53 | 450 | 7 | 0.8 | 3.93 | 1.1 | 0.2 | 80 | 7.2 |
| 4 | GQ5984101 | 10-01-1995 | 630 | 122 | 32.72 | 47.12 | 3.79 | 72.5 | 65.53 | 442 | 0.79 | 2.8 | 9.25 | 24 | 0.38 | 188 | 7.79 |
| 5 | GQ5984101 | 23-04-1995 | 650 | 83.6 | 9.52 | 12 | 5 | 107.5 | 58.8 | 427 | 2 | 0.9 | 8.25 | 24.2 | 0.42 | 116 | 7.66 |
| 6 | GQ5984101 | 15-06-1996 | 630 | 61.6 | 29.3 | 46.8 | 6.25 | 72 | 6.8 | 447 | 1 | 0.9 | 6.25 | 21.1 | 0.14 | 14 | 7.86 |
| 7 | GQ5984101 | 03-04-1997 | 610 | 129.2 | 30.28 | 117 | 13 | 180 | 6.8 | 414 | 3 | 2.5 | 8.25 | 28.5 | 0.42 | 66 | 6.95 |
| 8 | GQ5984101 | 05-11-1997 | 630 | 116.4 | 26.12 | 203.13 | 13 | 312.5 | 58.8 | 358 | 2 | 3.9 | 9.2 | 21.7 | 0.35 | 30 | 7.96 |
| 9 | GQ5984101 | 21-07-1998 | 630 | 65.01 | 31.27 | 132.8 | 5.33 | 93.8 | 64.68 | 579 | 0.79 | 3.8 | 4.43 | 12.65 | 0.31 | 125 | 8.16 |
| 10 | GQ5984101 | 17-12-1998 | 620 | 124 | 32.75 | 195 | 3.8 | 300 | 6.8 | 545 | 7 | 3.1 | 9.25 | 22 | 0.38 | 112 | 7.8 |
| 11 | GQ5984101 | 16-03-2000 | 626 | 124 | 33 | 195 | 3.85 | 300 | 6.8 | 550 | 2 | 3.1 | 9.2 | 22 | 0.2 | 112 | 7.8 |
| 12 | GQ5984101 | 23-02-2001 | 750 | 124 | 33 | 195 | 3.85 | 300 | 6.8 | 550 | 2 | 3.1 | 9.2 | 22 | 0.2 | 150 | 7.82 |
| 13 | GQ5984101 | 26-12-2004 | 760 | 124 | 33 | 195 | 3.85 | 305 | 65.53 | 555 | 2 | 3.1 | 9.2 | 22 | 0.31 | 150 | 7.82 |
| 14 | GQ5984101 | 01-06-2008 | 626 | 119.6 | 36.34 | 145.1 | 3.31 | 219.5 | 68 | 513 | 3 | 3.08 | 8.28 | 22 | 0.1 | 109 | 7.25 |
| 15 | GQ5984101 | 05-02-2010 | 630 | 114.8 | 2.21 | 117 | 4 | 180 | 68 | 449 | 0.79 | 3.25 | 7.9 | 25 | 0.35 | 115 | 7.5 |
| 16 | GQ5984101 | 01-02-2013 | 630 | 110 | 17.78 | 115.7 | 5.25 | 178 | 64.68 | 420 | 6 | 4.15 | 8 | 20.3 | 0.2 | 120 | 7.25 |
| 17 | GQ5984101 | 01-02-2014 | 620 | 106.8 | 12.91 | 118.3 | 5.75 | 182 | 68 | 384 | 8 | 4.95 | 7.8 | 21.25 | 0.22 | 115 | 7.3 |
| 18 | GQ5984101 | 01-11-2014 | 635 | 108.8 | 13.4 | 113.75 | 4.9 | 175 | 65.53 | 394 | 7 | 4.1 | 0.15 | 23.1 | 0.2 | 113 | 7.18 |
| 19 | GQ5984101 | 14-11-2015 | 635 | 108.8 | 13.4 | 113.75 | 4.9 | 175 | 64.68 | 394 | 7 | 4.1 | 0.15 | 23.1 | 0.2 | 113 | 7.18 |
| 20 | GQ5984101 | 01-04-2016 | 736 | 50 | 30 | 200 | 4.8 | 72 | 64.68 | 550 | 2 | 1.5 | 1.2 | 27 | 0.2 | 124 | 7.9 |
| 21 | GQ5984101 | 24-03-2018 | 435 | 106.8 | 32.75 | 117 | 5.33 | 21 | 6.8 | 360 | 2 | 2.1 | 3.39 | 21.1 | 0.17 | 109 | 6.34 |
| 22 | GQ5984101 | 09-09-2018 | 574 | 83.6 | 43.56 | 113.75 | 5.25 | 25 | 68 | 427 | 0.79 | 2.2 | 3.5 | 22 | 0.61 | 124 | 7.66 |
| 23 | GQ5984101 | 09-12-2019 | 569 | 61.6 | 32.75 | 118.3 | 5.75 | 81 | 64.68 | 394 | 3 | 0.5 | 3.5 | 21.7 | 0.74 | 112 | 6.9 |

Note: All units are mg/L except pH (-).

**Table A10 Groundwater Quality of Motijheel**

| **SL** | **WELL ID** | **DATETIME** | **TDS** | **CALCIUM** | **MAGNESIUM** | **SODIUM** | **POTASSIUM** | **CHLORIDE** | **CARBONATE** | **BICARBONATE** | **SULPHATE** | **NITRATE** | **IRON** | **SILICA** | **FLUORIDE** | **CARBONDIOXIDE** | **PH** |
| --- | --- | --- | --- | --- | --- | --- | --- | --- | --- | --- | --- | --- | --- | --- | --- | --- | --- |
| 1 | GQ2654054 | 01-03-1985 | 230 | 45.65 | 16.17 | 13.54 | 5.25 | 42.74 | 17.64 | 107.6 | 31.85 | 0.6 | 1.4 | 50.8 | 0.3 | 4 | 6.29 |
| 2 | GQ2654054 | 01-03-1986 | 727 | 21.53 | 21.87 | 21.1 | 1.6 | 44.77 | 5.78 | 143.4 | 24.5 | 3.5 | 1.45 | 36.4 | 0.04 | 4.99 | 7.6 |
| 3 | GQ2654054 | 01-03-1993 | 160 | 43.2 | 64.8 | 3.9 | 1.65 | 6 | 76 | 66 | 1 | 3.7 | 0.02 | 1.28 | 0.1 | 42 | 6.21 |
| 4 | GQ2654054 | 31-12-1994 | 180 | 42 | 8.54 | 14.4 | 1.5 | 21.7 | 76 | 106 | 1 | 2.5 | 0.03 | 69.5 | 0.26 | 28 | 6.61 |
| 5 | GQ2654054 | 13-06-1996 | 170 | 11.2 | 10.26 | 13.46 | 8.5 | 20.7 | 4.4 | 94 | 5 | 3.5 | 0.03 | 50.8 | 0.18 | 46 | 8.24 |
| 6 | GQ2654054 | 03-05-1997 | 170 | 46 | 11.23 | 10.4 | 5.5 | 16 | 4.4 | 54 | 14 | 2.6 | 0.34 | 77.1 | 0.3 | 68 | 6.29 |
| 7 | GQ2654054 | 05-11-1997 | 166 | 5.36 | 16.63 | 2.94 | 2.63 | 4.52 | 5.78 | 65 | 2.48 | 0.65 | 0.09 | 1.2 | 0.28 | 47 | 6.28 |
| 8 | GQ2654054 | 16-07-1998 | 230 | 26.25 | 10.06 | 21.1 | 1.36 | 30 | 4.4 | 120 | 4.5 | 2.3 | 0.07 | 32.25 | 0.22 | 4 | 8.1 |
| 9 | GQ2654054 | 17-12-1998 | 250 | 50 | 8.55 | 2.99 | 1.6 | 4.6 | 76 | 196 | 1.48 | 0.9 | 0.08 | 11 | 0.15 | 42 | 7.6 |
| 10 | GQ2654054 | 16-03-2000 | 230 | 50 | 8.5 | 3.02 | 1.65 | 4.65 | 76 | 196 | 1.48 | 0.9 | 0.09 | 12 | 0.15 | 40 | 7.8 |
| 11 | GQ2654054 | 22-02-2001 | 250 | 50 | 8.6 | 3 | 1.65 | 4.6 | 17.64 | 195 | 1.5 | 0.9 | 0.09 | 12 | 0.22 | 40 | 7.8 |
| 12 | GQ2654054 | 25-12-2004 | 250 | 50 | 8.6 | 3 | 1.65 | 4.65 | 17.64 | 190 | 1.5 | 0.9 | 0.09 | 12 | 0.22 | 40 | 7.8 |
| 13 | GQ2654054 | 01-06-2008 | 222 | 49.6 | 11.24 | 4.18 | 3.11 | 6.03 | 4.4 | 149 | 1.29 | 0.78 | 0.09 | 52.35 | 0.04 | 55 | 7.56 |
| 14 | GQ2654054 | 04-02-2010 | 220 | 51.2 | 7.8 | 4.71 | 2.65 | 7.25 | 4.4 | 194 | 4 | 1.75 | 0.05 | 48 | 0.1 | 60 | 7.5 |
| 15 | GQ2654054 | 01-02-2013 | 230 | 52.4 | 8.28 | 5.85 | 4.15 | 9 | 5.78 | 196 | 5 | 3.75 | 0.1 | 48.75 | 0.08 | 65 | 7.5 |
| 16 | GQ2654054 | 01-02-2014 | 240 | 54 | 9.74 | 5.53 | 5.35 | 8.5 | 17.64 | 215 | 6 | 4.12 | 0.15 | 49 | 0.1 | 70 | 7.8 |
| 17 | GQ2654054 | 01-11-2014 | 235 | 51.2 | 8.77 | 6.17 | 5.25 | 9.5 | 5.78 | 193 | 7 | 5.1 | 0.17 | 48.25 | 0.15 | 75 | 7.6 |
| 18 | GQ2654054 | 14-11-2015 | 235 | 51.2 | 8.77 | 6.17 | 5.25 | 9.5 | 76 | 193 | 7 | 5.1 | 0.17 | 48.25 | 0.15 | 75 | 7.6 |
| 19 | GQ2654054 | 01-04-2016 | 290 | 50 | 9.23 | 3.5 | 2.63 | 6.35 | 4.4 | 189 | 2.26 | 2 | 0.08 | 15.1 | 0.01 | 38 | 7.2 |
| 20 | GQ2654054 | 29-03-2018 | 127.3 | 26.25 | 21.87 | 3 | 5.5 | 15 | 4.4 | 160 | 5 | 0.3 | 0.18 | 12 | 0.03 | 40 | 5.79 |
| 21 | GQ2654054 | 09-09-2018 | 185 | 49.6 | 8.6 | 2.94 | 5.25 | 14 | 76 | 193 | 1 | 0.6 | 6.22 | 48.25 | 0.28 | 65 | 6.81 |
| 22 | GQ2654054 | 10-12-2019 | 225 | 49.6 | 8.77 | 3.02 | 5.5 | 54 | 4.4 | 193 | 14 | 1.3 | 5.66 | 1.2 | 0.63 | 75 | 6.4 |
| 23 | GQ5984101 | 09-12-2019 | 569 | 61.6 | 32.75 | 118.3 | 5.75 | 81 | 64.68 | 394 | 3 | 0.5 | 3.5 | 21.7 | 0.74 | 112 | 6.9 |

Note: All units are mg/L except pH (-).

**Table A11 Groundwater Quality of Tangail (Madhupur)**

| **SL** | **WELL ID** | **DATETIME** | **TDS** | **CALCIUM** | **MAGNESIUM** | **SODIUM** | **POTASSIUM** | **CHLORIDE** | **CARBONATE** | **BICARBONATE** | **SULPHATE** | **NITRATE** | **IRON** | **SILICA** | **FLUORIDE** | **CARBONDIOXIDE** | **PH** |
| --- | --- | --- | --- | --- | --- | --- | --- | --- | --- | --- | --- | --- | --- | --- | --- | --- | --- |
| 1 | GQ9357049 | 01-03-1985 | 191 | 23.1 | 10.23 | 23.41 | 2.75 | 8.15 | 5.36 | 125.2 | 34.3 | 1.1 | 20.4 | 29.4 | 0.25 | 2 | 6.9 |
| 2 | GQ9357049 | 01-03-1986 | 201 | 19.53 | 15.8 | 29.78 | 6.1 | 11.91 | 5.75 | 101.6 | 29.4 | 1.1 | 2.78 | 29.4 | 0.24 | 2 | 6.95 |
| 3 | GQ9357049 | 01-03-1992 | 108 | 14 | 11 | 6 | 5.8 | 10 | 4 | 112 | 10 | 5.6 | 14.65 | 30.1 | 0.24 | 4 | 6.9 |
| 4 | GQ9357049 | 01-03-1993 | 120 | 31.2 | 46.8 | 6.5 | 2.8 | 10 | 7 | 94 | 1 | 0.9 | 14.65 | 1.02 | 0.55 | 40 | 6.06 |
| 5 | GQ9357049 | 12-11-1994 | 110 | 34.4 | 12.7 | 0.52 | 2.75 | 0.8 | 7 | 120 | 1 | 1.1 | 25.75 | 65.5 | 0.24 | 178 | 6.93 |
| 6 | GQ9357049 | 20-06-1996 | 90 | 15.6 | 9.52 | 0.2 | 6 | 0.3 | 5.36 | 87 | 1 | 0.2 | 0.46 | 41.5 | 0.16 | 92 | 7.98 |
| 7 | GQ9357049 | 15-04-1997 | 90 | 28.4 | 9.77 | 4.55 | 7 | 7 | 52 | 203 | 1 | 0.9 | 1.76 | 71.6 | 0.37 | 124 | 6.35 |
| 8 | GQ9357049 | 31-10-1997 | 100 | 44.4 | 17.33 | 0.85 | 2.8 | 1.3 | 5.6 | 86 | 1 | 0.8 | 2.25 | 34.5 | 0.4 | 166 | 7.43 |
| 9 | GQ9357049 | 20-07-1998 | 201 | 16.61 | 4.8 | 16.3 | 1.28 | 1.3 | 5.6 | 118 | 0.93 | 0.2 | 2.84 | 28.4 | 0.23 | 92 | 7.87 |
| 10 | GQ9357049 | 16-12-1998 | 191 | 36 | 12.75 | 0.52 | 2.8 | 0.8 | 7 | 176 | 1 | 1.1 | 25.75 | 67 | 0.25 | 180 | 6.95 |
| 11 | GQ9357049 | 15-03-2000 | 134 | 36 | 12.8 | 0.52 | 2.8 | 0.8 | 5.6 | 180 | 0.93 | 1.1 | 5.75 | 68 | 0.25 | 180 | 6.9 |
| 12 | GQ9357049 | 19-02-2001 | 400 | 35 | 13 | 0.5 | 2.85 | 0.8 | 7 | 180 | 29.4 | 1.1 | 25.8 | 70 | 0.25 | 185 | 6.9 |
| 13 | GQ9357049 | 01-01-2005 | 400 | 35 | 13 | 0.5 | 2.85 | 0.8 | 5.6 | 180 | 10 | 1.1 | 25.9 | 70 | 0.25 | 185 | 6.9 |
| 14 | GQ9357049 | 22-01-2010 | 125 | 34.8 | 11.44 | 3.08 | 2.8 | 4.25 | 80 | 162 | 1 | 1.5 | 21.25 | 71 | 0.55 | 145 | 6.75 |
| 15 | GQ9357049 | 01-02-2013 | 140 | 41.2 | 11.45 | 4.38 | 5.15 | 6.75 | 52 | 181 | 4 | 3.75 | 20.8 | 67 | 0.37 | 110 | 6.4 |
| 16 | GQ9357049 | 01-02-2014 | 135 | 42.8 | 11.69 | 5.03 | 6.1 | 7.75 | 5.36 | 186 | 5 | 4.12 | 20.15 | 68.25 | 0.35 | 120 | 6.8 |
| 17 | GQ9357049 | 01-11-2014 | 145 | 44.4 | 11.93 | 5.29 | 5.8 | 8.15 | 5.6 | 192 | 4 | 4.75 | 20.5 | 67.25 | 0.37 | 127 | 6.9 |
| 18 | GQ9357049 | 14-11-2015 | 145 | 44.4 | 11.93 | 5.29 | 5.8 | 8.15 | 80 | 192 | 4 | 4.75 | 20.5 | 67.25 | 0.37 | 127 | 6.9 |
| 19 | GQ9357049 | 01-04-2016 | 134 | 15 | 9 | 0.8 | 5.8 | 18 | 5.75 | 201 | 1 | 5.6 | 0.1 | 70 | 0.3 | 185 | 8.1 |
| 20 | GQ9357049 | 21-03-2018 | 123 | 42.8 | 11 | 5.29 | 5.8 | 19 | 5.6 | 142 | 1 | 9.6 | 5.83 | 29.4 | 0.17 | 185 | 2.86 |
| 21 | GQ9357049 | 29-08-2018 | 86.7 | 15.6 | 15.8 | 6 | 6.1 | 13 | 52 | 158 | 1 | 0.7 | 2.09 | 67.25 | 0.35 | 92 | 6.3 |
| 22 | GQ9357049 | 09-06-2019 | 191 | 15.6 | 15.8 | 5.03 | 5.8 | 32.5 | 80 | 158 | 4 | 0.7 | 3.5 | 65.5 | 0.4 | 180 | 6.35 |
| 23 | GQ9357049 | 07-12-2019 | 130 | 42.8 | 11 | 0.5 | 5.8 | 23 | 4 | 180 | 1 | 0.6 | 3.51 | 68.25 | 0.89 | 185 | 5.6 |

Note: All units are mg/L except pH (-).

**Table A12 Groundwater Quality of Tangail Sadar**

| **SL** | **WELL ID** | **DATETIME** | **TDS** | **CALCIUM** | **MAGNESIUM** | **SODIUM** | **POTASSIUM** | **CHLORIDE** | **CARBONATE** | **BICARBONATE** | **SULPHATE** | **NITRATE** | **IRON** | **SILICA** | **FLUORIDE** | **CARBONDIOXIDE** | **PH** |
| --- | --- | --- | --- | --- | --- | --- | --- | --- | --- | --- | --- | --- | --- | --- | --- | --- | --- |
| 1 | GQ9395050 | 01-03-1985 | 232 | 35.2 | 17.49 | 19.13 | 2.1 | 20.82 | 5.88 | 149.4 | 39.2 | 1.5 | 9.76 | 34.5 | 0.32 | 0.7 | 7.8 |
| 2 | GQ9395050 | 01-03-1986 | 240 | 34.56 | 19.74 | 21.71 | 2 | 14.41 | 6.72 | 161.4 | 34.25 | 0.99 | 8 | 20.6 | 0.32 | 23.1 | 8.04 |
| 3 | GQ9395050 | 01-03-1991 | 412 | 41 | 20.1 | 87.6 | 2.1 | 34.79 | 11.18 | 289.3 | 85.75 | 0.9 | 18.7 | 19.06 | 0.45 | 12.97 | 7.8 |
| 4 | GQ9395050 | 01-03-1992 | 192 | 42 | 15 | 17 | 2.1 | 27 | 12 | 156 | 12 | 5.6 | 8 | 19 | 0.3 | 119 | 6.6 |
| 5 | GQ9395050 | 01-03-1993 | 202 | 62.4 | 93.6 | 16.9 | 3.01 | 26 | 12 | 158 | 1 | 2.2 | 9.15 | 1.13 | 0.19 | 40 | 6.59 |
| 6 | GQ9395050 | 12-11-1994 | 220 | 75.6 | 16.11 | 7.93 | 2 | 12.2 | 11.18 | 183 | 1 | 1.5 | 18.5 | 79 | 0.31 | 120 | 7.1 |
| 7 | GQ9395050 | 27-04-1995 | 230 | 85.2 | 22.95 | 7.47 | 4 | 11.5 | 64 | 310 | 4.1 | 1.2 | 11.25 | 34.3 | 0.23 | 124 | 6.66 |
| 8 | GQ9395050 | 20-06-1996 | 220 | 49.2 | 12.94 | 12.42 | 5.5 | 19.1 | 40 | 185 | 34.25 | 1.7 | 10.2 | 32.4 | 0.19 | 92 | 8.04 |
| 9 | GQ9395050 | 15-04-1997 | 270 | 46.4 | 12.21 | 8.06 | 10.5 | 12.4 | 64 | 208 | 34 | 0.9 | 15.75 | 45.6 | 0.28 | 126 | 6.97 |
| 10 | GQ9395050 | 31-10-1997 | 230 | 78.4 | 20.12 | 8 | 2.1 | 12.3 | 4.8 | 199 | 2 | 0.9 | 16.75 | 34.5 | 0.28 | 112 | 9.9 |
| 11 | GQ9395050 | 20-07-1998 | 192 | 48.07 | 12.18 | 9.1 | 3.01 | 20 | 40 | 192 | 0.23 | 0.2 | 7.33 | 19.06 | 0.27 | 112 | 7.74 |
| 12 | GQ9395050 | 16-12-1998 | 232 | 76.8 | 16.11 | 8.78 | 2 | 12.75 | 6.72 | 317 | 1 | 1.5 | 18.6 | 82 | 0.32 | 125 | 7.15 |
| 13 | GQ9395050 | 15-03-2000 | 232 | 76.8 | 16 | 8.9 | 2.1 | 12.75 | 64 | 310 | 1 | 1.5 | 18.7 | 80 | 0.32 | 125 | 7.15 |
| 14 | GQ9395050 | 19-02-2001 | 450 | 77 | 16 | 8.85 | 2.1 | 12.8 | 6.72 | 310 | 1 | 1.5 | 18.75 | 80 | 0.32 | 120 | 7.1 |
| 15 | GQ9395050 | 01-01-2005 | 455 | 77 | 16 | 8.85 | 2.1 | 12.7 | 6.72 | 315 | 1 | 1.5 | 18.8 | 80 | 0.32 | 120 | 7.1 |
| 16 | GQ9395050 | 01-05-2008 | 232 | 83.6 | 14.5 | 7.57 | 4.46 | 12.08 | 11.18 | 297 | 1 | 0.99 | 17.78 | 77 | 0.37 | 119 | 7.01 |
| 17 | GQ9395050 | 01-05-2008 | 136 | 32.4 | 11.23 | 3.31 | 3.49 | 2.65 | 40 | 179 | 39.2 | 1.03 | 23.47 | 71 | 0.31 | 144 | 6.8 |
| 18 | GQ9395050 | 22-01-2010 | 220 | 79.2 | 11.44 | 6.98 | 33.75 | 10.75 | 5.88 | 298 | 2 | 1.5 | 16.5 | 65.69 | 0.25 | 121 | 6.92 |
| 19 | GQ9395050 | 01-02-2013 | 210 | 76 | 9.74 | 3.09 | 9.74 | 4.75 | 6.72 | 272 | 4.1 | 4.75 | 16 | 72 | 0.3 | 120 | 7.4 |
| 20 | GQ9395050 | 01-02-2014 | 218 | 74.4 | 8.28 | 3.08 | 8.75 | 4.75 | 11.18 | 269 | 4 | 4.85 | 15.25 | 73 | 0.45 | 115 | 7.5 |
| 21 | GQ9395050 | 01-11-2014 | 220 | 71.2 | 7.24 | 3.99 | 7.75 | 6.15 | 5.88 | 251 | 5 | 4.85 | 14.9 | 72.25 | 0.47 | 119 | 7.8 |
| 22 | GQ9395050 | 14-11-2015 | 220 | 71.2 | 7.24 | 3.99 | 7.75 | 6.15 | 11.18 | 251 | 5 | 4.85 | 14.9 | 72.25 | 0.47 | 119 | 7.8 |
| 23 | GQ9395050 | 01-04-2016 | 565 | 60 | 36 | 7.89 | 4.5 | 80 | 12 | 302 | 10 | 10 | 0.3 | 80.6 | 0.23 | 130 | 7.9 |
| 24 | GQ9395050 | 21-03-2018 | 232 | 35.2 | 7.24 | 21.71 | 2.1 | 132 | 4.8 | 272 | 34 | 4.9 | 0.61 | 79 | 0.17 | 144 | 7.1 |
| 25 | GQ9395050 | 29-08-2018 | 514 | 41 | 16.11 | 8 | 4.46 | 27 | 40 | 146 | 34 | 1.8 | 0.21 | 32.4 | 0.16 | 40 | 6.29 |
| 26 | GQ9395050 | 09-06-2019 | 270 | 79.2 | 16 | 16.9 | 10.5 | 167.5 | 64 | 146 | 68 | 4.2 | 3.5 | 82 | 0.48 | 120 | 6.59 |
| 27 | GQ9395050 | 07-12-2019 | 452.2 | 76.8 | 20.1 | 19.13 | 33.75 | 140 | 59.27 | 310 | 3.5 | 4 | 2.45 | 82 | 0.28 | 130 | 6.52 |

Note: All units are mg/L except pH (-).

**Table A13 Groundwater Quality of Tangail Textile Mill**

| **SL** | **WELL ID** | **DATETIME** | **TDS** | **CALCIUM** | **MAGNESIUM** | **SODIUM** | **POTASSIUM** | **CHLORIDE** | **CARBONATE** | **BICARBONATE** | **SULPHATE** | **NITRATE** | **IRON** | **SILICA** | **FLUORIDE** | **CARBONDIOXIDE** | **PH** |
| --- | --- | --- | --- | --- | --- | --- | --- | --- | --- | --- | --- | --- | --- | --- | --- | --- | --- |
| 1 | GQ9366103 | 20-06-1996 | 150 | 21.2 | 0.3 | 1.43 | 10 | 2.2 | 76 | 153 | 1 | 0.1 | 5.75 | 42.7 | 0.22 | 72 | 8.56 |
| 2 | GQ9366103 | 15-04-1997 | 160 | 54.8 | 19.29 | 0.13 | 5.25 | 0.2 | 6.8 | 144 | 20 | 2.25 | 8 | 62.8 | 0.4 | 130 | 6.86 |
| 3 | GQ9366103 | 31-10-1997 | 170 | 55.2 | 16.11 | 2.68 | 5.25 | 2.5 | 6.8 | 166 | 16 | 2.6 | 10 | 20.7 | 0.3 | 144 | 7.42 |
| 4 | GQ9366103 | 20-07-1998 | 170 | 28.76 | 10.96 | 25.1 | 0.85 | 2.5 | 72 | 215 | 1.31 | 0.2 | 1.48 | 25.12 | 0.34 | 110 | 7.9 |
| 5 | GQ9366103 | 16-12-1998 | 235 | 46 | 11.75 | 0.07 | 2.6 | 0.12 | 76 | 198 | 1.02 | 2.65 | 19.3 | 70 | 0.25 | 110 | 7.05 |
| 6 | GQ9366103 | 15-03-2000 | 240 | 46 | 12 | 0.07 | 2.7 | 0.12 | 6.8 | 200 | 1 | 2.65 | 19.2 | 70 | 0.25 | 110 | 7.05 |
| 7 | GQ9366103 | 19-02-2001 | 350 | 45 | 12 | 0.07 | 2.7 | 0.15 | 72 | 220 | 1 | 2.6 | 19.5 | 75 | 0.25 | 110 | 7.1 |
| 8 | GQ9366103 | 29-12-2004 | 350 | 45 | 12 | 0.07 | 2.7 | 0.15 | 76 | 220 | 1 | 2.6 | 19.6 | 75 | 0.25 | 110 | 7.1 |
| 9 | GQ9366103 | 01-05-2008 | 233 | 58.4 | 19.84 | 0.49 | 4.48 | 0.79 | 72 | 216 | 19.1 | 0.51 | 6.3 | 70.5 | 0.53 | 98 | 7.08 |
| 10 | GQ9366103 | 23-01-2010 | 240 | 64 | 12.18 | 0.65 | 4 | 1 | 6.8 | 235 | 18 | 1 | 6.1 | 64.5 | 0.3 | 99.5 | 7.1 |
| 11 | GQ9366103 | 01-02-2013 | 225 | 47.2 | 11.45 | 3.35 | 5.25 | 5.15 | 72 | 196 | 5 | 4.75 | 13.25 | 67.45 | 0.38 | 120 | 6.55 |
| 12 | GQ9366103 | 01-02-2014 | 227 | 55.2 | 7.79 | 3.73 | 4.8 | 5.75 | 6.8 | 188 | 16 | 4.25 | 6.75 | 57.75 | 0.38 | 90 | 7.25 |
| 13 | GQ9366103 | 01-11-2014 | 235 | 52.8 | 6.82 | 3.99 | 4.9 | 6.15 | 76 | 175 | 18 | 4.8 | 6.6 | 59.25 | 0.4 | 96 | 7.1 |
| 14 | GQ9366103 | 14-11-2015 | 230 | 40.4 | 9.98 | 4.64 | 6.1 | 7.15 | 76 | 174 | 7 | 5.2 | 13.5 | 65.7 | 0.39 | 110 | 6.5 |
| 15 | GQ9366103 | 01-04-2016 | 200 | 59.2 | 9.01 | 0.5 | 3.75 | 2.75 | 76 | 260 | 20 | 2.25 | 6 | 85 | 0.57 | 136 | 7.1 |
| 16 | GQ9366103 | 21-03-2018 | 109.5 | 58.4 | 19.84 | 3.35 | 4.9 | 23 | 6.8 | 188 | 3 | 15.7 | 6.57 | 75 | 0.45 | 110 | 5.72 |
| 17 | GQ9366103 | 29-08-2018 | 337 | 55.2 | 11.75 | 3.35 | 5.25 | 18 | 76 | 144 | 34 | 0.4 | 3.37 | 70.5 | 0.09 | 96 | 6.75 |
| 18 | GQ9366103 | 09-06-2019 | 337 | 55.2 | 11.45 | 25.1 | 4.9 | 107.5 | 72 | 190 | 87 | 0.9 | 3.5 | 57.75 | 0.04 | 96 | 6.79 |
| 19 | GQ9366103 | 07-12-2019 | 75.22 | 64 | 19.84 | 4.64 | 5.25 | 47 | 72 | 215 | 33 | 0.6 | 6.41 | 75 | 0.55 | 99.5 | 6.79 |

Note: All units are mg/L except pH (-).

**Table A14 Groundwater Quality of Faridpur Sadar**

| **SL** | **WELL ID** | **DATETIME** | **TDS** | **CALCIUM** | **MAGNESIUM** | **SODIUM** | **POTASSIUM** | **CHLORIDE** | **CARBONATE** | **BICARBONATE** | **SULPHATE** | **NITRATE** | **IRON** | **SILICA** | **FLUORIDE** | **CARBONDIOXIDE** | **PH** |
| --- | --- | --- | --- | --- | --- | --- | --- | --- | --- | --- | --- | --- | --- | --- | --- | --- | --- |
| 1 | GQ2947071 | 01-03-1985 | 540 | 83 | 57.6 | 46 | 9.75 | 24.85 | 11.48 | 582.8 | 15 | 8.2 | 0.64 | 24 | 0.31 | 39.89 | 6.9 |
| 2 | GQ2947071 | 01-03-1986 | 376 | 34 | 36.45 | 59.44 | 15 | 30.81 | 29.42 | 263 | 24.54 | 0.1 | 0.63 | 24 | 0.43 | 2.89 | 7.5 |
| 3 | GQ2947071 | 01-03-1991 | 717 | 25 | 44.7 | 91.67 | 14.5 | 30.87 | 58.8 | 718.6 | 29.4 | 2 | 1.01 | 17.36 | 0.25 | 48.44 | 8 |
| 4 | GQ2947071 | 01-03-1992 | 460 | 156.4 | 20.17 | 91.67 | 4.54 | 72 | 29.42 | 613 | 12 | 6.15 | 10.05 | 41.15 | 0.25 | 48.44 | 7.2 |
| 5 | GQ2947071 | 01-03-1993 | 522 | 151.2 | 22.68 | 80.13 | 15 | 20 | 29.42 | 452 | 12 | 1.1 | 8 | 1.21 | 0.25 | 124 | 7.7 |
| 6 | GQ2947071 | 24-12-1994 | 470 | 165.6 | 33.2 | 8.45 | 14.5 | 13 | 58.8 | 421 | 15 | 1.9 | 9.75 | 37.6 | 0.6 | 70 | 7.84 |
| 7 | GQ2947071 | 29-06-1997 | 390 | 156.4 | 26.13 | 11.77 | 9 | 18.1 | 84 | 302 | 1 | 5.85 | 0.17 | 37 | 0.25 | 108 | 6.38 |
| 8 | GQ2947071 | 07-11-1997 | 480 | 198.4 | 53.96 | 11.31 | 15 | 17.4 | 29.42 | 441 | 2 | 2.8 | 9.4 | 36.8 | 0.14 | 280 | 8.84 |
| 9 | GQ2947071 | 23-07-1998 | 440 | 105.9 | 37.22 | 29.5 | 4.54 | 14 | 6.4 | 552 | 0.21 | 2.4 | 5.67 | 17.36 | 0.15 | 246 | 8.36 |
| 10 | GQ2947071 | 07-12-1998 | 376 | 140 | 33 | 8.45 | 15 | 13 | 11.48 | 613 | 3 | 2 | 10.04 | 38 | 0.24 | 244 | 7.48 |
| 11 | GQ2947071 | 03-10-2000 | 450 | 144 | 33.1 | 8.45 | 15 | 13 | 11.48 | 610 | 7 | 2 | 10.05 | 38 | 0.24 | 250 | 7.48 |
| 12 | GQ2947071 | 02-01-2001 | 455 | 144 | 33.1 | 8.45 | 15 | 13 | 11.48 | 610 | 12 | 2 | 10.05 | 40 | 0.24 | 13 | 7.5 |
| 13 | GQ2947071 | 23-12-2004 | 450 | 145 | 33.2 | 8.55 | 15 | 13 | 84 | 612 | 7 | 2 | 10.1 | 40 | 0.24 | 250 | 7.5 |
| 14 | GQ2947071 | 01-04-2008 | 468 | 139.6 | 29.2 | 9.84 | 16.52 | 14.96 | 6.4 | 557 | 6 | 2.03 | 10.21 | 38 | 0.31 | 216 | 7.26 |
| 15 | GQ2947071 | 07-02-2010 | 460 | 130 | 18.26 | 11.8 | 10.25 | 18.25 | 58.8 | 498 | 1 | 2.75 | 9.15 | 34 | 0.25 | 220 | 6.98 |
| 16 | GQ2947071 | 01-02-2013 | 450 | 133.2 | 19.98 | 11.79 | 11.25 | 18.15 | 6.4 | 513 | 3 | 6.15 | 8.25 | 38 | 0.27 | 210 | 6.75 |
| 17 | GQ2947071 | 01-02-2014 | 440 | 135.2 | 20.71 | 12.83 | 10.75 | 19.75 | 6.4 | 518 | 5 | 7.1 | 8.5 | 39 | 0.28 | 200 | 7.08 |
| 18 | GQ2947071 | 01-11-2014 | 430 | 138 | 20.17 | 13.09 | 9.75 | 20.15 | 58.8 | 521 | 7 | 8.2 | 8.15 | 41.15 | 0.25 | 117 | 6.9 |
| 19 | GQ2947071 | 14-11-2015 | 430 | 138 | 20.17 | 13.09 | 9.75 | 20.15 | 58.8 | 521 | 7 | 8.2 | 8.15 | 41.15 | 0.25 | 117 | 6.9 |
| 20 | GQ2947071 | 01-04-2016 | 836 | 85 | 51 | 10.2 | 4 | 11.6 | 84 | 480 | 0.21 | 0.5 | 0.2 | 31 | 0.25 | 246 | 7.5 |
| 21 | GQ2947071 | 04-04-2018 | 315 | 105.9 | 53.96 | 8.45 | 15 | 18 | 6.4 | 613 | 2 | 0.1 | 1.01 | 41.15 | 12 | 48.44 | 3 |
| 22 | GQ2947071 | 17-09-2018 | 360 | 83 | 51 | 12.83 | 15 | 20 | 58.8 | 613 | 9 | 0.5 | 4.07 | 36.8 | 0.43 | 280 | 5.4 |
| 23 | GQ2947071 | 09-05-2019 | 540 | 165.6 | 20.17 | 29.5 | 9 | 87.5 | 84 | 250 | 6 | 1.1 | 6.68 | 41.15 | 0.3 | 48.44 | 6.38 |
| 24 | GQ2947071 | 15-11-2019 | 352 | 133.2 | 20.17 | 12.83 | 9 | 54 | 11.48 | 421 | 1 | 2.5 | 0.5 | 40 | 0.27 | 246 | 5.3 |

Note: All units are mg/L except pH (-).

**Table A15 Groundwater Quality of Gazipur**

| **SL** | **WELL ID** | **DATETIME** | **TDS** | **CALCIUM** | **MAGNESIUM** | **SODIUM** | **POTASSIUM** | **CHLORIDE** | **CARBONATE** | **BICARBONATE** | **SULPHATE** | **NITRATE** | **IRON** | **SILICA** | **FLUORIDE** | **CARBONDIOXIDE** | **PH** |
| --- | --- | --- | --- | --- | --- | --- | --- | --- | --- | --- | --- | --- | --- | --- | --- | --- | --- |
| 1 | GQ3386052 | 01-03-1985 | 205 | 16.5 | 8.1 | 45.5 | 3 | 8.96 | 6 | 113.5 | 5.635 | 2.5 | 1.4 | 14.3 | 0.34 | 34.7 | 7.82 |
| 2 | GQ3386052 | 01-03-1986 | 208 | 21.04 | 12.15 | 38.92 | 3.75 | 11.95 | 5.76 | 119.5 | 19.6 | 5 | 0.47 | 32.55 | 0.37 | 88.9 | 6.95 |
| 3 | GQ3386052 | 01-03-1992 | 100 | 34 | 12.91 | 16 | 3.75 | 4.5 | 5.95 | 165 | 12 | 0.7 | 4.1 | 35.2 | 0.55 | 55 | 7.2 |
| 4 | GQ3386052 | 01-03-1993 | 320 | 20.8 | 31.2 | 17.9 | 0.7 | 12 | 5.67 | 83 | 3 | 1.4 | 2.32 | 14.3 | 0.57 | 50 | 7.79 |
| 5 | GQ3386052 | 11-11-1994 | 210 | 32 | 10.7 | 2.01 | 3 | 3.1 | 5.2 | 81 | 6 | 0.7 | 5 | 70.9 | 0.35 | 46 | 7.66 |
| 6 | GQ3386052 | 14-06-1996 | 100 | 15.2 | 3.42 | 3.9 | 4.75 | 6 | 6 | 98 | 1 | 0.2 | 3.12 | 35.2 | 0.01 | 90 | 7.86 |
| 7 | GQ3386052 | 13-04-1997 | 130 | 79.2 | 21.49 | 2.15 | 8.5 | 3.3 | 5.95 | 56 | 5.635 | 0.9 | 1.61 | 59.2 | 0.45 | 56 | 6.95 |
| 8 | GQ3386052 | 29-10-1997 | 170 | 50 | 18.31 | 2.08 | 3.75 | 3.2 | 5.2 | 94 | 2 | 0.1 | 0.82 | 49.8 | 0.35 | 20 | 7.96 |
| 9 | GQ3386052 | 17-07-1998 | 100 | 19.75 | 6.77 | 17.9 | 0.7 | 3.8 | 5.67 | 134 | 0.83 | 0.2 | 1.62 | 27.28 | 0.37 | 48 | 8.16 |
| 10 | GQ3386052 | 16-12-1998 | 244 | 34 | 10.7 | 2.04 | 3.05 | 3.15 | 5.76 | 160 | 6 | 0.7 | 5.05 | 72 | 0.35 | 48 | 7.8 |
| 11 | GQ3386052 | 15-03-2000 | 300 | 34 | 11 | 2.04 | 3 | 3.15 | 6 | 160 | 5.635 | 0.7 | 5 | 75 | 0.35 | 50 | 7.8 |
| 12 | GQ3386052 | 19-02-2001 | 300 | 35 | 11 | 2.05 | 3 | 3.15 | 5.76 | 165 | 4 | 0.7 | 5 | 78 | 0.35 | 55 | 7.82 |
| 13 | GQ3386052 | 28-12-2004 | 295 | 35 | 11 | 2.05 | 3 | 3.15 | 5.2 | 160 | 5.635 | 0.7 | 5 | 78 | 0.35 | 55 | 7.82 |
| 14 | GQ3386052 | 01-05-2008 | 308 | 31.2 | 16.87 | 2.1 | 2.69 | 3.19 | 5.2 | 143 | 8 | 0.69 | 4.61 | 69.4 | 0.34 | 51 | 7.25 |
| 15 | GQ3386052 | 23-01-2010 | 315 | 33.2 | 11.45 | 2.92 | 1.75 | 4.5 | 5.95 | 153 | 2 | 2.5 | 4.1 | 65.22 | 0.5 | 50.5 | 7.5 |
| 16 | GQ3386052 | 01-02-2013 | 320 | 38 | 12.18 | 3.41 | 2.3 | 5.25 | 5.76 | 168 | 4 | 4.25 | 4.5 | 62.45 | 0.55 | 70 | 7.25 |
| 17 | GQ3386052 | 01-02-2014 | 315 | 40 | 12.66 | 4 | 3.15 | 6.15 | 6 | 176 | 6 | 4.1 | 4.8 | 60.75 | 0.58 | 75 | 7.3 |
| 18 | GQ3386052 | 01-11-2014 | 310 | 42.8 | 12.91 | 4.38 | 3.75 | 6.75 | 5.95 | 183 | 8 | 5 | 4.3 | 61.7 | 0.57 | 73 | 7.18 |
| 19 | GQ3386052 | 14-11-2015 | 310 | 42.8 | 12.91 | 4.38 | 3.75 | 6.75 | 5.67 | 183 | 8 | 5 | 4.3 | 61.7 | 0.57 | 73 | 7.18 |
| 20 | GQ3386052 | 01-04-2016 | 134 | 15 | 9 | 2.5 | 4.75 | 12 | 6 | 170 | 1 | 1.2 | 0.1 | 77.85 | 0.4 | 50 | 7.9 |
| 21 | GQ3386052 | 10-04-2018 | 244 | 15.2 | 11 | 2.08 | 4.75 | 13 | 4.4 | 168 | 2 | 1.2 | 0.17 | 77.85 | 0.44 | 50 | 6.34 |
| 22 | GQ3386052 | 08-09-2018 | 109.5 | 15.2 | 6.77 | 3.41 | 3.15 | 12 | 5.2 | 83 | 1 | 0.4 | 6.06 | 75 | 0.4 | 70 | 7.96 |
| 23 | GQ3386052 | 11-06-2019 | 320 | 15 | 21.49 | 16 | 1.75 | 35 | 8.4 | 140 | 3 | 1.2 | 5.26 | 72 | 0.56 | 48 | 6.9 |
| 24 | GQ3386052 | 10-12-2019 | 235 | 42.8 | 12.91 | 2.04 | 8.5 | 11 | 5.67 | 113.5 | 1 | 0.8 | 4.19 | 75 | 0.44 | 88.9 | 7.18 |

Note: All units are mg/L except pH (-).

**Table A16 Groundwater Quality of Gopalganj Kashiani**

| **SL** | **WELL ID** | **DATETIME** | **TDS** | **CALCIUM** | **MAGNESIUM** | **SODIUM** | **POTASSIUM** | **CHLORIDE** | **CARBONATE** | **BICARBONATE** | **SULPHATE** | **NITRATE** | **IRON** | **SILICA** | **FLUORIDE** | **CARBONDIOXIDE** | **PH** |
| --- | --- | --- | --- | --- | --- | --- | --- | --- | --- | --- | --- | --- | --- | --- | --- | --- | --- |
| 1 | GQ3543072 | 01-03-1985 | 998 | 104 | 57 | 69.92 | 12.8 | 18.8 | 6 | 660.5 | 7.84 | 5 | 2.72 | 16.5 | 0.4 | 42.79 | 6.8 |
| 2 | GQ3543072 | 01-03-1986 | 291 | 309.9 | 39.79 | 17.97 | 5.75 | 17.09 | 29.48 | 209.2 | 4.39 | 0.9 | 15.5 | 36.85 | 0.32 | 302 | 7.12 |
| 3 | GQ3543072 | 01-03-1991 | 712 | 215 | 42.3 | 19.33 | 12.8 | 87.47 | 10.67 | 608.9 | 2.45 | 0.6 | 10.8 | 37.3 | 0.37 | 109.8 | 8.5 |
| 4 | GQ3543072 | 01-03-1993 | 549 | 185.2 | 27.78 | 1.3 | 4.66 | 20 | 8.24 | 510 | 1.2 | 5 | 20 | 11.1 | 0.02 | 226 | 7.04 |
| 5 | GQ3543072 | 23-12-1994 | 550 | 203.2 | 28.81 | 21.6125 | 12.75 | 33.25 | 9 | 498 | 1 | 1.7 | 17.5 | 35.4 | 0.37 | 280 | 6.93 |
| 6 | GQ3543072 | 29-06-1997 | 501 | 213.6 | 26.37 | 26.33 | 8.5 | 40.5 | 5.2 | 494 | 1 | 1 | 10.25 | 40.2 | 0.23 | 184 | 7.09 |
| 7 | GQ3543072 | 06-11-1997 | 660 | 243.6 | 46.88 | 45.5 | 8.75 | 70 | 9 | 571 | 1 | 2.9 | 27 | 24.1 | 0.45 | 166 | 8.95 |
| 8 | GQ3543072 | 23-07-1998 | 780 | 149.6 | 45.11 | 26.9 | 4.66 | 27.3 | 9 | 567 | 0.35 | 1.4 | 10.61 | 16.76 | 0.02 | 250 | 7.93 |
| 9 | GQ3543072 | 08-12-1998 | 780 | 204 | 28.9 | 20.8 | 12.8 | 32 | 5.2 | 801 | 1 | 1.7 | 12 | 35 | 0.15 | 290 | 7 |
| 10 | GQ3543072 | 03-10-2000 | 720 | 200 | 29 | 20.8 | 12.8 | 32 | 10.67 | 800 | 1 | 1.75 | 12 | 65 | 0.29 | 36 | 7 |
| 11 | GQ3543072 | 03-01-2001 | 850 | 150 | 40 | 25 | 5 | 25 | 6 | 570 | 0.5 | 1.5 | 10.5 | 16.5 | 0.02 | 250 | 7.2 |
| 12 | GQ3543072 | 25-12-2004 | 860 | 152 | 40.5 | 25.8 | 5 | 25 | 8.24 | 565 | 0.5 | 1.5 | 10.6 | 16.5 | 0.08 | 250 | 7.2 |
| 13 | GQ3543072 | 01-04-2008 | 784 | 204.8 | 29.54 | 25.32 | 11.92 | 40.01 | 9 | 756 | 0.99 | 1.58 | 11.52 | 33 | 0.08 | 254 | 6.98 |
| 14 | GQ3543072 | 09-02-2010 | 780 | 160 | 14.61 | 18.03 | 8.75 | 27.75 | 10.67 | 568 | 2 | 2.75 | 11.15 | 34 | 0.15 | 240 | 7 |
| 15 | GQ3543072 | 01-02-2013 | 730 | 156 | 10.96 | 10.88 | 5.75 | 16.75 | 6 | 527 | 4 | 5.25 | 10.1 | 36.85 | 0.35 | 235 | 7.05 |
| 16 | GQ3543072 | 01-02-2014 | 720 | 157.2 | 11.45 | 9.58 | 4.3 | 14.75 | 10.67 | 528 | 6 | 4.75 | 10.75 | 36.25 | 0.37 | 230 | 7.2 |
| 17 | GQ3543072 | 01-11-2014 | 710 | 125.4 | 9.5 | 9.87 | 4.75 | 15.15 | 29.48 | 507 | 5 | 5 | 10.8 | 37.25 | 0.32 | 227 | 7.3 |
| 18 | GQ3543072 | 14-11-2015 | 710 | 152.4 | 9.5 | 9.87 | 5.75 | 15.15 | 8.24 | 507 | 5 | 5 | 10.8 | 37.25 | 0.32 | 227 | 7.3 |
| 19 | GQ3543072 | 01-04-2016 | 716 | 100 | 60 | 20.32 | 10 | 24 | 29.48 | 850 | 1.2 | 1 | 7.5 | 37.3 | 0.01 | 305 | 7.4 |
| 20 | GQ3543072 | 05-04-2018 | 359 | 156 | 45.11 | 25.8 | 4.66 | 18 | 9 | 498 | 1 | 0.5 | 4.63 | 40.2 | 0.28 | 226 | 5.98 |
| 21 | GQ3543072 | 19-09-2018 | 495 | 149.6 | 14.61 | 26.9 | 10 | 14 | 5.2 | 498 | 9 | 0.6 | 3.5 | 36.85 | 0.45 | 166 | 6.1 |
| 22 | GQ3543072 | 25-07-2019 | 359 | 100 | 10.96 | 19.33 | 12.8 | 35 | 8.24 | 318 | 5 | 1 | 5.8 | 36.85 | 0.4 | 109.8 | 6.93 |
| 23 | GQ3543072 | 16-11-2019 | 493 | 150 | 45.11 | 26.9 | 4.66 | 17 | 6 | 570 | 1 | 0.9 | 6.39 | 24.1 | 0.34 | 166 | 5.8 |

Note: All units are mg/L except pH (-).

**Table A17 Groundwater Quality of Gopalganj Sadar**

| **SL** | **WELL ID** | **DATETIME** | **TDS** | **CALCIUM** | **MAGNESIUM** | **SODIUM** | **POTASSIUM** | **CHLORIDE** | **CARBONATE** | **BICARBONATE** | **SULPHATE** | **NITRATE** | **IRON** | **SILICA** | **FLUORIDE** | **CARBONDIOXIDE** | **PH** |
| --- | --- | --- | --- | --- | --- | --- | --- | --- | --- | --- | --- | --- | --- | --- | --- | --- | --- |
| 1 | GQ3532073 | 01-03-1985 | 515 | 51 | 12.91 | 78.89 | 14 | 52.68 | 12 | 386.1 | 73.47 | 4.5 | 10.88 | 32 | 0.23 | 21.09 | 7.1 |
| 2 | GQ3532073 | 01-03-1986 | 294 | 23.02 | 30.07 | 38.62 | 14.05 | 28.31 | 17.68 | 185.3 | 19.62 | 3.8 | 18.75 | 32 | 0.23 | 5 | 7.21 |
| 3 | GQ3532073 | 01-03-1990 | 387 | 31 | 18 | 87.71 | 14 | 20.48 | 50.3 | 267.3 | 9.8 | 0.09 | 16 | 22.52 | 0.23 | 17.97 | 8.72 |
| 4 | GQ3532073 | 01-03-1991 | 750 | 26 | 39.3 | 21.142 | 14 | 52.68 | 4.4 | 706.1 | 31.85 | 0.09 | 10.21 | 20 | 0.23 | 17.97 | 8.5 |
| 5 | GQ3532073 | 01-03-1992 | 400 | 100 | 12.91 | 30.87 | 6.5 | 28 | 76 | 267.3 | 24 | 4 | 9.75 | 4.7 | 0.27 | 158 | 7.2 |
| 6 | GQ3532073 | 01-03-1993 | 511 | 100 | 15 | 36.4 | 14 | 56 | 76 | 392 | 3.5 | 7.3 | 27.25 | 12.3 | 0.23 | 194 | 7.32 |
| 7 | GQ3532073 | 24-12-1994 | 480 | 132 | 10.01 | 22.75 | 14 | 35 | 76 | 401 | 24 | 0.1 | 10.25 | 31.4 | 0.05 | 190 | 7.33 |
| 8 | GQ3532073 | 29-04-1995 | 500 | 158 | 24.42 | 9.75 | 6.25 | 15 | 17.64 | 397 | 5 | 1.1 | 13.25 | 27 | 0.22 | 130 | 7.37 |
| 9 | GQ3532073 | 30-06-1997 | 388 | 152.2 | 19.29 | 28.93 | 12.5 | 44.5 | 76 | 347 | 1 | 2.2 | 10.5 | 32.9 | 0.25 | 198 | 7.07 |
| 10 | GQ3532073 | 06-11-1997 | 510 | 168.8 | 29.05 | 9.8 | 6.25 | 15 | 5.78 | 416 | 3.5 | 2.5 | 10.25 | 4.7 | 0.05 | 158 | 7.55 |
| 11 | GQ3532073 | 23-07-1998 | 810 | 107.7 | 23.4 | 45 | 4.1 | 28.4 | 5.78 | 527 | 3.5 | 2.2 | 6.92 | 11.67 | 0.24 | 190 | 8.19 |
| 12 | GQ3532073 | 08-12-1998 | 800 | 139.6 | 10 | 24.7 | 14 | 38 | 4.4 | 496 | 24 | 0.1 | 10.5 | 31 | 0.23 | 204 | 7.4 |
| 13 | GQ3532073 | 03-10-2000 | 400 | 140 | 10 | 24.7 | 14 | 38 | 76 | 500 | 19.62 | 0.1 | 10.6 | 32 | 0.23 | 205 | 7.4 |
| 14 | GQ3532073 | 03-01-2001 | 900 | 140 | 10 | 24.7 | 14 | 38 | 4.4 | 500 | 10 | 0.1 | 10.6 | 20 | 0.23 | 230 | 7.6 |
| 15 | GQ3532073 | 26-12-2004 | 920 | 142 | 10.5 | 24.7 | 14 | 38 | 5.78 | 505 | 24 | 0.1 | 10.6 | 20 | 0.23 | 230 | 7.6 |
| 16 | GQ3532073 | 01-04-2008 | 701 | 139.2 | 13.58 | 27.94 | 14.05 | 39.45 | 17.64 | 406 | 9.8 | 0.09 | 10.21 | 37.8 | 0.29 | 199 | 7.08 |
| 17 | GQ3532073 | 09-02-2010 | 830 | 130 | 10.96 | 25.67 | 7.5 | 39.5 | 76 | 458 | 1 | 1 | 9.75 | 36 | 0.55 | 200 | 7.02 |
| 18 | GQ3532073 | 01-02-2013 | 820 | 131.2 | 12.66 | 22.75 | 6.85 | 35 | 76 | 464 | 5 | 3.75 | 9.2 | 34 | 0.25 | 175 | 7.25 |
| 19 | GQ3532073 | 01-02-2014 | 810 | 132.8 | 12.91 | 24.37 | 6.5 | 37.5 | 5.78 | 470 | 4 | 3.25 | 8.8 | 35.15 | 0.23 | 180 | 7.4 |
| 20 | GQ3532073 | 01-11-2014 | 800 | 134.4 | 13.15 | 24.86 | 5.75 | 38.25 | 4.4 | 517 | 6 | 3.8 | 9.1 | 34.1 | 0.27 | 190 | 7.55 |
| 21 | GQ3532073 | 14-11-2015 | 800 | 134.4 | 13.15 | 24.86 | 5.75 | 38.25 | 4.4 | 517 | 6 | 3.8 | 9.1 | 34.1 | 0.27 | 190 | 7.55 |
| 22 | GQ3532073 | 01-04-2016 | 684 | 102 | 61 | 25 | 8 | 18 | 35.8 | 485 | 10 | 3 | 6.2 | 32.1 | 0.28 | 200 | 7.1 |
| 23 | GQ3532073 | 05-04-2018 | 375 | 131.2 | 10 | 45.5 | 4.1 | 16 | 48 | 166 | 3.5 | 3.6 | 6.39 | 34 | 0.08 | 158 | 3 |
| 24 | GQ3532073 | 25-07-2018 | 400 | 139.6 | 12.91 | 19.33 | 5.75 | 50 | 72 | 224 | 1 | 4 | 3.5 | 31.4 | 0.69 | 200 | 6.7 |
| 25 | GQ3532073 | 19-09-2018 | 521 | 102 | 24.42 | 26.9 | 4.1 | 16 | 46.8 | 401 | 8 | 1 | 3.5 | 37.8 | 0.23 | 158 | 6.6 |
| 26 | GQ3532073 | 16-11-2019 | 542 | 132 | 30.07 | 26.9 | 5.75 | 48 | 35.8 | 505 | 1 | 4.5 | 3.5 | 37.8 | 0.23 | 190 | 6.5 |

Note: All units are mg/L except pH (-).

**Table A18 Groundwater Quality of Kishorganj Sadar**

| **SL** | **WELL ID** | **DATETIME** | **TDS** | **CALCIUM** | **MAGNESIUM** | **SODIUM** | **POTASSIUM** | **CHLORIDE** | **CARBONATE** | **BICARBONATE** | **SULPHATE** | **NITRATE** | **IRON** | **SILICA** | **FLUORIDE** | **CARBONDIOXIDE** | **PH** |
| --- | --- | --- | --- | --- | --- | --- | --- | --- | --- | --- | --- | --- | --- | --- | --- | --- | --- |
| 1 | GQ4849046 | 01-03-1985 | 340 | 61.5 | 29.7 | 16.1 | 4.75 | 2.87 | 6 | 292.9 | 31.9 | 2.25 | 1.16 | 57.75 | 0.38 | 10.05 | 6.9 |
| 2 | GQ4849046 | 01-03-1986 | 251 | 18.53 | 13.67 | 54.26 | 7.25 | 14.93 | 11.79 | 119.6 | 41.65 | 0.65 | 1.65 | 29.6 | 0.52 | 3.98 | 7.42 |
| 3 | GQ4849046 | 01-03-1990 | 315 | 25.3 | 12 | 58.54 | 4.15 | 15.9 | 10.67 | 243.9 | 16.66 | 1.5 | 1.58 | 45 | 0.56 | 12.33 | 8.32 |
| 4 | GQ4849046 | 01-03-1992 | 166 | 25 | 21 | 5 | 2.75 | 8 | 7 | 212 | 7 | 4.8 | 1.65 | 28 | 0.38 | 74 | 7.2 |
| 5 | GQ4849046 | 01-03-1993 | 214 | 42 | 63 | 1.95 | 4 | 3 | 6.57 | 147 | 1 | 0.7 | 1.12 | 9.1 | 0.38 | 36 | 7.18 |
| 6 | GQ4849046 | 15-11-1994 | 190 | 54.8 | 19.29 | 0.91 | 2.75 | 1.4 | 6 | 175 | 13 | 0.6 | 1.54 | 44.9 | 0.38 | 74 | 7.51 |
| 7 | GQ4849046 | 17-06-1996 | 160 | 22.4 | 21 | 1.04 | 7.75 | 1.6 | 7.2 | 179 | 4 | 1.3 | 2.99 | 39.9 | 0.16 | 50 | 8.14 |
| 8 | GQ4849046 | 13-04-1997 | 180 | 64.4 | 26.37 | 0.91 | 7.25 | 1.4 | 7 | 185 | 0.29 | 1 | 1.67 | 54.5 | 0.34 | 94 | 7.64 |
| 9 | GQ4849046 | 29-10-1997 | 180 | 44 | 15.13 | 1.3 | 1.78 | 2 | 7 | 146 | 9 | 0.9 | 1.17 | 10.2 | 0.3 | 90 | 9.09 |
| 10 | GQ4849046 | 18-07-1998 | 315 | 22.93 | 11.06 | 32.7 | 1.78 | 2.4 | 10.67 | 214 | 0.11 | 0.2 | 0.75 | 20.92 | 0.3 | 75 | 8 |
| 11 | GQ4849046 | 15-12-1998 | 215 | 56 | 19.3 | 0.97 | 2.8 | 1.5 | 5.2 | 270 | 13 | 0.65 | 1.6 | 45 | 0.38 | 75 | 7.6 |
| 12 | GQ4849046 | 15-03-2000 | 251 | 56 | 19.3 | 0.97 | 2.8 | 1.5 | 7.2 | 270 | 1.86 | 0.65 | 1.6 | 45 | 0.38 | 78 | 7.6 |
| 13 | GQ4849046 | 18-02-2001 | 400 | 56 | 19.4 | 0.98 | 2.75 | 1.5 | 6 | 270 | 11 | 0.65 | 1.6 | 48 | 0.35 | 75 | 7.62 |
| 14 | GQ4849046 | 07-01-2005 | 410 | 56 | 19.4 | 0.98 | 2.75 | 1.5 | 6 | 265 | 13 | 0.65 | 1.6 | 48 | 0.35 | 75 | 7.62 |
| 15 | GQ4849046 | 01-05-2008 | 199 | 63.6 | 31.36 | 0.96 | 4 | 1.49 | 6 | 219 | 0.29 | 0.98 | 1.19 | 65.4 | 0.67 | 79 | 7.46 |
| 16 | GQ4849046 | 24-01-2010 | 180 | 60 | 14.61 | 1.62 | 3.75 | 2.5 | 6 | 255 | 2 | 1.75 | 1 | 55 | 0.4 | 84.1 | 7.7 |
| 17 | GQ4849046 | 01-02-2013 | 220 | 63.2 | 12.66 | 3.9 | 4.15 | 6 | 6.57 | 247 | 9 | 2.75 | 0.75 | 55 | 0.45 | 70 | 7.5 |
| 18 | GQ4849046 | 01-02-2014 | 230 | 65.2 | 13.64 | 5.2 | 4.75 | 8 | 6 | 254 | 11 | 3.5 | 0.8 | 56 | 0.48 | 75 | 7.25 |
| 19 | GQ4849046 | 01-11-2014 | 237 | 62 | 12.18 | 4.64 | 3.8 | 7.15 | 6 | 233 | 13 | 3.75 | 0.7 | 57.75 | 0.48 | 78 | 7.2 |
| 20 | GQ4849046 | 14-11-2015 | 237 | 62 | 12.18 | 4.64 | 3.8 | 7.15 | 7 | 233 | 13 | 3.75 | 0.7 | 57.75 | 0.48 | 78 | 7.2 |
| 21 | GQ4849046 | 01-04-2016 | 215 | 63.2 | 21 | 1 | 3 | 2.3 | 6.57 | 310 | 1.86 | 2.25 | 1.6 | 46 | 0.41 | 82 | 7.5 |
| 22 | GQ4849046 | 26-03-2018 | 127.1 | 63.2 | 21 | 3.9 | 4.75 | 10 | 5.2 | 184 | 1 | 0.1 | 0.26 | 20.92 | 0.52 | 75 | 5.85 |
| 23 | GQ4849046 | 02-09-2018 | 173 | 22.4 | 19.4 | 32.7 | 4.15 | 13 | 12 | 119.6 | 27 | 0.5 | 5.84 | 65.4 | 0.56 | 90 | 6.7 |
| 24 | GQ4849046 | 11-06-2019 | 166 | 18.53 | 31.36 | 16.1 | 4 | 37.5 | 7.2 | 180 | 3 | 1.1 | 5.26 | 28 | 0.04 | 12.33 | 6.7 |
| 25 | GQ4849046 | 09-12-2019 | 167.5 | 56 | 29.7 | 0.91 | 1.78 | 13 | 7 | 254 | 27 | 1.5 | 4.99 | 45 | 0.56 | 82 | 6.7 |

Note: All units are mg/L except pH (-).

**Table A19 Groundwater Quality of Mohammadpur**

| **SL** | **WELL ID** | **DATETIME** | **TDS** | **CALCIUM** | **MAGNESIUM** | **SODIUM** | **POTASSIUM** | **CHLORIDE** | **CARBONATE** | **BICARBONATE** | **SULPHATE** | **NITRATE** | **IRON** | **SILICA** | **FLUORIDE** | **CARBONDIOXIDE** | **PH** |
| --- | --- | --- | --- | --- | --- | --- | --- | --- | --- | --- | --- | --- | --- | --- | --- | --- | --- |
| 1 | GQ2650102 | 01-03-1985 | 289 | 19.8 | 9.9 | 69.87 | 6.5 | 17.89 | 3.52 | 139.8 | 93.1 | 1.8 | 1.3 | 33.8 | 0.17 | 1 | 6.9 |
| 2 | GQ2650102 | 01-03-1986 | 275 | 17.54 | 12.45 | 61.73 | 4.28 | 14.87 | 11.39 | 119.6 | 63.7 | 3.25 | 5.9 | 33.8 | 0.4 | 5.99 | 6.95 |
| 3 | GQ2650102 | 01-03-1993 | 130 | 33.6 | 50.4 | 1.3 | 12 | 2 | 76 | 76 | 3 | 1.4 | 0.03 | 1.11 | 0.1 | 22 | 6.31 |
| 4 | GQ2650102 | 31-12-1994 | 150 | 49.6 | 14.65 | 6.3 | 2.75 | 9.7 | 76 | 112 | 1 | 0.9 | 0.03 | 66.1 | 0.17 | 80 | 6.42 |
| 5 | GQ2650102 | 13-06-1996 | 220 | 7.6 | 14.66 | 7.08 | 6.5 | 10 | 64 | 107 | 3 | 1.8 | 0.99 | 50.2 | 0.12 | 58 | 8.8 |
| 6 | GQ2650102 | 03-04-1997 | 150 | 49.2 | 15.14 | 9.95 | 25 | 15.3 | 23.52 | 114 | 7 | 1 | 0.03 | 66.7 | 0.4 | 42 | 6.71 |
| 7 | GQ2650102 | 16-07-1998 | 245 | 29.03 | 10.81 | 22.6 | 2.05 | 15.9 | 64 | 170 | 5.16 | 0.8 | 0.1 | 30.44 | 0.19 | 58 | 8.25 |
| 8 | GQ2650102 | 17-12-1998 | 275 | 50 | 14.66 | 2.6 | 2.8 | 4 | 76 | 228 | 1 | 0.91 | 0.04 | 45 | 0.1 | 82 | 6.45 |
| 9 | GQ2650102 | 16-03-2000 | 289 | 50 | 15.59 | 2.6 | 2.75 | 4 | 76 | 228 | 1 | 0.9 | 0.05 | 45 | 0.1 | 80 | 6.5 |
| 10 | GQ2650102 | 04-01-2001 | 650 | 49.6 | 10.81 | 1.3 | 12 | 15.9 | 30.6 | 255 | 27 | 0.31 | 0.19 | 1.11 | 2.2 | 150 | 7.6 |
| 11 | GQ2650102 | 22-02-2001 | 275 | 50 | 15.6 | 2.7 | 2.75 | 4 | 30.6 | 228 | 1 | 0.9 | 0.06 | 45 | 0.1 | 82 | 6.7 |
| 12 | GQ2650102 | 25-12-2004 | 280 | 50 | 15.6 | 2.7 | 2.75 | 4 | 30.6 | 230 | 1 | 0.9 | 0.07 | 45 | 0.1 | 85 | 6.7 |
| 13 | GQ2650102 | 01-06-2008 | 236 | 48 | 16.45 | 3.32 | 4.28 | 5.97 | 23.52 | 166 | 0.97 | 0.98 | 0.07 | 61.2 | 0.19 | 69 | 7.03 |
| 14 | GQ2650102 | 04-02-2010 | 250 | 46 | 8.52 | 3.08 | 3.5 | 4.75 | 30.6 | 182 | 2 | 1 | 0.07 | 47 | 0.17 | 75 | 6.98 |
| 15 | GQ2650102 | 01-02-2013 | 240 | 47.6 | 7.55 | 3.41 | 3.5 | 5.25 | 76 | 178 | 4 | 3.25 | 0.15 | 42 | 0.2 | 70 | 6.7 |
| 16 | GQ2650102 | 01-02-2014 | 230 | 45.2 | 7.8 | 4.06 | 2.75 | 6.25 | 64 | 170 | 5 | 4.1 | 0.1 | 43.15 | 0.18 | 75 | 7.1 |
| 17 | GQ2650102 | 01-11-2014 | 245 | 47.2 | 8.53 | 4.64 | 3.15 | 7.15 | 30.6 | 178 | 6 | 4.75 | 0.19 | 44.5 | 0.13 | 68 | 7.2 |
| 18 | GQ2650102 | 14-11-2015 | 245 | 47.2 | 8.53 | 4.64 | 3.15 | 7.15 | 64 | 178 | 6 | 4.75 | 0.19 | 44.5 | 0.13 | 68 | 7.2 |
| 19 | GQ2650102 | 01-04-2016 | 358 | 40 | 24 | 2.98 | 1 | 36 | 23.52 | 240 | 6 | 1 | 0.2 | 50 | 0.12 | 90 | 7.8 |
| 20 | GQ2650102 | 29-03-2018 | 187.8 | 49.2 | 15.59 | 7.08 | 3.5 | 17 | 12 | 142 | 8 | 0.31 | 7.06 | 66.1 | 0.18 | 85 | 6.07 |
| 21 | GQ2650102 | 08-09-2018 | 259 | 40 | 15.59 | 9.95 | 2.05 | 18 | 88 | 178 | 5 | 0.7 | 5.3 | 66.7 | 0.07 | 42 | 6.3 |
| 22 | GQ2650102 | 10-12-2019 | 273 | 50 | 24 | 7.08 | 2.75 | 51 | 30.6 | 170 | 7 | 0.5 | 3.25 | 66.7 | 0.19 | 82 | 6.3 |

Note: All units are mg/L except pH (-).

Appendix A1


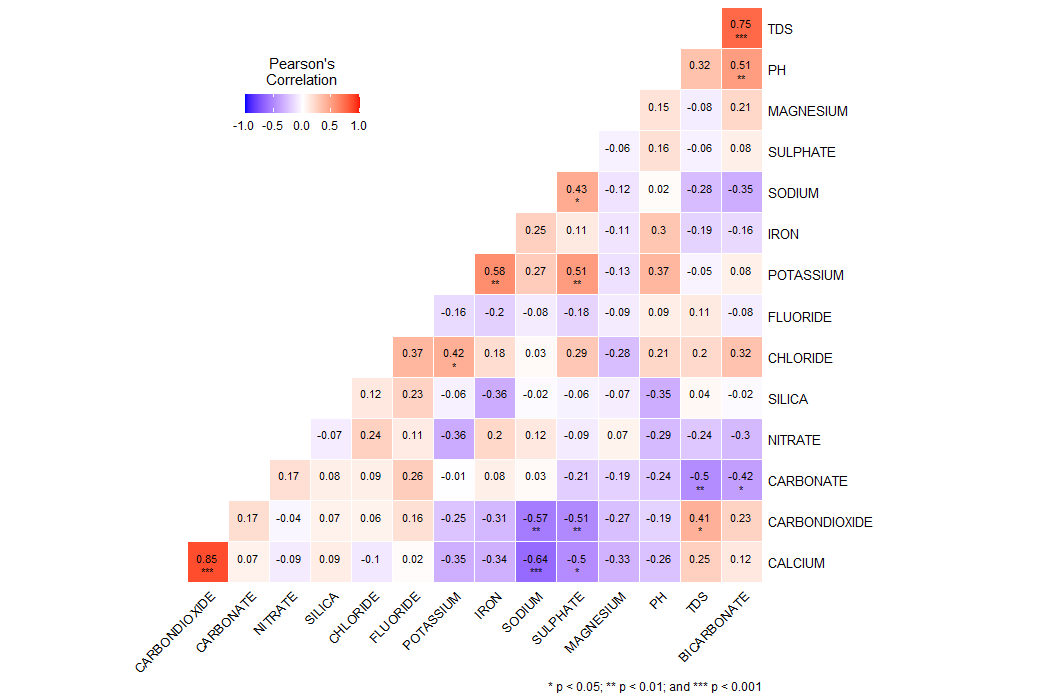

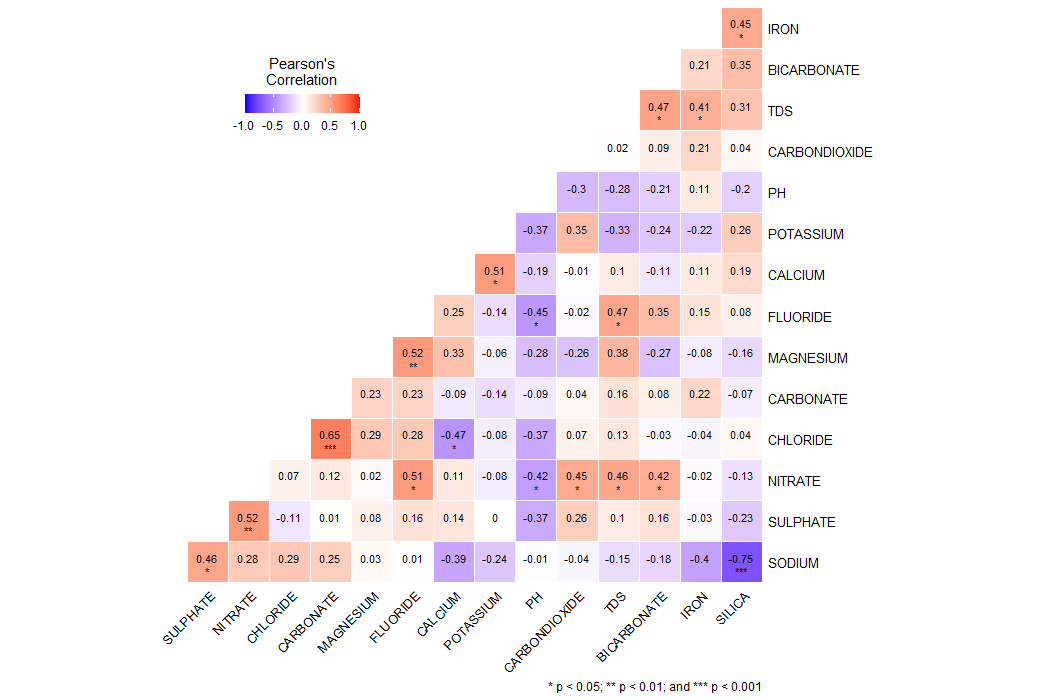


(2) Gopalganj Sadar

(1) Gazipur


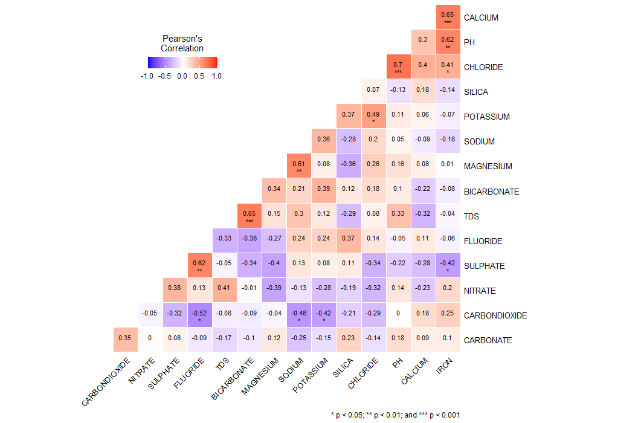


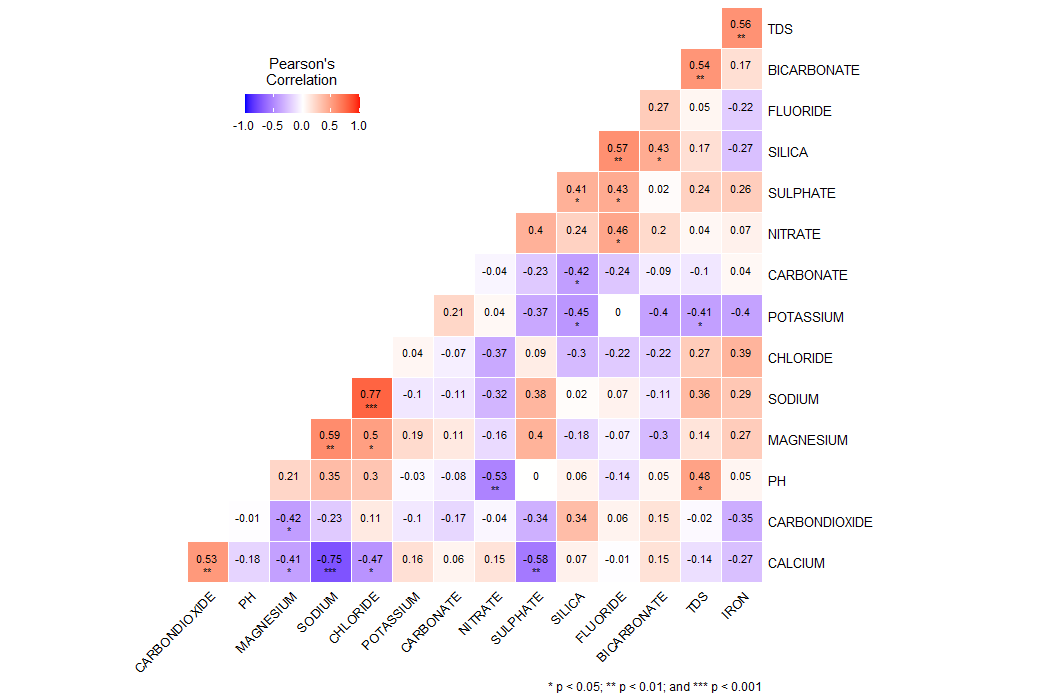


(4) Kishoreganj Bhairab

(3) Gopalganj Kashiani


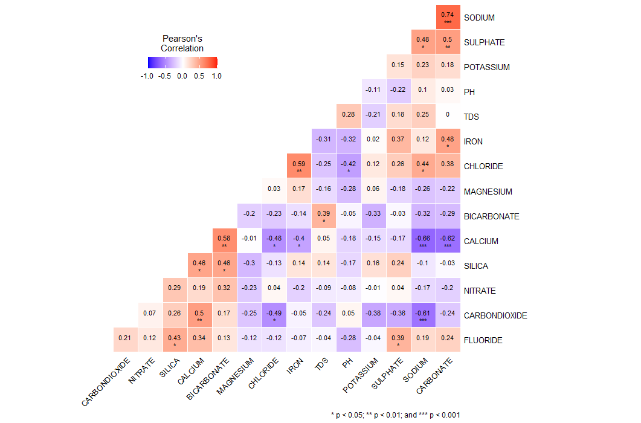


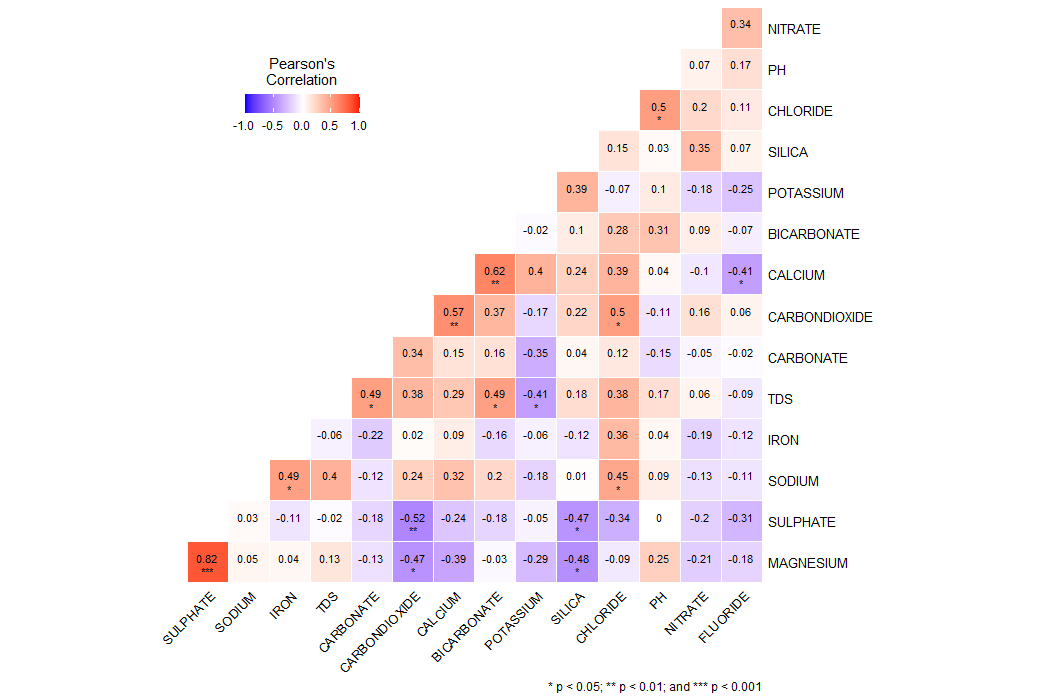


(6) Madaripur Sadar

(5) Kishoreganj Sadar


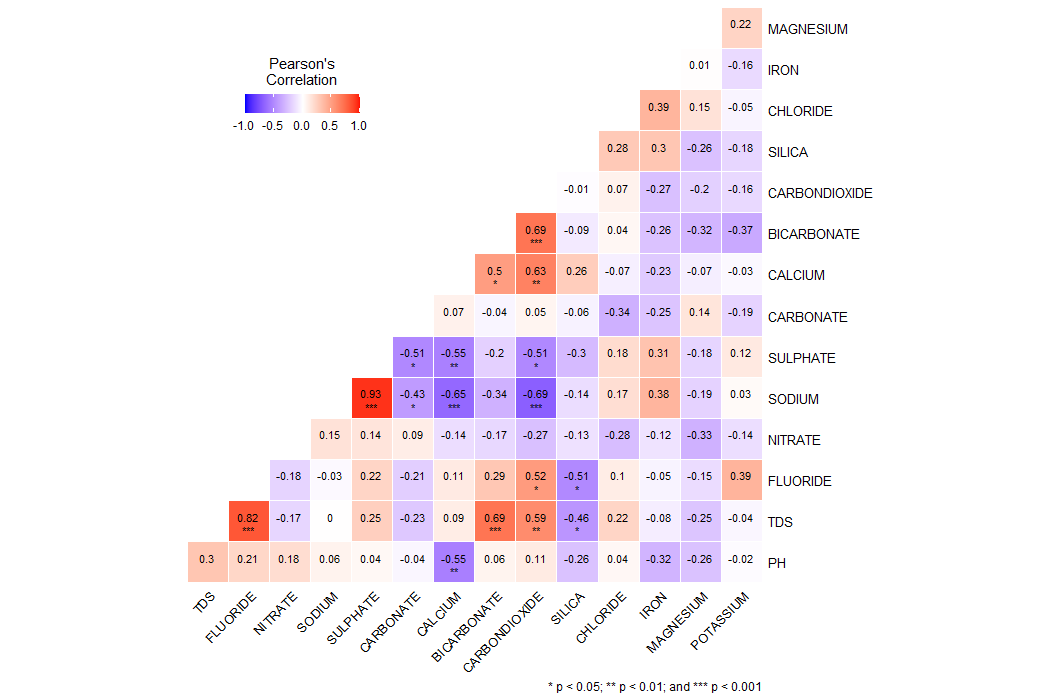


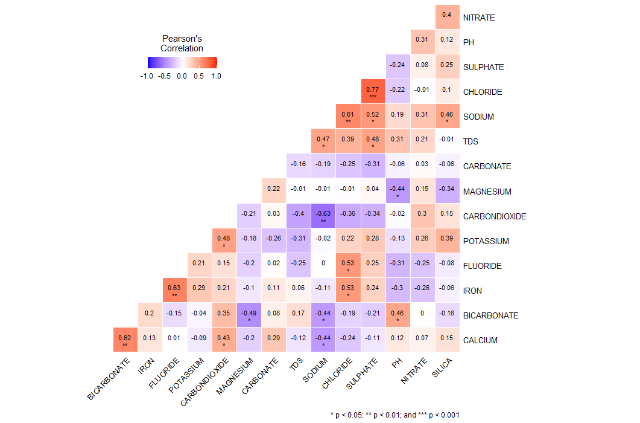


(8) Motijheel

(7) Mohammadpur


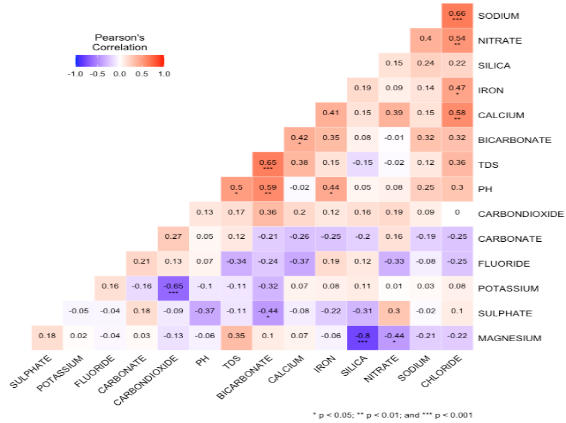


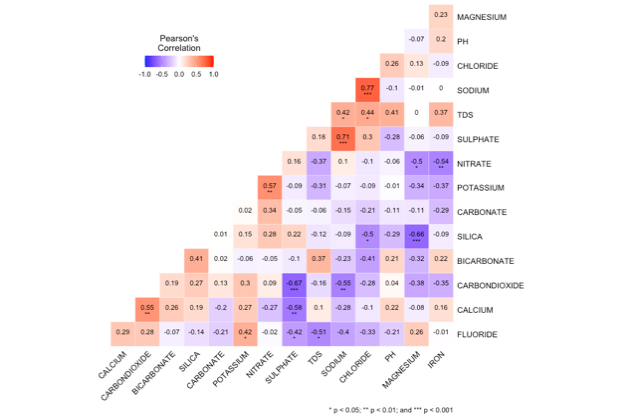


(10) Narsingdi Sadar

(9) Munshiganj


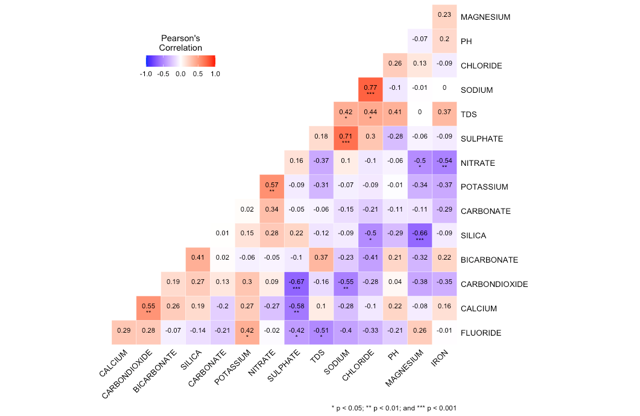


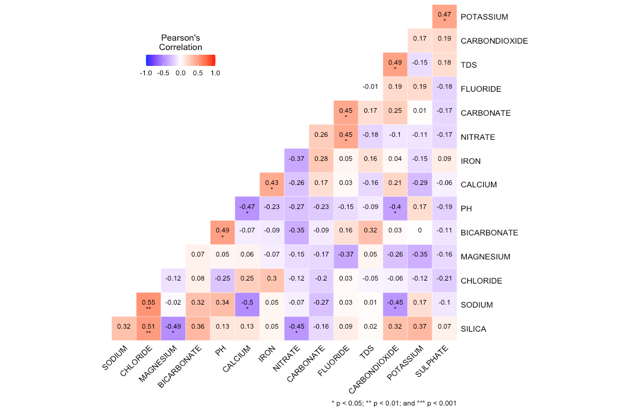


(11) Rajbari Pangsha

(12) Rajbari Sadar


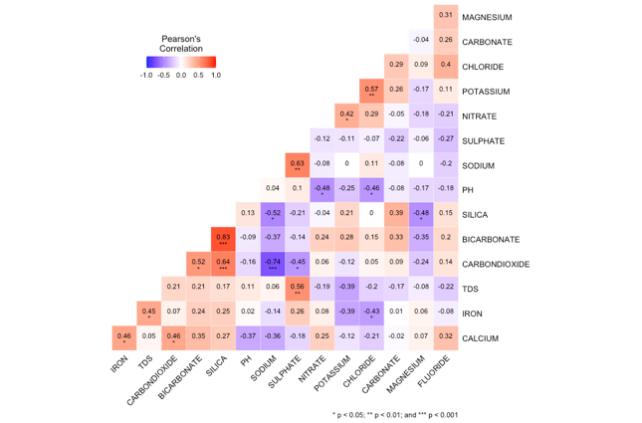

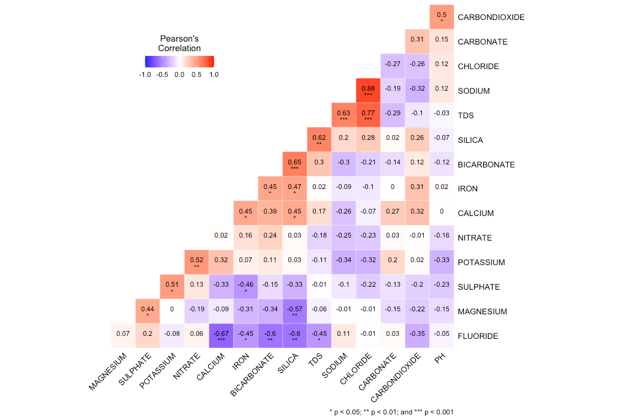


(13) Sherpur Sadar

(14) Tangail Madhupur


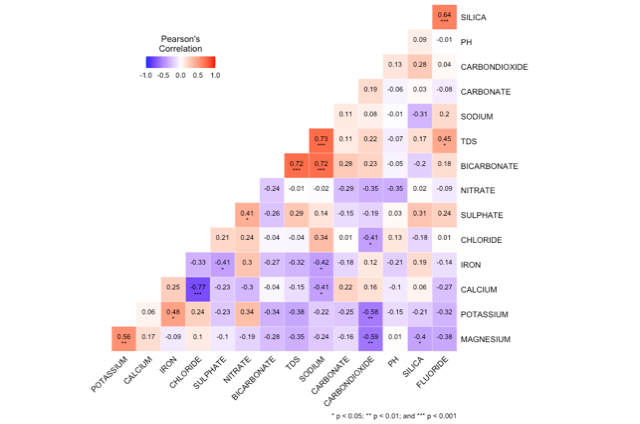

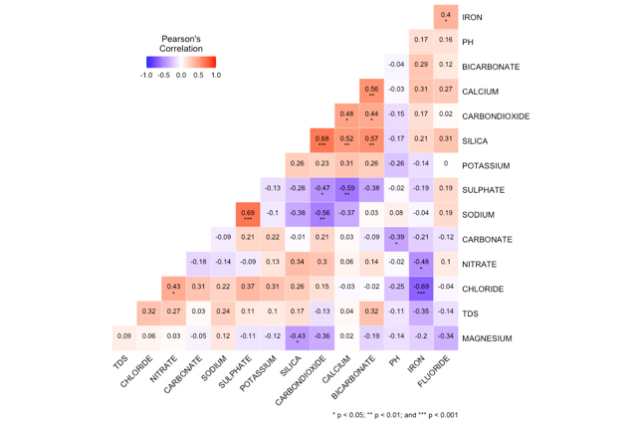


(16) Tangail Mirzapur

(15) Tangail Sadar


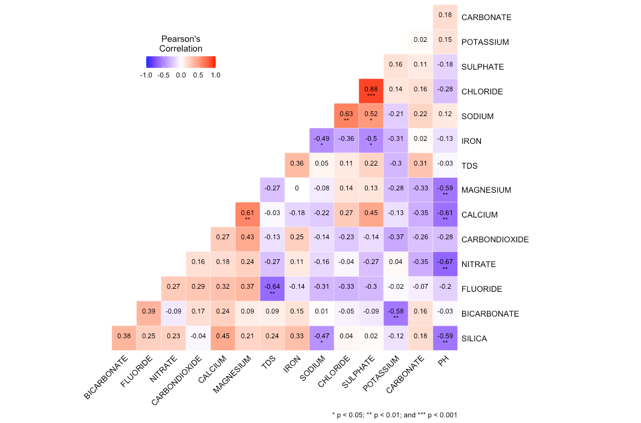


(17) Tangail Textile Mill

**Figure A1 Pearson's correlation matrix**

Appendix A2


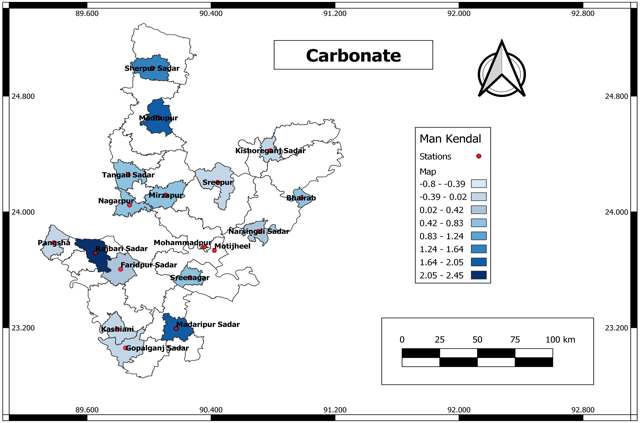

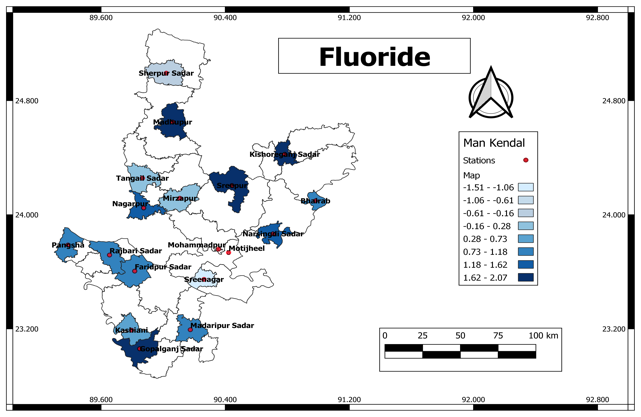

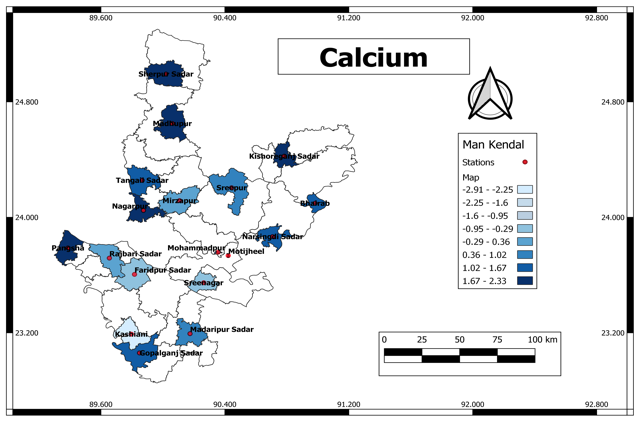

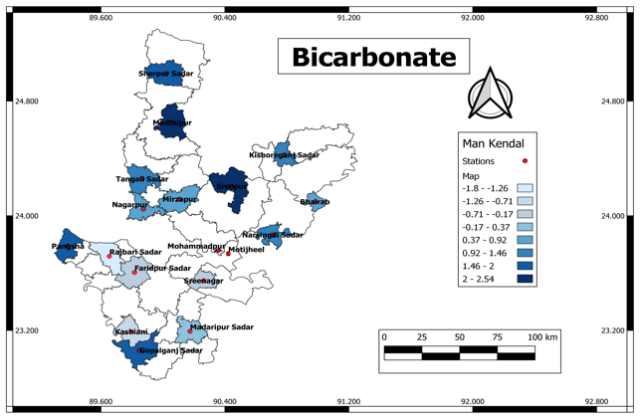

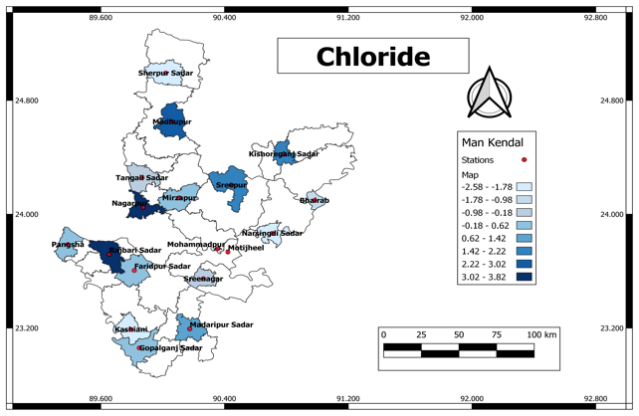

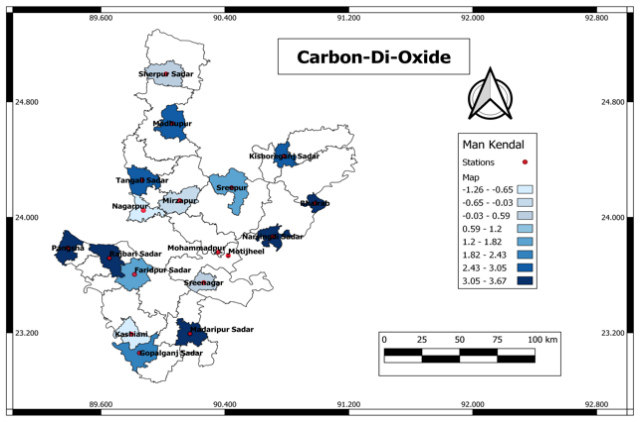


(2) Calcium

(1) Bicarbonate

(4) Carbonate

(3) Carbondioxide

(6) Fluoride

(5) Chloride


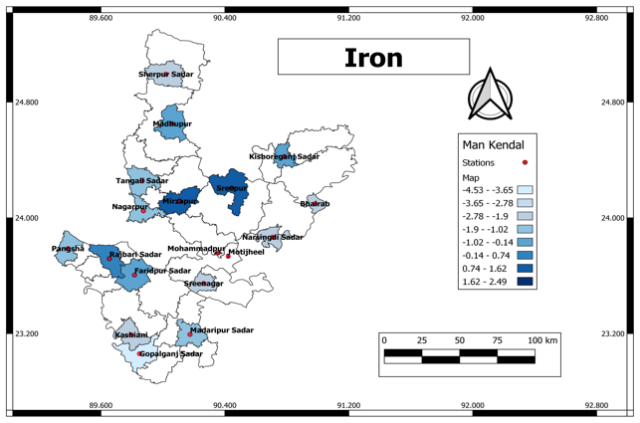

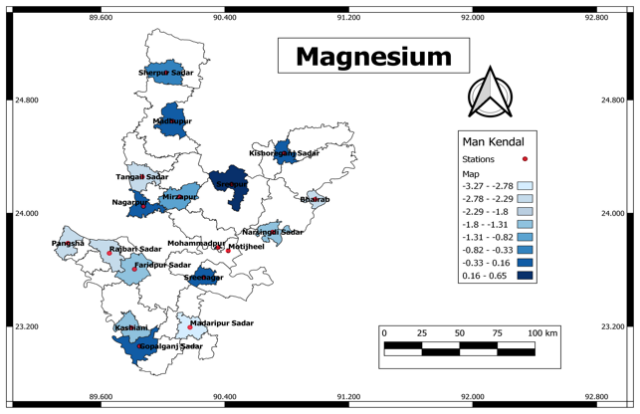


(7) Iron

(8) Magnesium


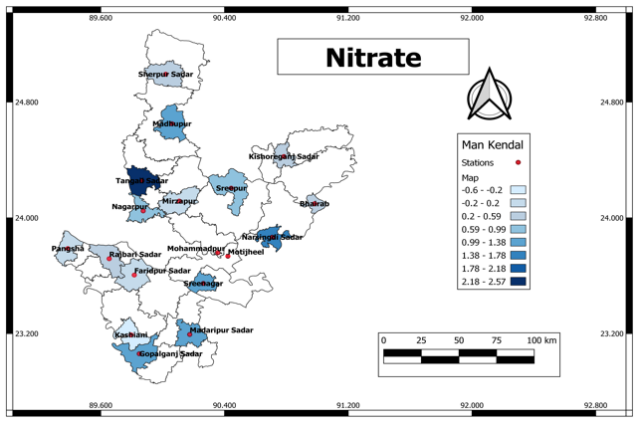

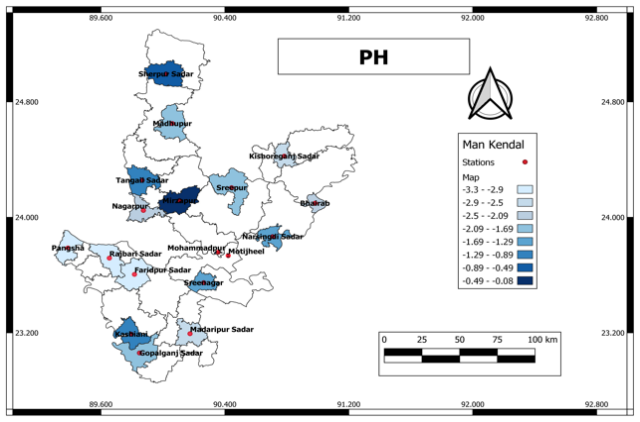


(9) Nitrate

(10) PH


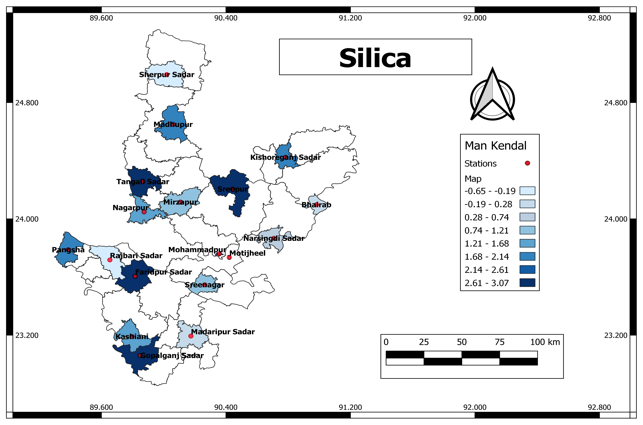

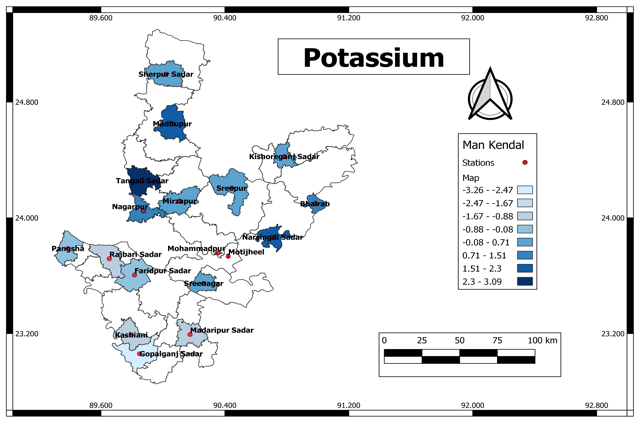


(12) Silica

(11) Potassium


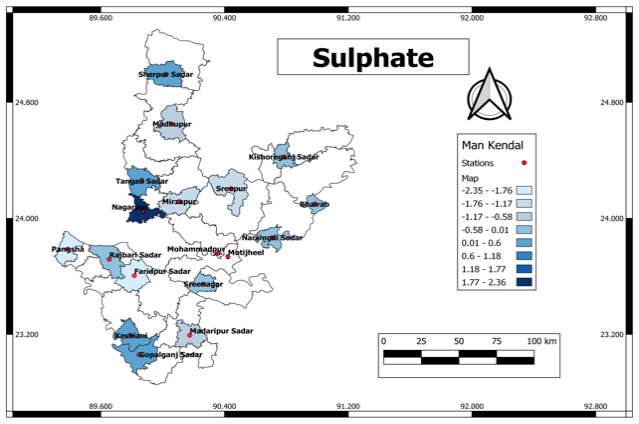

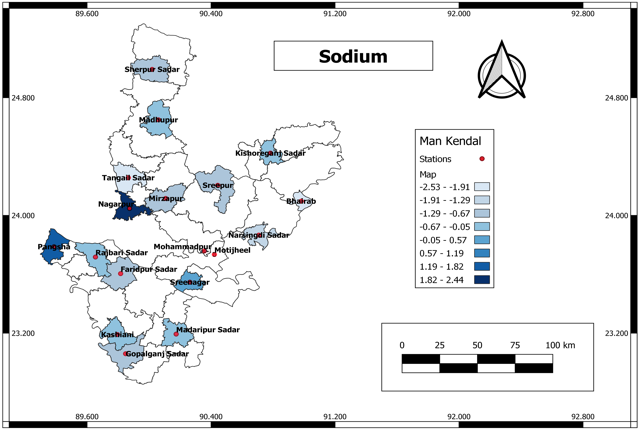


(14) Sulphate

(13) Sodium

**Figure A2 GIS mapping of Man Kendal value**

Appendix A3


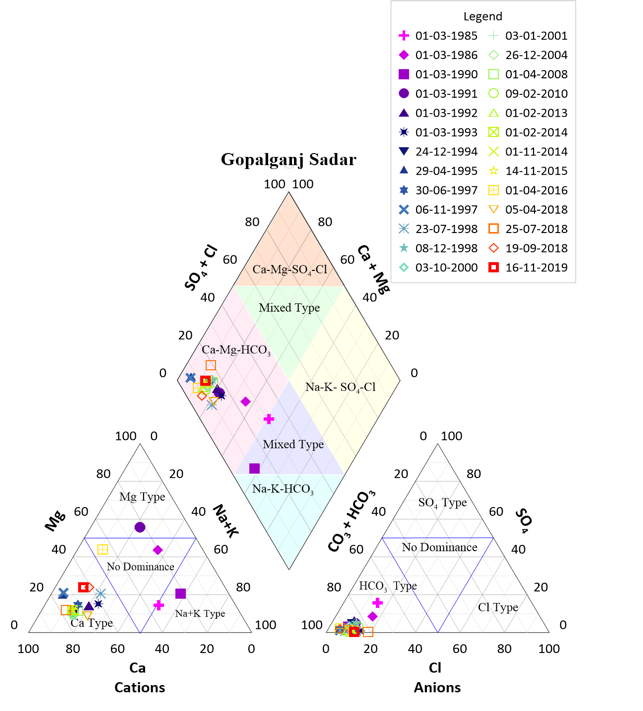

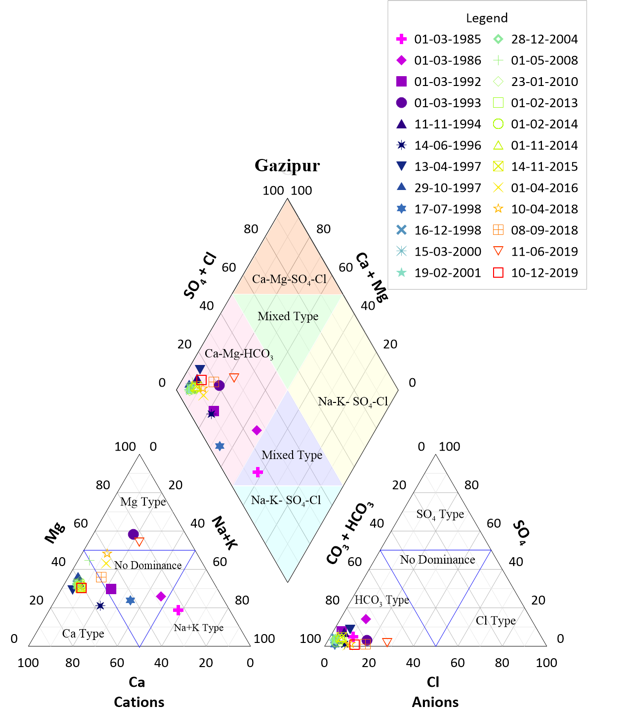


(1) Gazipur

(2) Gopalganj Sadar


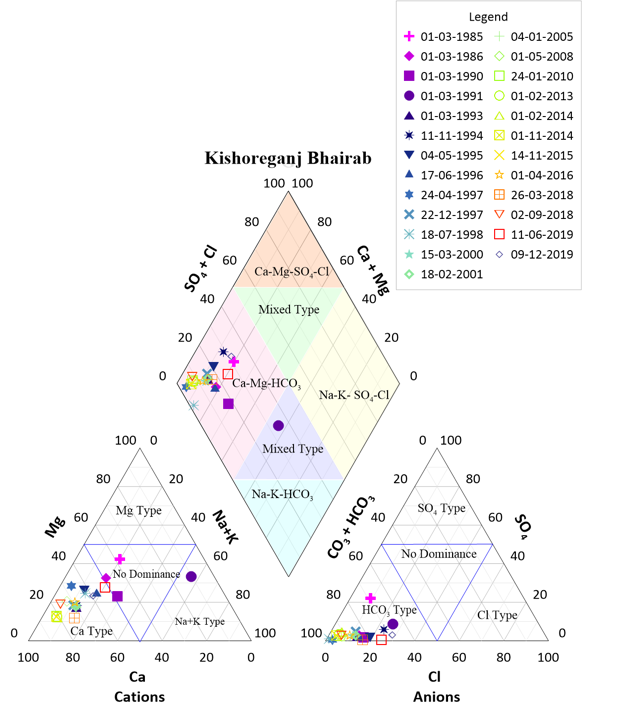

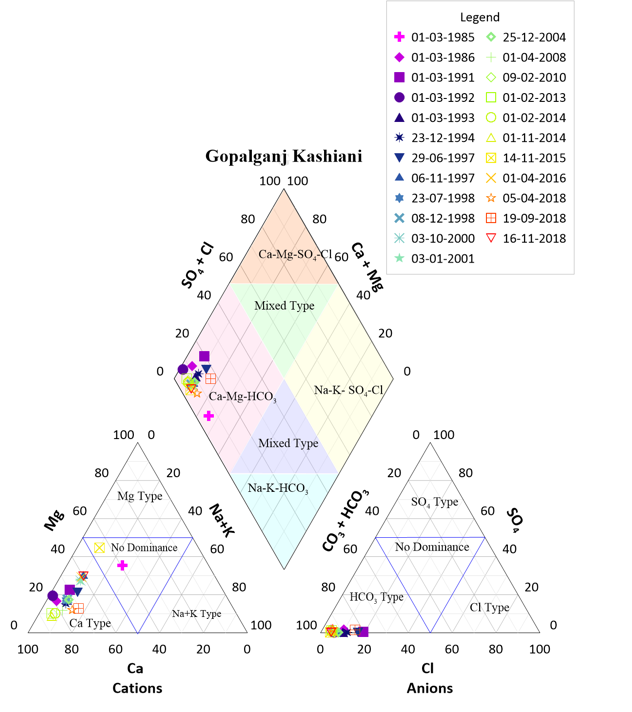


(4) Kishoreganj Bhairab

(3) Gopalganj Kashiani


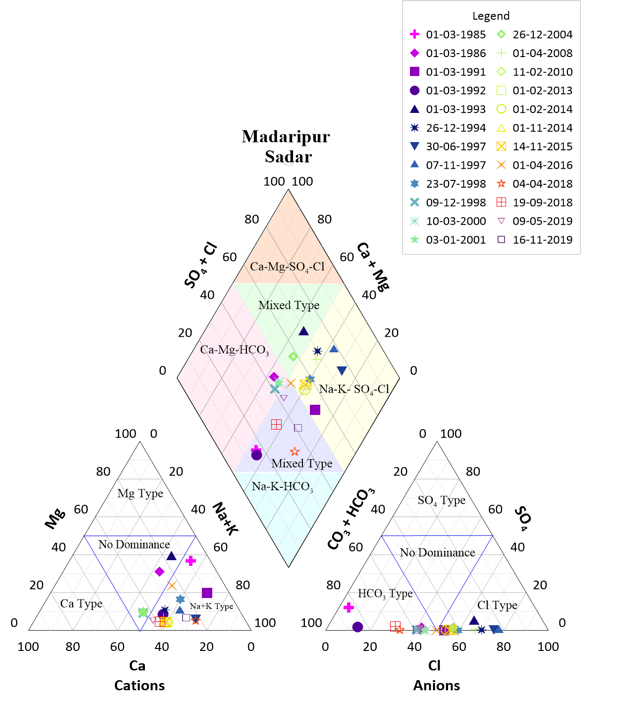

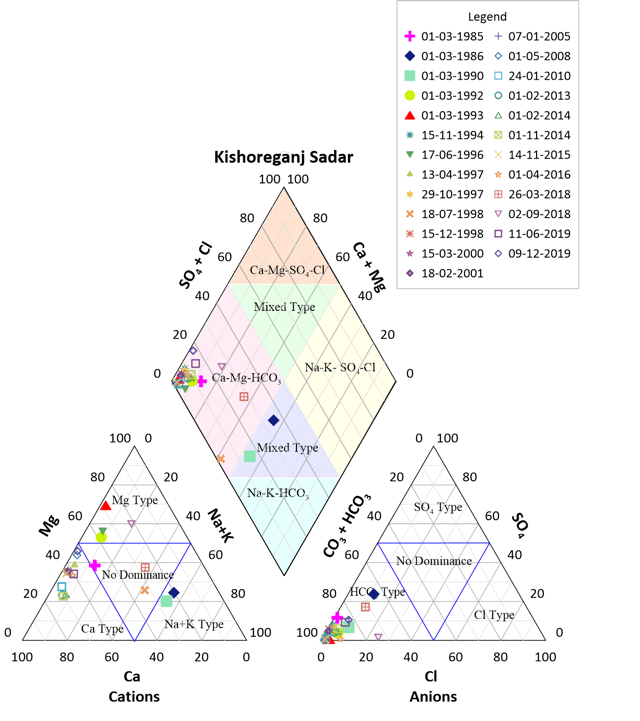


(6) Madaripur Sadar

(5) Kishoreganj Sadar


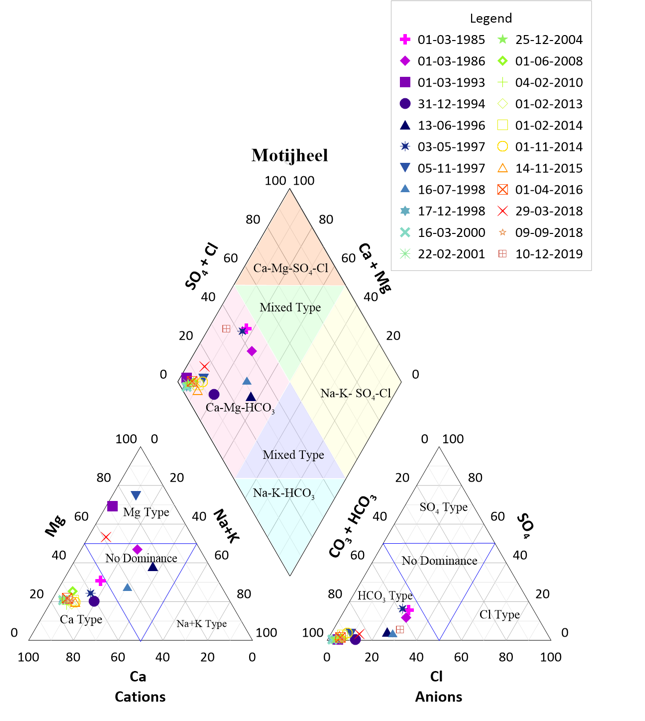

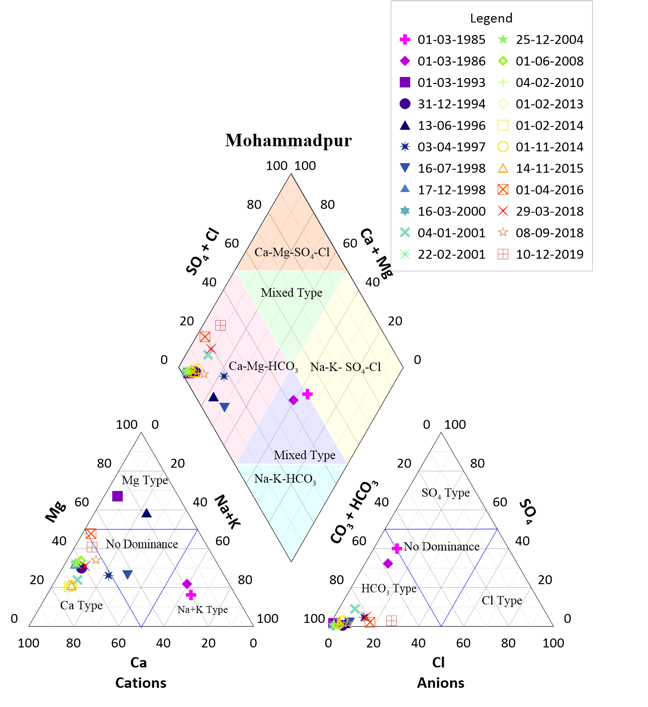


(7) Mohammadpur

(8) Motijheel


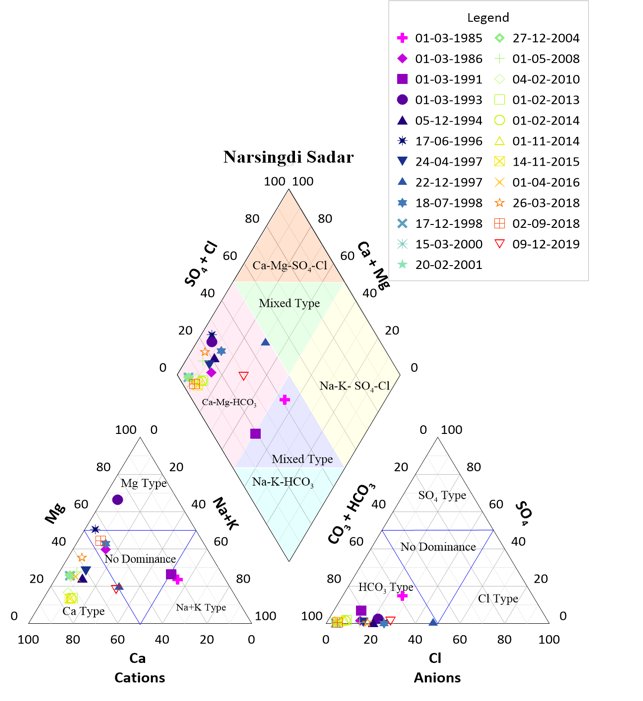

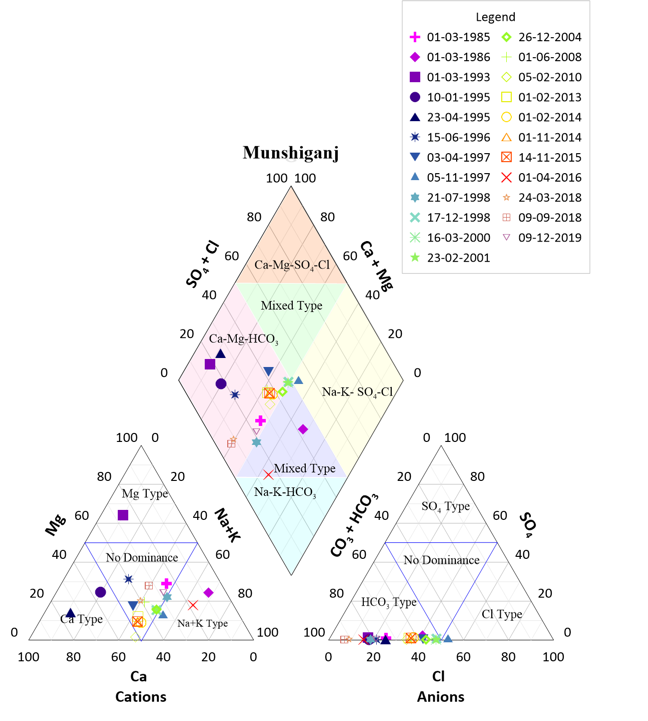


(9) Munshiganj

(10) Narsingdi Sadar


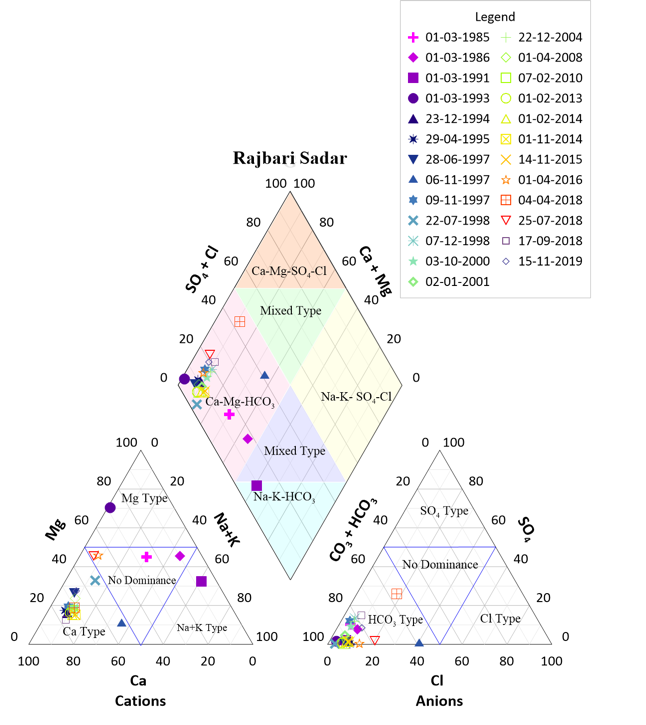

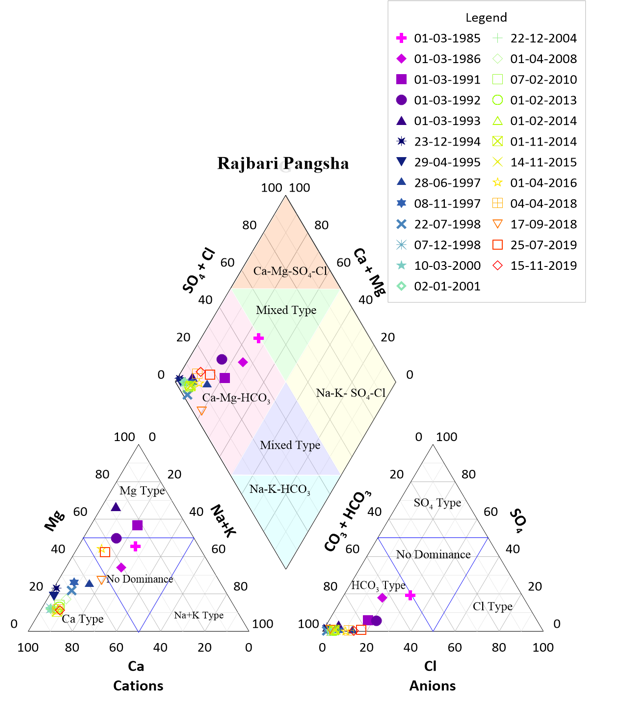


(12) Rajbari Sadar

(11) Rajbari Pangsha


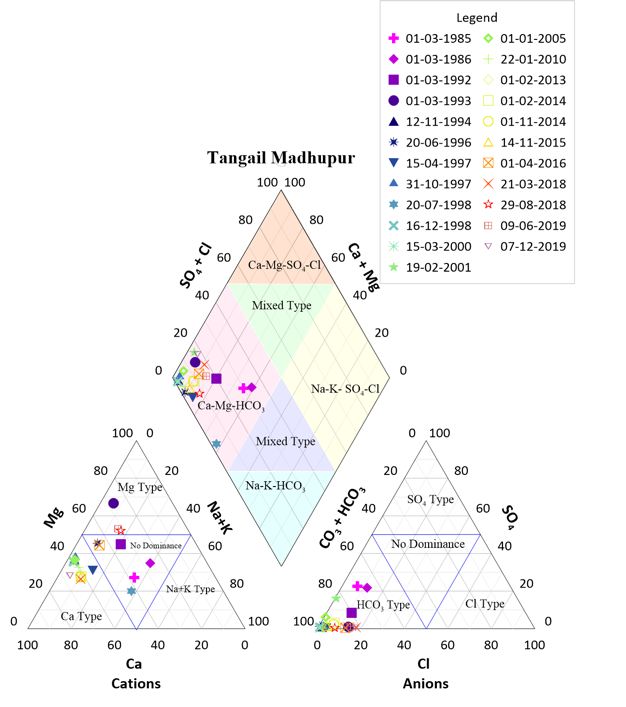

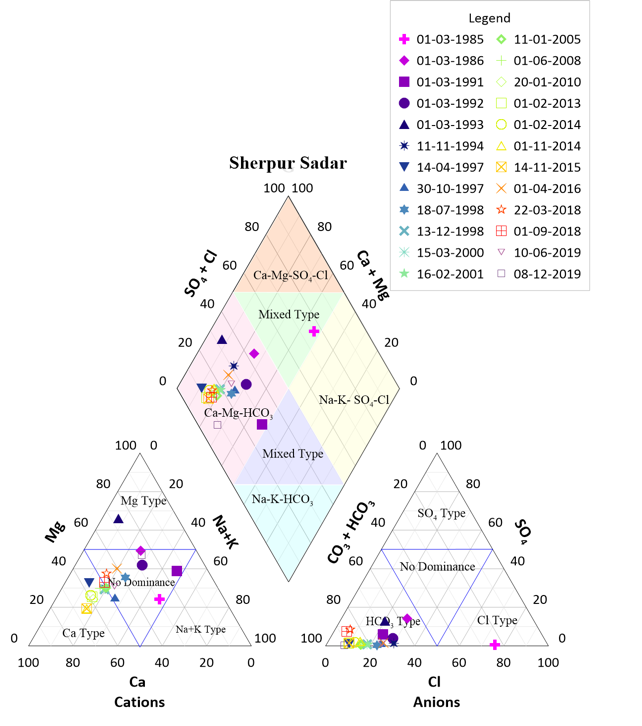


(13) Sherpur Sadar

(14) Tangail Madhupur


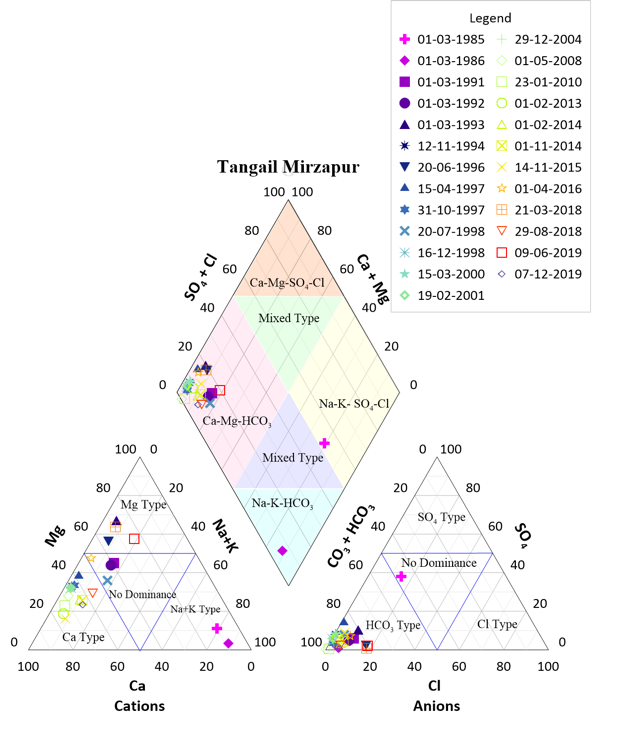

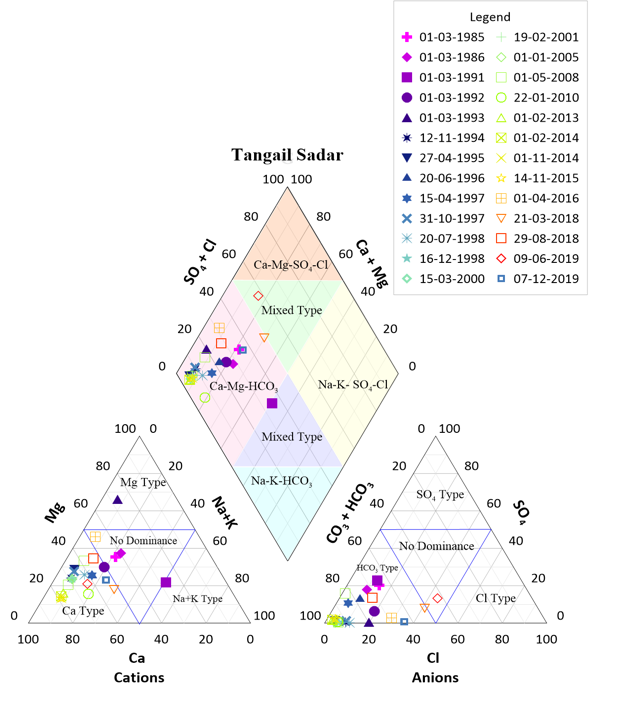


(16) Tangail Mirzapur

(15) Tangail Sadar


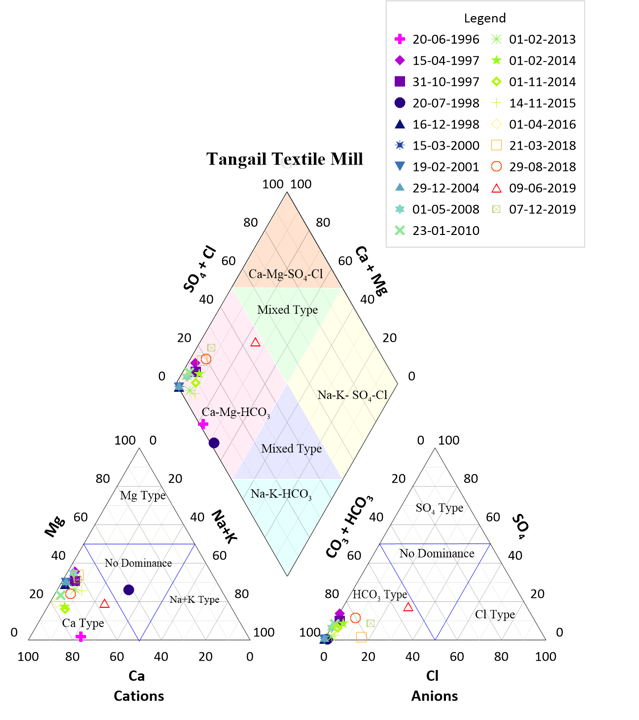


(17) Tangail Textile Mill

**Figure A3 Piper diagram**

Appendix A4


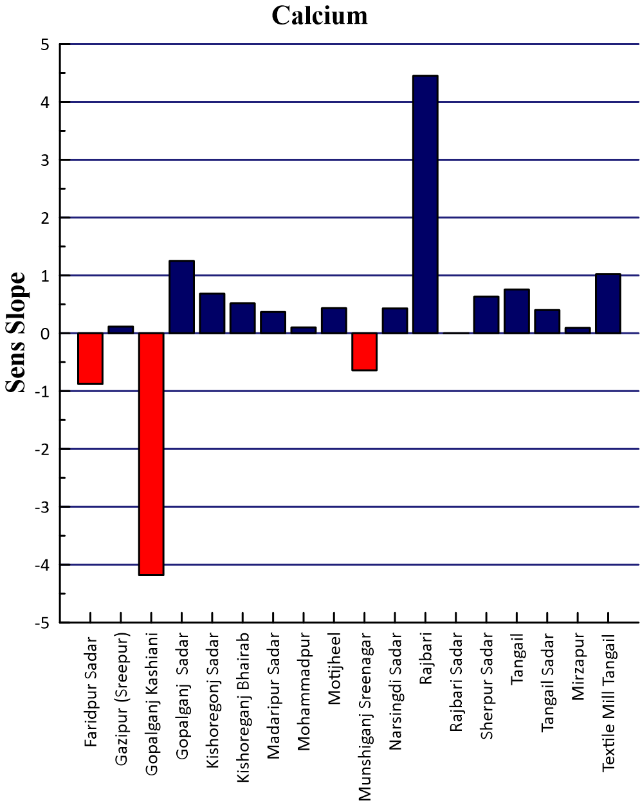

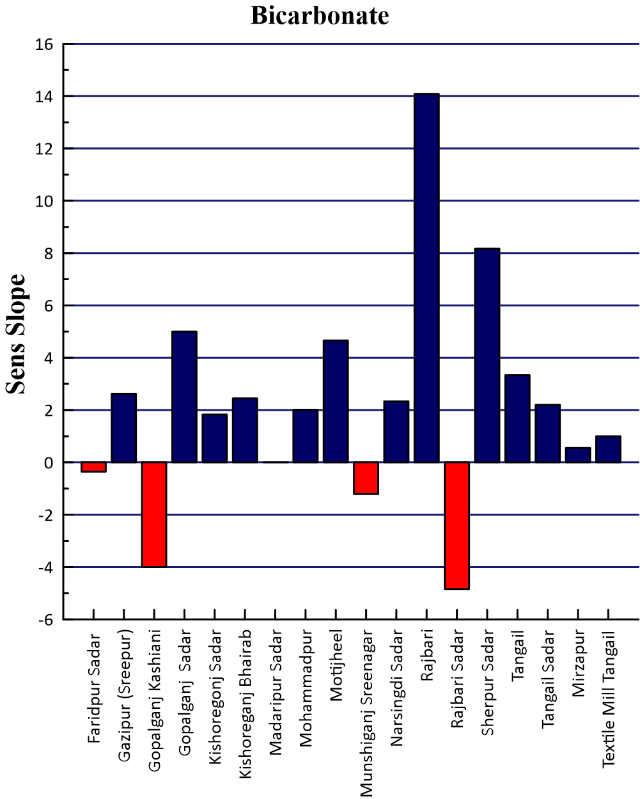


(1) Bicarbonate

(2) Calcium


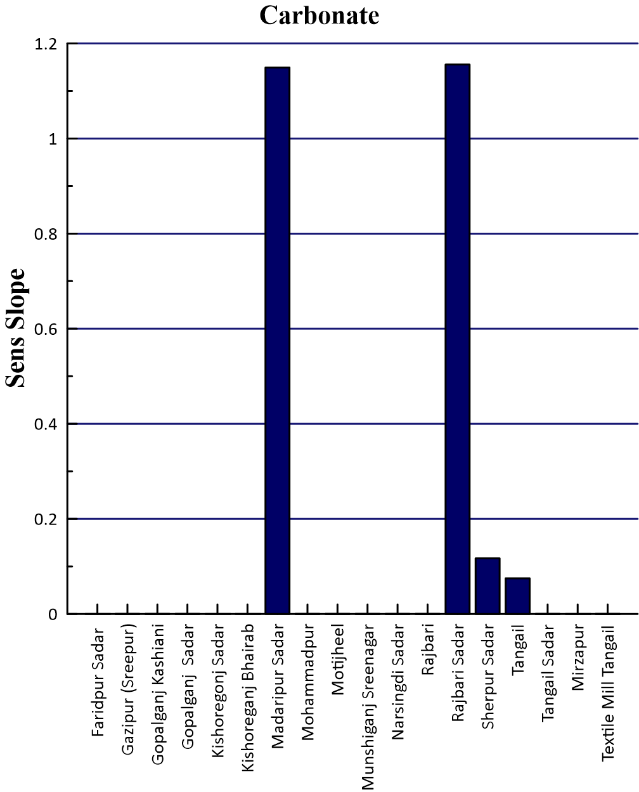

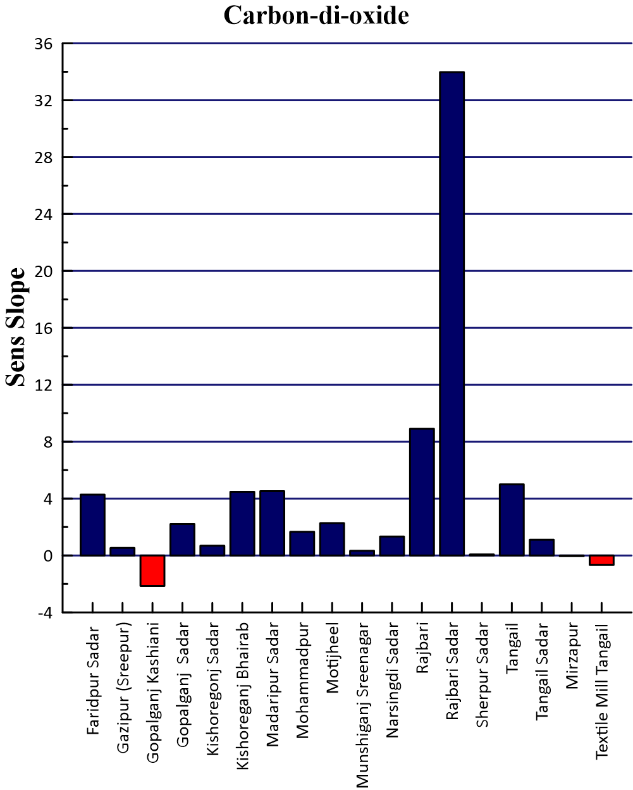


(3) Carbondioxide

(4) Carbonate


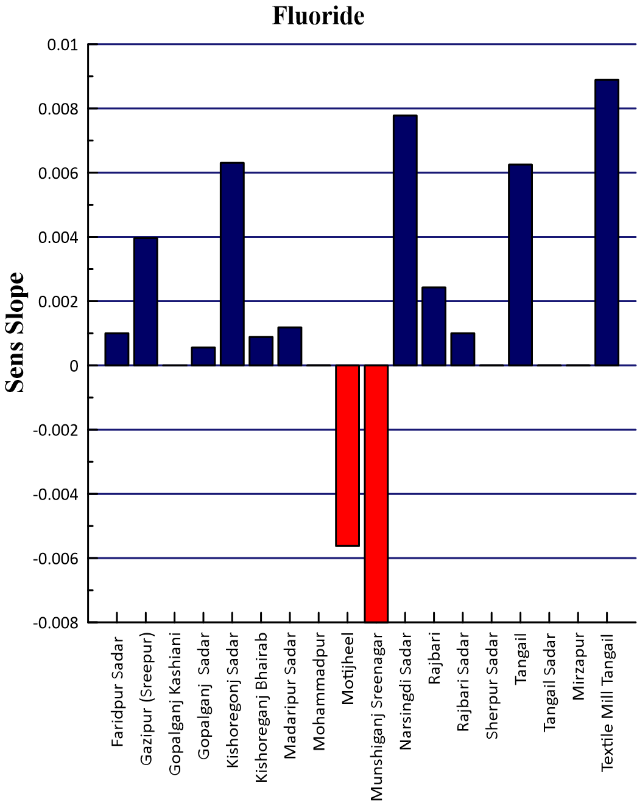

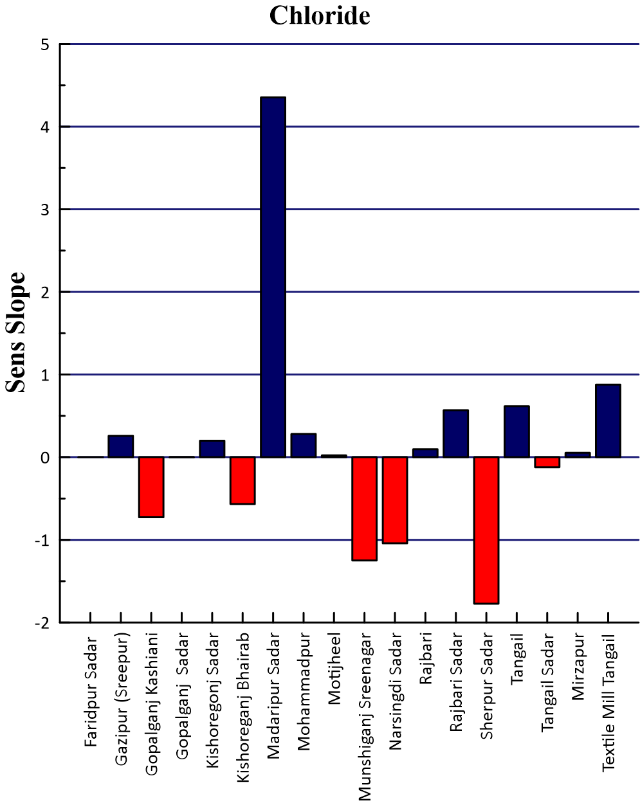


(5) Chloride

(6) Fluoride


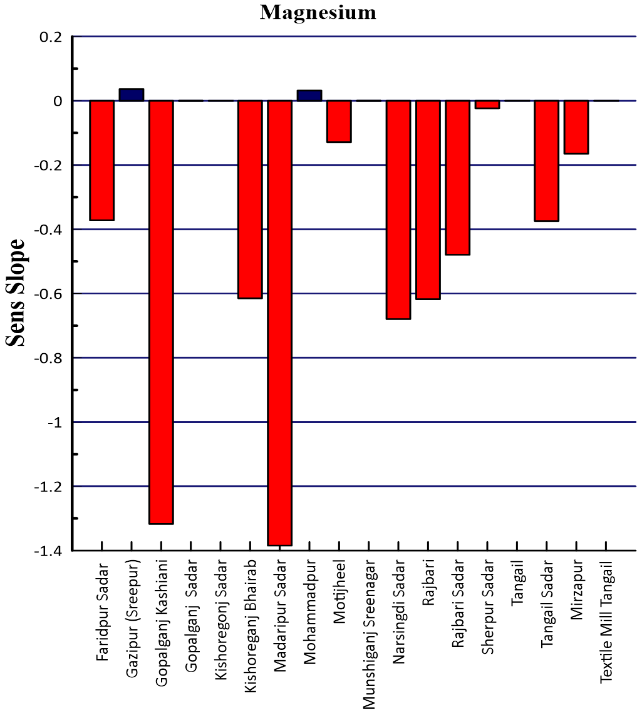

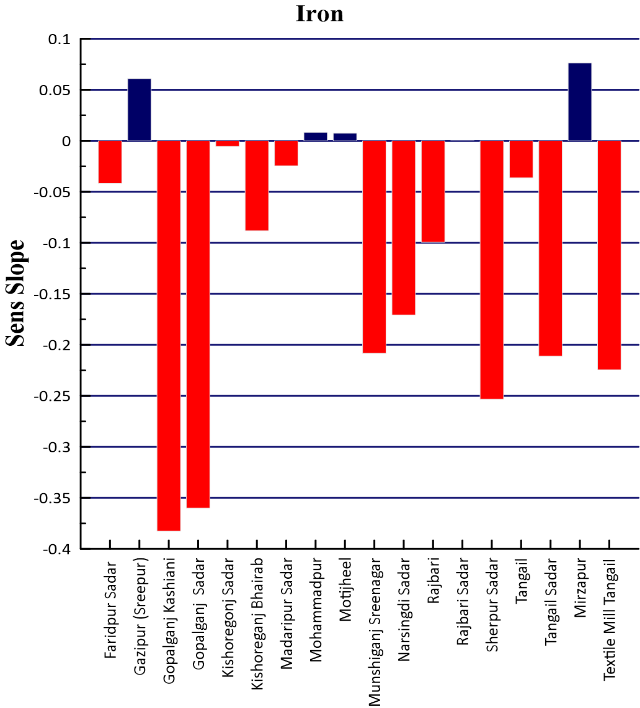


(8) Magnesium

(7) Iron


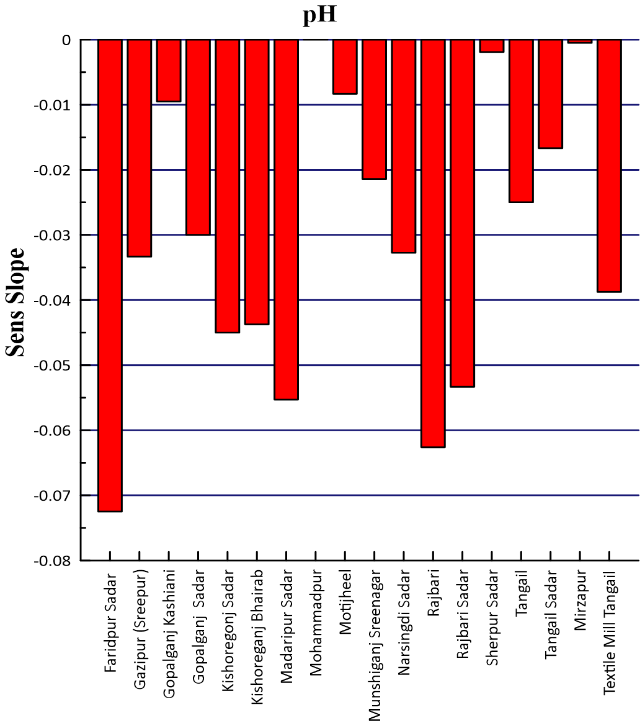

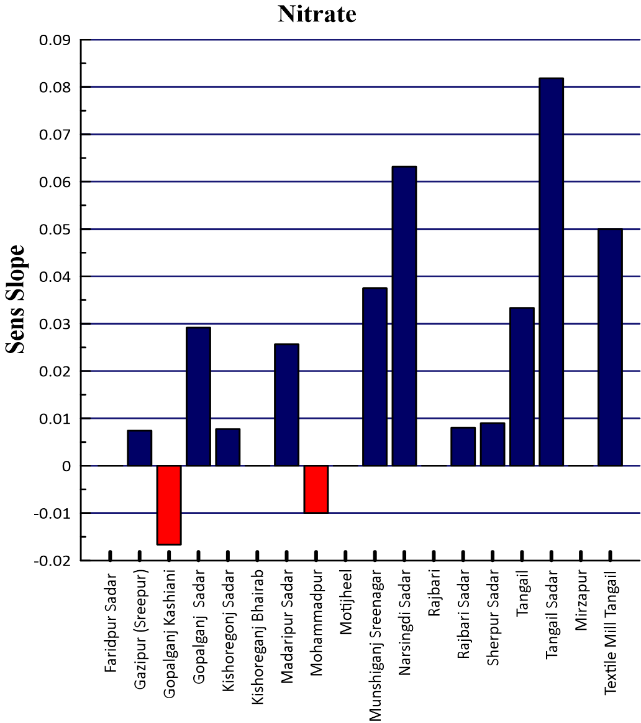


(10) PH

(9) Nitrate


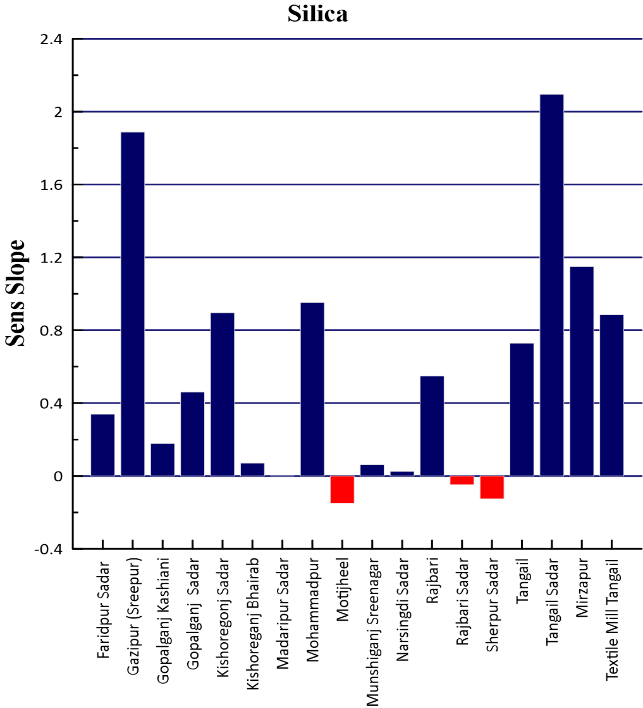

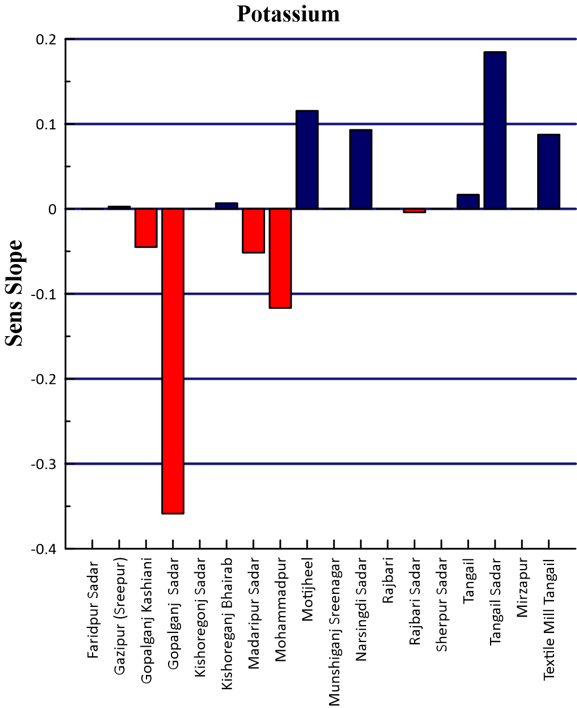


(12) Silica

(11) Potasium


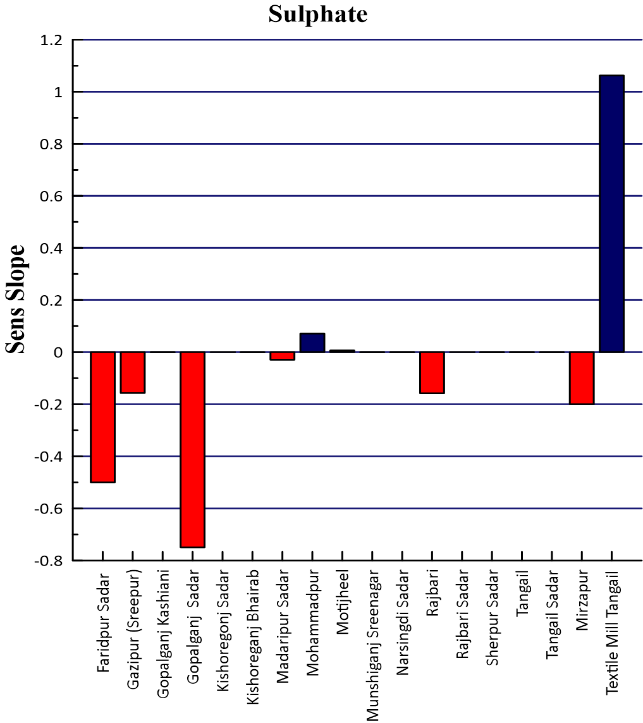

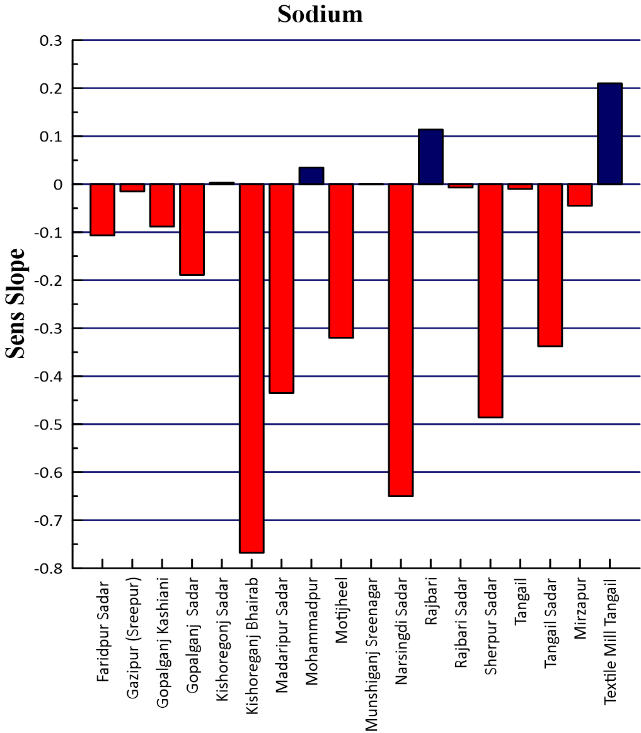


**Figure A4 Bar diagram of Sen’s slope**

(14) Sulphate

(13) Sodium

Appendix A5


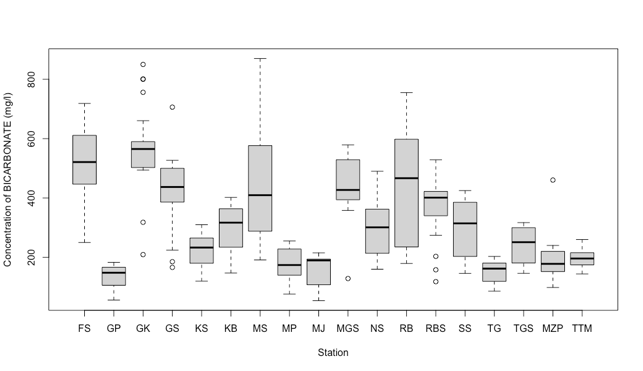


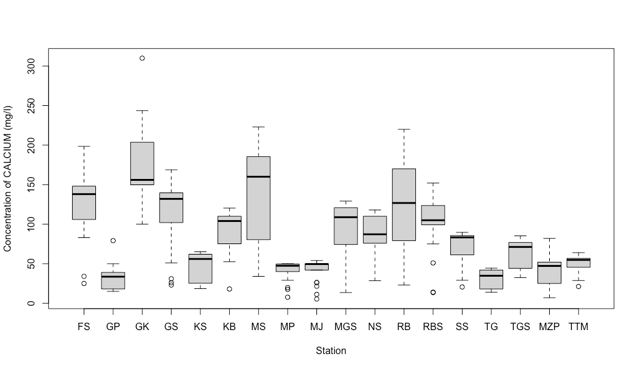


(2) Calcium

(1) Bicarbonate


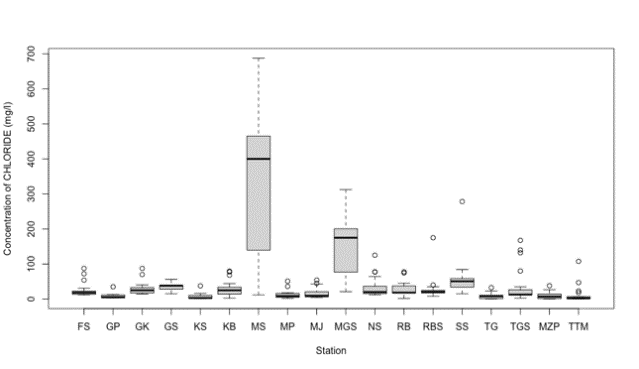

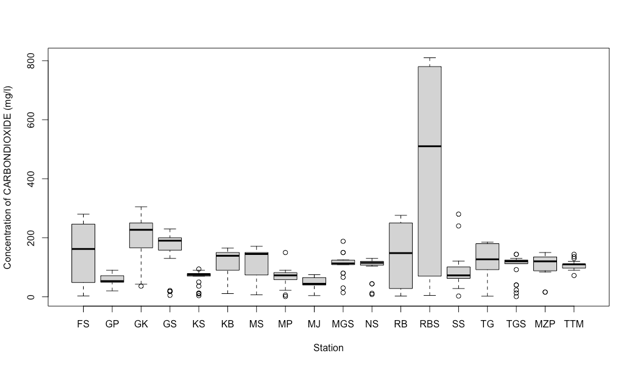


(4) Chloride

(3) Carbondioxide


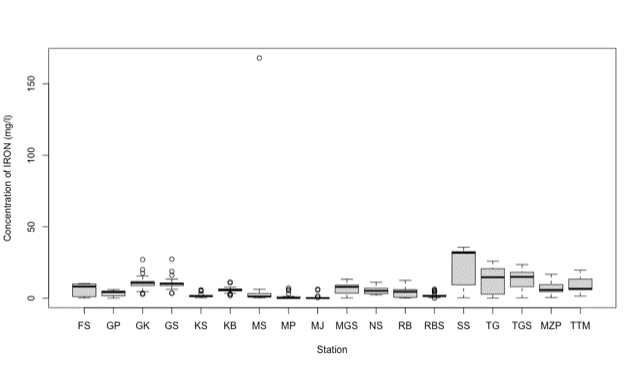

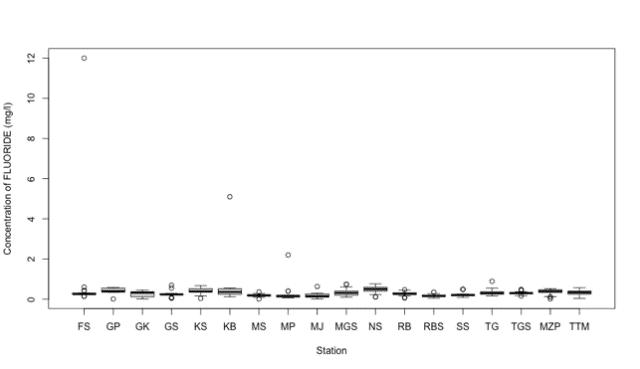


(6) Iron

(5) Fluoride


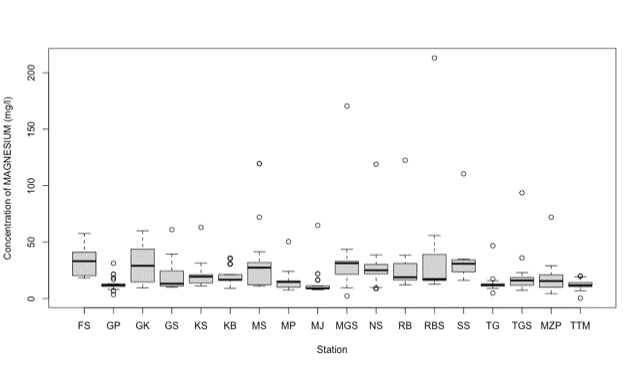


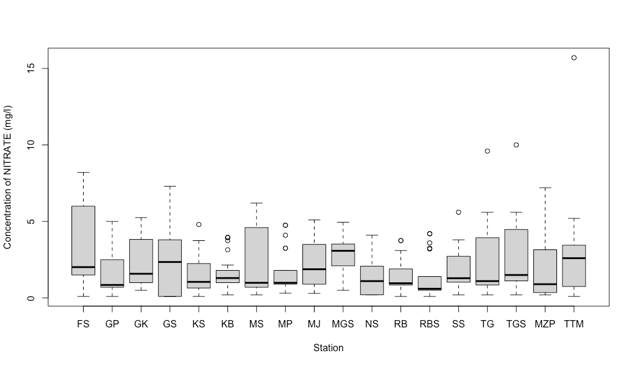


(8) Nitrate

(7) Magnesium


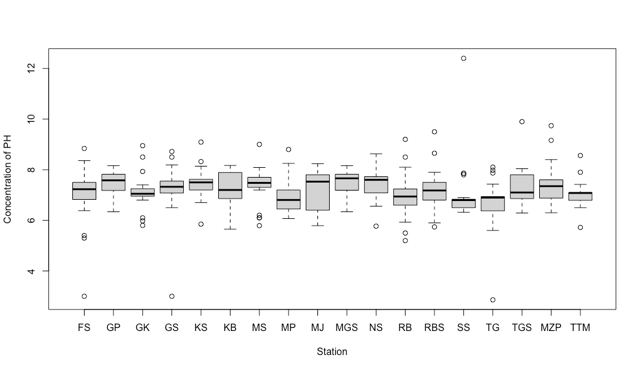


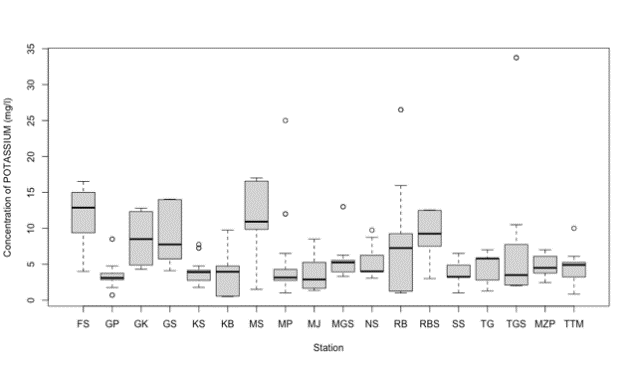


(10) Potassium

(9) PH


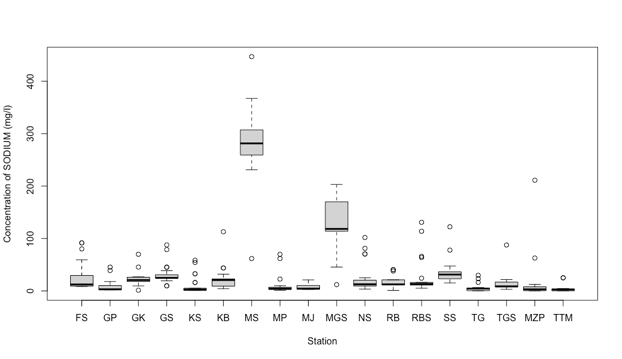

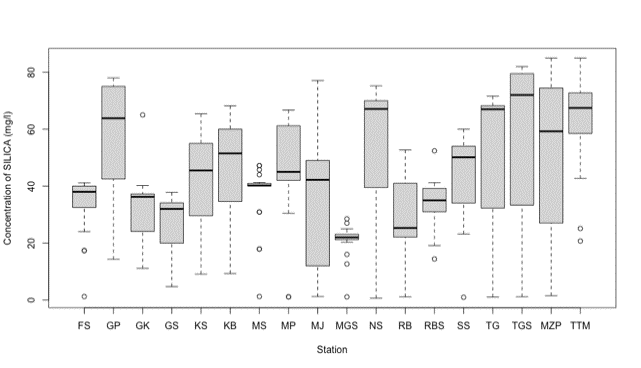


(12) Sodium

(11) Silica


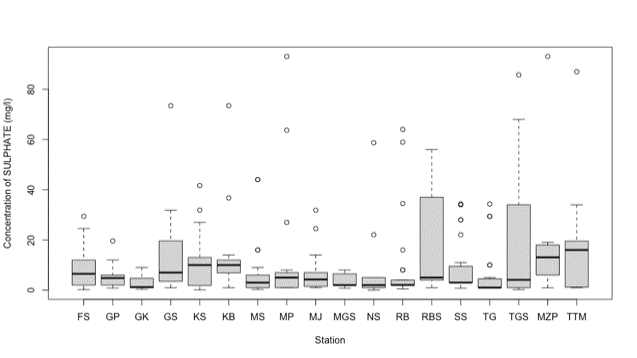


(13) Sulphate

**Figure A5 Box Whiskers plot**
